# Design, synthesis, and *in silico* studies of new benzofuran–pyrazole hybrids as multi-kinase inhibitors with potential antiproliferative activity

**DOI:** 10.1039/d5ra00553a

**Published:** 2025-10-01

**Authors:** Somaia S. Abd El-Karim, Yasmin M. Syam, Reham M. Abdelkader, Mohamed K. El-Ashrey, Manal M. Anwar

**Affiliations:** a Department of Therapeutic Chemistry, National Research Centre El-Bohouth St. 12262 Cairo Egypt ssabdelkarim@gmail.com manal.hasan52@live.com; b Department of Pharmacology and Toxicology, Faculty of Pharmacy and Biotechnology, German University in Cairo Cairo Egypt; c Pharmaceutical Chemistry Department, Faculty of Pharmacy, Cairo University Kasr Elini St. Cairo 11562 Egypt; d Medicinal Chemistry Department, Faculty of Pharmacy, King Salman International University (KSIU) South Sinai 46612 Egypt

## Abstract

A new series of benzofuran–pyrazole-based analogues, conjugated with different substituted aromatic and heterocyclic ring systems featuring the pharmacophoric fragments of protein kinase suppressors, 3a–d and 4a–d was synthesized as potential antiproliferative agents. All the new analogues were selected by the NCI to screen their antiproliferative activity against sixty human cancer cell lines (NCI60). The 1*H*-benzo[*d*]imidazole derivative 3d demonstrated the highest percentage inhibition for various cancer cell lines and advanced to the five-dose assay. It showed potent anti-proliferative activity against various types of cancer lines with GI_50_ values ranging from 0.33 to 4.87 μM and LC_50_ values exceeding 100 μM against the majority of the tested cell lines, confirming its non-lethal effects. Additionally, 3d exhibited multi-targeting PK-suppression activity against B-Raf (V600E), c-Met, Pim-1, EGFR (WT), and VEGFR-2, with IC_50_ values of 0.078 ± 0.004, 0.405 ± 0.017, 1.053 ± 0.046, 0.177 ± 0.007 and 0.275 ± 0.011 μg mL^−1^, respectively. Moreover, 3d caused cell cycle arrest at the G0–G1 phase besides early and late apoptosis in MCF-7 cancer cells. *In silico* molecular docking and ADMET studies were performed on 3d to determine its expected binding interactions with the key regions in the kinase domains, as well as to ascertain its risks of human toxicity, drug-likeness traits, and oral bioavailability.

## Abstract 2

Design and synthesis of novel benzofuran–pyrazole-based analogues conjugated with substituted aromatic and heterocyclic ring systems. 3d exhibited multi-kinase suppression activity against B-Raf (V600E), c-Met, Pim-1, EGFR (WT), and VEGFR-2.

## Graphical abstract

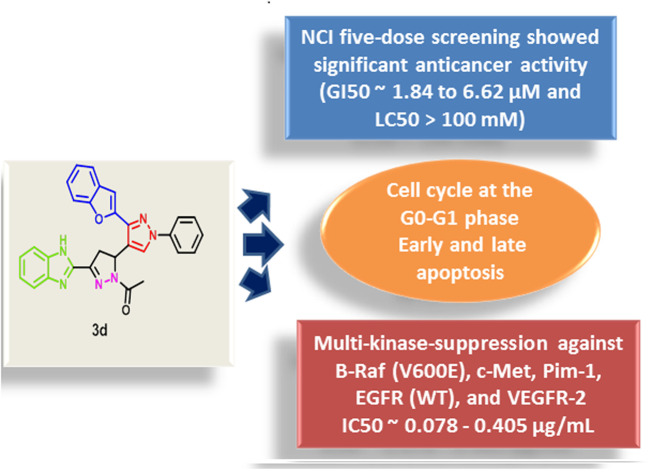

## 1. Introduction

The development of effective and specifically targeted anticancer medicines is the primary goal in cancer therapeutic approaches. Chemotherapy continues to be the most essential means for treating cancer, even with the availability of several clinical cancer treatment techniques, including laser therapy, stem-cell transplantation, radiotherapy, immunotherapy, hormonal therapy, and surgery.^1,2^

However, traditional cancer chemotherapeutic agents lack selectivity, resulting in toxicity to normal cells due to the similarities between cancer and normal human cells. Another emerging challenge in antitumor therapy is the development of tumor cells' resistance to anticancer drugs.^3,4^ In addition, cancer is a heterogeneous disease, and thus, a multitargeted treatment may provide greater therapeutic effectiveness and a lower adverse effect profile than mono-targeted treatment.^5,6^ However, using more than one medication in cancer treatment leads to the simultaneous blocking of different targets, which might result in harmful drug–drug interactions and drug resistance. Therefore, experts believe that employing a single medication capable of influencing multiple targets presents a distinct strategy for surmounting previous obstacles.^7,8^

In humans, protein kinases (PKs) comprise the fifth-largest protein family,^9,10^ which act by transferring the terminal phosphate group of an ATP molecule to a substrate protein.^9,11,12^ These PKs are categorized as tyrosine kinases; serine/threonine kinases; or dual-specificity kinases, such as MEK1 and MEK2, which can catalyze the phosphorylation of tyrosine or threonine on the target proteins.^9,10^ As a result, regulatory disturbance in PKs causes a variety of diseases, including cancer.^13,14^ Thus, blocking PKs is a potential tactic to mitigate the interruptions they cause.^9,15^ PK inhibitors are a well-known type of targeted chemotherapy used in oncology, and they mainly affect the microenvironment and signaling pathways of cancer cells while having few negative effects on healthy cells.^16–18^

The surface of cancerous cells overexpresses a class of tyrosine kinases known as human epithelial growth factor receptors (EGFR). Given that it is implicated in the proliferation, migration, differentiation, apoptosis, and angiogenesis of cancer cells, EGFR (HER1) is one of the most significant cancer treatment targets.^19–22^ Due to T790M, L858R, and C797S mutations in the ATP binding pocket of EGFR, predominant drug resistance and limited drug efficacy were detected in 50% of cancer patients. Accordingly, various generations of EGFR inhibitors have been developed to overcome these resistant tumor clones. However, despite the excellent characteristics of third-generation EGFR inhibitors such as WZ4002, rociletinib and osimertinib (AZD9291), they exhibited several adverse effects in clinical application, including diarrhea, rash, decreased appetite and cardiotoxicity.^23–28^ Accordingly, there is still a great demand to develop new EGFR inhibitors with high selectivity^19,20,29^ (Fig. S24, SI).

The vascular endothelial growth factor (VEGF) family and hepatocyte growth factor receptor (HGFR), which is also known as c-Met (c-mesenchymal epithelial transition factor), are receptor tyrosine kinases (RTKs). Enhanced vascular permeability, endothelial growth, invasion, migration, cell proliferation, differentiation, apoptosis, and morphogenesis are primarily triggered by VEGFR-2 and c-Met. Moreover, VEGFR-2 is critical in pathological and physiological angiogenesis. Therefore, experts believe that directly stopping the intracellular kinase domains of VEGFR-2 and c-Met by competing with the ATP-binding sites is the most effective way to stop tumor growth^15,29–49^ (S1, SI).

Three serine/threonine kinases (Pim-1, Pim-2, and Pim-3) make up the provirus integration in Maloney (Pim) kinases family. They play a crucial role in controlling several biological processes, such as the cell cycle, apoptosis, and proliferation.^50^ Given that PIM kinases are expressed in a variety of solid and hematological malignancies and are essentially absent in benign tumors, they have been shown to be effective targets for anticancer drugs with low toxicity.^51^ Most Pim inhibitor investigations have focused on Pim-1 inhibitors because of the low *K*_m_ of Pim-2 for ATP, which is 100-times lower than that of Pim-1 and Pim-3 (S1, SI).^52,53^ Additionally, the ERK–MAPK pathway is regulated by serine/threonine kinases called BRAF. Due to the replacement of valine for glutamic acid at position 600, the BRAF gene (V600E) has the highest frequency of BRAF mutations in human malignancies. According to recent studies, BRAF suppression marks a new era in the therapeutic management of human cancer^54^ (S1, SI).

**Fig. 1:**
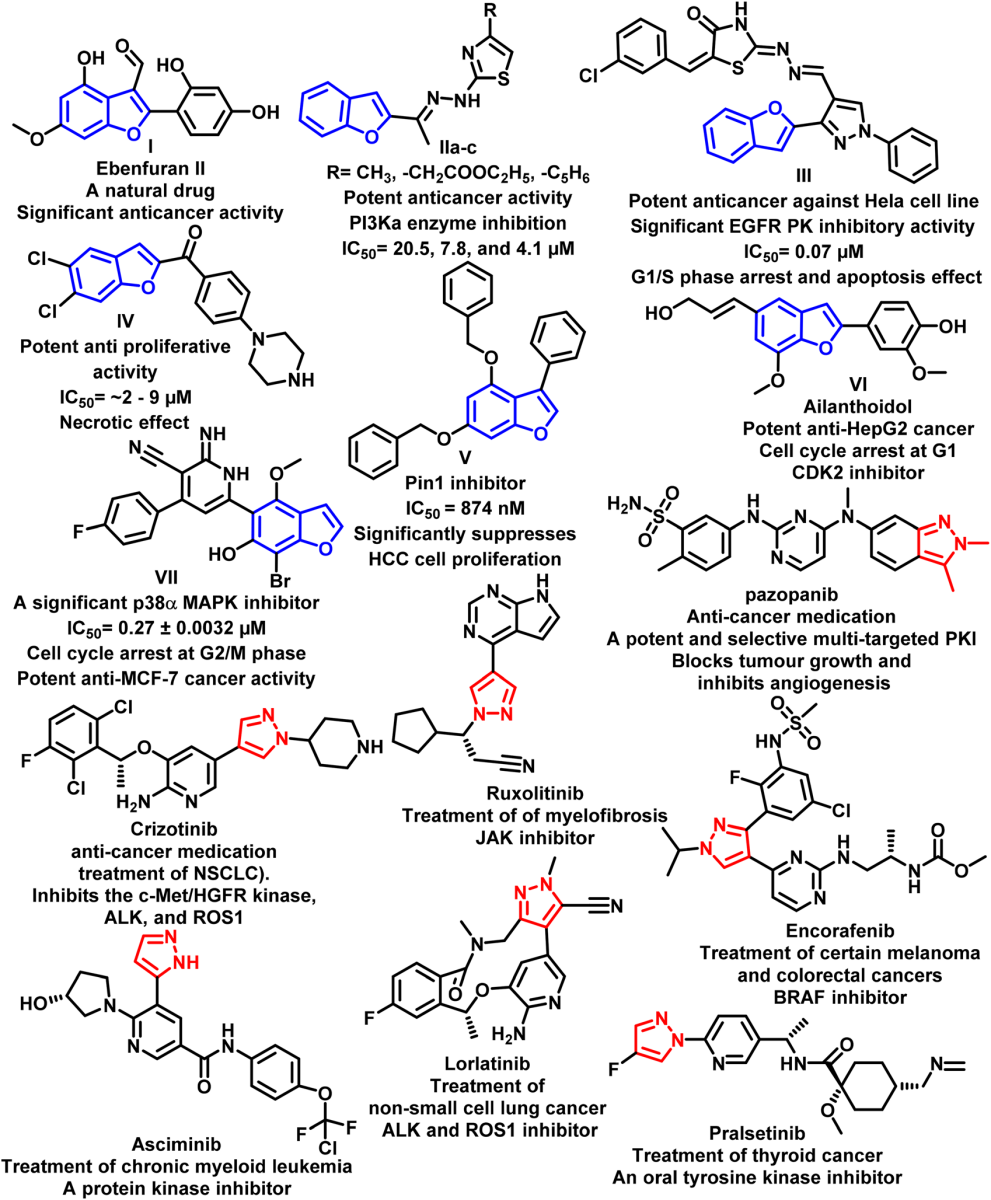
Examples of various benzofuran- and pyrazole-based candidates with anticancer activity targeting different protein kinases.

The benzofuran nucleus is a useful building block in the realm of pharmaceutical discovery and development due to its intriguing biological characteristics, especially in the field of cancer treatment. This scaffold has various approaches to mediate its anticancer action, such as anti-angiogenesis, antitubulin polymerization, and inhibition of carbonic anhydrases, EGFR, and different protein kinases.^16,55,56^Fig. 1 demonstrates various examples of benzofuran-based derivatives with significant anticancer activity targeting different protein kinases.^16,56–58^

Azoles, *viz.*, pyrazoles and pyrazolines, constitute valuable building blocks in medicinal chemistry.^15^ Presently, different commercially available pyrazole-containing drugs are used to treat various types of cancers (Fig. 1).^59–62^ Several studies demonstrated that pyrazole-based compounds exhibit *in vitro* and *in vivo* antitumor activity by inhibiting various enzymes such as topoisomerase II, EGFR, MEK, VEGFR, GGT1, microtubule, and HDACs.^59–62^

### 1.1. Rationale of molecular design

Molecular hybridization is an effective technique in the realm of drug discovery and development, where this approach aims to conjugate two or more pharmacophoric or bioactive subunits covalently into novel hybrid molecules. In comparison to their parent drugs, these newly created hybrids often exhibit better affinity and efficacy, improved pharmacokinetic and pharmacodynamic properties, and the ability to engage in dual or multiple modes of action, with diminished undesirable side effects and reduced likelihood of drug–drug interactions and emergence of drug resistance or proliferation.^63–67^

**Fig. 2:**
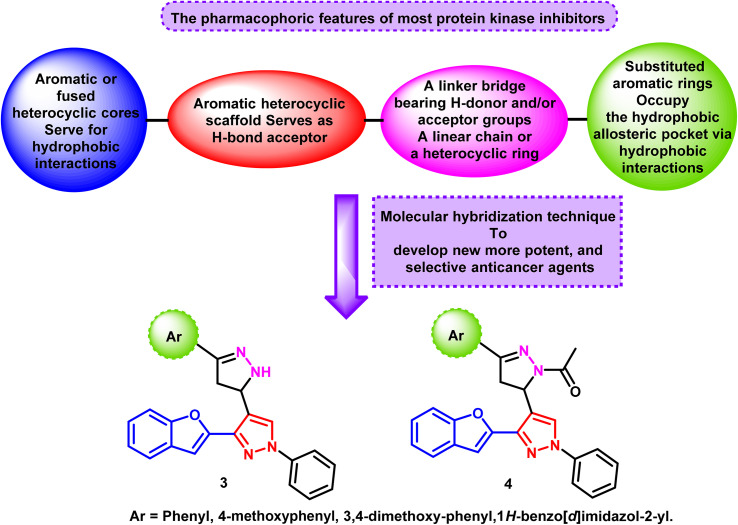
Hypothetical pharmacophoric characteristics of the newly synthesized compounds.

The majority of studies that link the molecular structures of various heterocyclic-based derivatives and their ability to suppress protein kinases have revealed that these compounds have four main characteristic features, a central aromatic heterocyclic scaffold, which serves as a hydrogen bond acceptor (HBA) to interact with the adenine binding pocket of the target enzyme, an aryl ring, such as substituted phenyl, aromatic, and fused aromatic heterocyclic cores, a linker bridge that might be a linear chain or a heterocyclic moiety (where the length and number of hydrogen donor and/or acceptor groups can be altered), and a hydrophobic tail, such as a phenyl ring, which occupies the hydrophobic allosteric pocket through multiple hydrophobic interactions^15,68^ (Fig. 2).

As a result of our curiosity in the field of drug discovery and based on the promising anticancer potency of the benzofuran and pyrazole ring systems, we have utilized the molecular hybridization technique to design and create new compounds that possess the key pharmacophoric characteristics of protein kinase suppressors with multi-targeting kinase inhibition and anticancer potential. The new congeners are based on a benzofuran–pyrazole scaffold as the main heteroaromatic system to interact with the adenine binding pocket of the target enzyme tethered with various substituted aromatic and heterocyclic rings *via* pyrazoline rings 3 and 4 (Fig. 2). The degree of hydrophobicity and releasing/withdrawing capacities of the substituents on the pyrazoline ring produced various impacts on inhibitory action against the target protein kinase and the anticancer activity.

The National Cancer Institute (NCI) tested all the new target candidates for their anticancer activity against a set of sixty cancer cell lines. Moreover, the impact of the most promising compound was further evaluated *in vitro* against multi-kinases, B-Raf (V600E), c-Met, Pim-1, EGFR (WT), and VEGFR-2 enzymes, in addition to the cancer cell cycle and the capacity to trigger apoptosis to reveal the anticipated mechanism of anti-proliferative activity.

*In silico* molecular docking investigation was also carried out to find out the expected interaction model of the most promising analogue with the active sites of the above-mentioned kinases. Furthermore, an *in silico* ADMET study was performed to ascertain its risks of human toxicity, drug-likeness traits, and oral bioavailability.

## 2. Materials and methods

### 2.1. Chemistry

#### 2.1.1. General remarks

The chemicals that were involved in the synthesis of the target compounds, as well as for biological evaluation were obtained from commercial suppliers. Precoated silica gel 60 F_245_ aluminium plates (Merck) were used to follow the progress of the chemical reaction. Uncorrected melting points were measured on an Electrothermal 9100 apparatus. Spectral and elemental analyses of the synthesized compounds were performed in the Micro Analytical Labs, National Research Centre, Cairo, Egypt. IR spectra (4000–400 cm^−1^) were recorded on an FT/IR-4100 Jasco-Japan Fourier transform infrared spectrophotometer. ^1^H NMR and ^13^C NMR (DMSO-*d*_6_) spectra were measured at 400 (100) MHz on Bruker instruments.

#### 2.1.2. General procedure for the synthesis of the target compounds 3-(3-(benzofuran-2-yl)-1-phenyl-1*H*-pyrazol-4-yl)-1-(substituted)-prop-2-en-1-one 2a–d

A mixture of carbaldehyde 1 (2.88 g, 0.01 mol) and the appropriate acetyl derivatives, namely, acetophenone, 4-methoxyacetophenone, 3,4-dimethoxy acetophenone and/or 2-acetyl-1*H*-benzo[*d*]imidazole (0.01 mol), in alcoholic sodium hydroxide solution (30 mL, 10%) was stirred for 5 h. The reaction mixture was left overnight at room temperature. The formed precipitate was filtered, washed several times with water, dried and recrystallized from ethanol to give the target compounds 2a–d, respectively.

##### 2.1.2.1. 3-(5-(Benzofuran-2-yl)-1-phenyl-1*H*-pyrazol-3-yl)-1-phenylprop-2-en-1-one (2a)

Yield (75%), mp 201–203 °C, IR (*ν*_max_/cm^−1^): 3062 (CH, aromatic), 2916, 2851 (CH-aliphatic), 1632 (C=O), 1601 (C=C); ^1^HNMR (400 MHz; DMSO-*d*_6_) *δ*_H_ 7.35 (t, ^3^*J* = 7.18 Hz, 1H, Ar–H), 7.40–7.48 (m, 3H, Ar–H), 7.63 (t, ^3^*J* = 7.10 Hz, 4H, Ar–H), 7.71 (t, ^3^*J* = 6.48 Hz, ^4^*J* = 2.32 Hz, 2H, Ar–H), 7.77 (d, ^3^*J* = 7.40 Hz, 1H, CH=), 7.94–7.99 (m, 3H, Ar–H), 8.14–8.17 (m, 3H, Ar–H), 9.52 (s, 1H, pyrazole-H4) ppm; ^13^C NMR (100 MHz; DMSO-*d*_6_) *δ*_C_ 105.82, 111.73, 118.96, 119.36, 122.15, 122.86, 124.02, 125.74, 128.08, 128.40, 128.77, 129.30, 129.47, 130.25, 133.60, 134.23, 138.11, 139.19, 143.67, 149.60, 154.80, 189.40 (CO) ppm; MS, *m*/*z* (%): 390 [M˙^+^] (38.05), analysis for C_26_H_18_N_2_O_2_ (390.44), calcd% C, 79.98; H, 4.65; N, 7.17 found: % C, 97.75; H, 4.58; N, 7.08.

##### 2.1.2.2. 3-(5-(Benzofuran-2-yl)-1-phenyl-1*H*-pyrazol-3-yl)-1-(4-methoxyphenyl)prop-2-en-1-one (2b)

Yield (75%), mp 125–126 °C, IR (*ν*_max_/cm^−1^): 3063 (CH, aromatic), 2955, 2832 (CH-aliphatic), 1639 (C=O), 1601 (C=C); ^1^HNMR (400 MHz; DMSO-*d*_6_) *δ*_H_ 3.89 (s, 3H, OCH_3_), 7.13 (d, ^3^*J* = 8.72 Hz, 2H, Ar–H), 7.35 (t, ^3^*J* = 7.18 Hz, 1H, Ar–H), 7.40–7.42 (m, 2H, Ar–H), 7.46 (t, ^3^*J* = 7.40 Hz, 1H, Ar–H), 7.63 (t, ^3^*J* = 7.92 Hz, 2H, Ar–H), 7.72 (d, ^3^*J* = 8.16 Hz, 1H, =CH), 7.77 (d, ^3^*J* = 7.28 Hz, 1H, =CH), 7.95–7.99 (m, 3H, Ar–H), 8.09–8.17 (m, 3H, Ar–H), 9.49 (s, 1H, pyrazole-H4) ppm. ^13^C NMR (100 MHz; DMSO-*d*_6_) *δ*_C_ 56.05 (OCH_3_), 105.77, 111.73, 114.53, 119.08, 119.34, 122.13, 122.81, 124.01, 125.72, 128.04, 128.41, 129.26, 130.24, 130.90, 131.16, 133.19, 139.20, 143.57, 149.62, 154.78, 163.71, 187.56 (CO) ppm; MS, *m*/*z* (%): 420 [M˙^+^] (33.47), analysis for C_27_H_20_N_2_O_3_ (420.47), calcd% C, 77.13; H, 4.79; N, 6.66 found: % C, 77.19; H, 4.83; N, 6.72.

##### 2.1.2.3. 3-(5-(Benzofuran-2-yl)-1-phenyl-1*H*-pyrazol-3-yl)-1-(3,4-dimethoxyphenyl)prop-2-en-1-one (2c)

Yield (75%), mp 175–176 °C, IR (*ν*_max_/cm^−1^): 3063 (CH, aromatic), 2923, 2839 (CH-aliphatic), 1651 (C=O), 1597 (C=C); ^1^HNMR (400 MHz; DMSO-*d*_6_) *δ*_H_ 3.87 (s, 3H, OCH_3_), 3.90 (s, 3H, OCH_3_), 7.15 (d, ^3^*J* = 8.48 Hz, 1H, Ar–H), 7.35 (t, ^3^*J* = 7.32 Hz, 1H, Ar–H), 7.40–4.42 (m, 1H, Ar–H), 7.46 (t, ^3^*J* = 7.36 Hz, 2H, Ar–H), 7.61–7.65 (m, 3H, Ar–H), 7.71 (d, ^3^*J* = 8.12 Hz, 1H, =CH), 7.77 (d, ^3^*J* = 7.48 Hz, 1H, =CH), 7.86 (d, ^3^*J* = 8.44 Hz, ^4^*J* = 1.72 Hz, 1H, Ar–H), 7.92–7.93 (m, 1H, Ar–H), 7.97 (d, ^3^*J* = 8.76 Hz, 2H, Ar–H), 8.09–8.13 (m, 1H, Ar–H), 9.47 (s, 1H, pyrazole-H4) ppm. ^13^C NMR (100 MHz; DMSO-*d*_6_) *δ*_C_ 56.08 (OCH_3_), 56.29 (OCH_3_), 105.76, 111.21, 111.36, 111.73, 119.11, 119.39, 122.13, 122.79, 123.54, 124.01, 125.71, 128.03, 128.43, 129.33, 130.24, 131.01, 133.12, 139.24, 143.57, 149.36, 149.69, 153.75, 154.80, 187.47 (CO) ppm; MS, *m*/*z* (%): 450 [M˙^+^] (37.06), analysis for C_28_H_22_N_2_O_4_ (450.49), calcd% C, 74.65; H, 4.92; N, 6.22 found: % C, 74.70; H, 4.87; N, 6.38.

##### 2.1.2.4. 1-(1*H*-Benzo[*d*]imidazol-2-yl)-3-(5-(benzofuran-2-yl)-1-phenyl-1*H*-pyrazol-3-yl)prop-2-en-1-one (2d)

Yield (75%), mp 222–223 °C, IR (*ν*_max_/cm^−1^): 3244 (NH), 3063 (CH, aromatic), 2924, 2851 (CH-aliphatic), 1655 (C=O),1582 (C=C); ^1^HNMR (400 MHz; DMSO-*d*_6_) *δ*_H_ 7.33–7.37 (m, 3H, Ar–H), 7.41–7.46 (m, 3H, Ar–H), 7.60 (t, ^3^*J* = 7.92 Hz, 2H, Ar–H), 7.72–7.76 (m, 3H, Ar–H), 7.79 (d, ^3^*J* = 7.52 Hz, 1H, Ar–H), 8.07 (d, ^3^*J* = 7.84 Hz, 2H, Ar–H), 8.18 (d, ^3^*J* = 16.0 Hz, 1H, Ar–H), 8.42 (d, ^3^*J* = 16.0 Hz, 1H, Ar–H), 9.62 (s, 1H, pyrazole-H4) ppm. ^13^C NMR (100 MHz; DMSO-*d*_6_) *δ*_C_ 106.06, 111.80, 117.52, 118.82, 119.44, 122.16, 122.85, 124.03, 124.69, 125.76, 128.02, 128.43, 129.67, 130.15, 134.59, 139.19, 143.91, 149.52, 149.98, 154.85, 181.59 (CO) ppm; MS, *m*/*z* (%): 430 [M˙^+^] (33.63), analysis for C_27_H_18_N_4_O_2_ (430.47), calcd% C, 75.34; H, 4.21; N, 13.02 found: % C, 75.47; H, 4.28; N, 12.95.

#### 2.1.3. General procedure for the synthesis of the target compounds 3-(benzofuran-2-yl)-4-(3-substituted-4,5-dihydro-1*H*-pyrazol-5-yl)-1-phenyl-1*H*-pyrazole 3a–d

A mixture of chalcones 2a–d (0.002 mol) and hydrazine hydrate (0.2 mL, 98%) in absolute ethanol was refluxed for 3 h. The precipitate formed during heating was filtered, dried and recrystallized from absolute ethanol to give compounds 3a–d, respectively.

##### 2.1.3.1. 3-(Benzofuran-2-yl)-4-(3-phenyl-4,5-dihydro-1*H*-pyrazol-5-yl)-1-phenyl-1*H*-pyrazole (3a)

Yield (75%), mp 145–146 °C, IR (*ν*_max_/cm^−1^): 3314 (NH), 3082, 3055 (CH, aromatic), 2920, 2851 (CH-aliphatic), 1597 (C=C), ^1^HNMR (400 MHz; DMSO-*d*_6_) *δ*_H_ 3.04–3.11 (dd, ^2^*J* = 10.4, ^3^*J* = 16.28 Hz, 1H, pyrazoline-H4), 3.59–3.65 (dd, ^2^*J* = 10.72, ^3^*J* = 16.28 Hz, 1H, pyrazoline-H4), 5.24–5.30 (m, 1H, pyrazoline-H5), 7.28–7.42 (m, 7H, Ar–H), 7.52–7.59 (m, 3H, Ar–H), 7.67–7.72 (m, 3H, Ar–H), 7.93 (d, ^3^*J* = 7.88 Hz, 2H, Ar–H), 8.64 (s, 1H, pyrazole-H4), 10.21 (s, 1H, NH, D_2_O exchangeable) ppm. ^13^C NMR (100 MHz; DMSO-*d*_6_) *δ*_C_ 50.50 (pyrazoline-C-4), 55.47 (pyrazoline-C5), 95.99, 104.78, 109.65, 111.77, 118.88, 119.76, 121.78, 123.76, 124.64, 125.22, 126.04, 127.17, 128.76, 128.97, 129.35, 130.12, 130.27, 133.63, 134.00, 139.66, 141.86, 150.04, 154.52, 154.62 (Ar–C) ppm; MS, *m*/*z* (%): 390 [M˙^+^] (38.05), analysis for C_26_H_20_N_4_O (404.47), calcd% C, 77.21; H, 4.98; N, 13.85 found: % C, 77.32; H, 5.03; N, 13.90.

##### 2.1.3.2. 3-(Benzofuran-2-yl)-4-(3-(4-methoxyphenyl)-4,5-dihydro-1*H*-pyrazol-5-yl)-1-phenyl-1*H*-pyrazole (3b)

Yield (75%), mp 172–173 °C, IR (*ν*_max_/cm^−1^): 3310 (NH), 3078, 3051 (CH, aromatic), 2928, 2835 (CH-aliphatic), 1601 (C=C); ^1^HNMR (400 MHz; DMSO-*d*_6_) *δ*_H_ 3.00–3.07 (dd, ^2^*J* = 10.48, ^3^*J* = 16.20 Hz, 1H, pyrazoline-H4), 3.56–3.62 (dd, ^2^*J* = 10.56, ^3^*J* = 16.24 Hz, 1H, pyrazoline-H4), 3.78 (s, 3H, OCH_3_), 5.23 (t, ^3^*J* = 10.52 Hz, 1H, pyrazoline-H5), 6.95 (d, ^3^*J* = 8.84 Hz, 2H, Ar–H), 7.28 (t, ^3^*J* = 7.86 Hz, 1H, Ar–H), 7.32–7.38 (m, 4H, Ar–H), 7.54 (t, ^3^*J* = 7.94 Hz, 2H, Ar–H), 7.61 (d, ^3^*J* = 8.80 Hz, 2H, Ar–H), 7.70 (t, ^3^*J* = 7.04 Hz, 2H, Ar–H), 7.92 (d, ^3^*J* = 7.84 Hz, 2H, Ar–H), 8.63 (s, 1H, pyrazole-H); MS, *m*/*z* (%): 434 [M˙^+^] (35.62), analysis for C_27_H_22_N_4_O_2_ (434.50), calcd% C, 74.64; H, 5.10; N, 12.89 found: % C, 74.72; H, 5.23; N, 12.91.

##### 2.1.3.3. 3-(Benzofuran-2-yl)-4-(3-(3,4-dimethoxyphenyl)-4,5-dihydro-1*H*-pyrazol-5-yl)-1-phenyl-1*H*-pyrazole (3c)

Yield (75%), mp 158–160 °C, IR (*ν*_max_/cm^−1^): 3314 (NH), 3067, 3051 (CH, aromatic), 2920, 2851 (CH-aliphatic), 1597 (C=C); ^1^HNMR (400 MHz; DMSO-*d*_6_) *δ*_H_ 3.02–3.08 (dd, ^2^*J* = 10.32, ^3^*J* = 16.20 Hz, 1H, pyrazoline-H4), 3.56–3.62 (dd, ^2^*J* = 10.56, ^3^*J* = 16.20 Hz, 1H, pyrazoline-H4), 3.78 (s, 3H, OCH_3_), 3.79 (s, 3H, OCH_3_), 5.24 (t, ^3^*J* = 10.42 Hz, 1H, pyrazoline-H5), 6.95 (d, ^3^*J* = 8.36 Hz, 1H, Ar–H), 7.13 (d, ^3^*J* = 8.32, ^4^*J* = 1.68, 1H, Ar–H), 7.29–7.40 (m, 5H, Ar–H), 7.54 (t, ^3^*J* = 7.92 Hz, 2H, Ar–H), 7.71 (t, ^3^*J* = 6.92 Hz, 2H, Ar–H), 7.93 (d, ^3^*J* = 7.84 Hz, 2H, Ar–H), 8.62 (s, 1H, pyrazole-H4) ppm. ^13^C NMR (100 MHz; DMSO-*d*_6_) *δ*_C_ 42.22 (pyrazoline-C4), 55.43 (pyrazoline-C5), 55.85 (OCH_3_), 55.97 (OCH_3_), 104.79, 108.83, 111.76, 111.87, 118.87, 119.42, 121.78, 123.76, 124.73, 125.22, 126.50, 127.15, 127.73, 128.77, 130.11, 139.67, 141.86, 149.17, 149.75, 150.07, 150.29, 154.51 (Ar–C) ppm; MS, *m*/*z* (%): 464 [M˙^+^] (33.47), analysis for C_28_H_24_N_4_O_3_ (464.53), calcd% C, 72.40; H, 5.21; N, 12.06 found: % C, 72.48; H, 5.36; N, 11.86.

##### 2.1.3.4. 3-(Benzofuran-2-yl)-4-(3-(1*H*-benzo[*d*]imidazol-2-yl)-4,5-dihydro-1*H*-pyrazol-5-yl)-1-phenyl-1*H*-pyrazole (3d)

Yield (75%), mp 218–220 °C, IR (*ν*_max_/cm^−1^): 3390, 3348 (2NH), 3051 (CH, aromatic), 2920, 2851 (CH-aliphatic), 1597 (C=C); ^1^HNMR (400 MHz; DMSO-*d*_6_) *δ*_H_ 3.19–3.26 (dd, ^2^*J* = 10.20, ^3^*J* = 16.60 Hz, 1H, pyrazoline-H4), 3.72–3.79 (dd, ^2^*J* = 11.16, ^3^*J* = 16.60 Hz, 1H, pyrazoline-H4), 5.38 (t, ^3^*J* = 7.84, ^4^*J* = 2.16 Hz, 1H, pyrazoline-H5), 7.14 (t, ^3^*J* = 7.52 Hz, 1H, Ar–H), 7.20 (t, ^3^*J* = 7.36 Hz, 1H, Ar–H), 7.29–7.37 (m, 4H, Ar–H), 7.44 (d, ^3^*J* = 7.84 Hz, 1H, Ar–H), 7.54 (t, ^3^*J* = 7.84 Hz, 2H, Ar–H), 7.60 (d, ^3^*J* = 7.72 Hz, 1H, Ar–H), 7.68 (d, ^3^*J* = 8.04 Hz, 1H, Ar–H), 7.72 (d, ^3^*J* = 7.44 Hz, 1H, Ar–H), 7.94 (d, ^3^*J* = 8.00 Hz, 2H, Ar–H), 8.14 (s, 1H, Ar–H), 8.68 (s, 1H, pyrazole-H), 12.75 (s, 1H, NH, exchangeable with D_2_O) ppm. ^13^C NMR (100 MHz; DMSO-*d*_6_) *δ*_C_ 55.67 (pyrazoline-C4), 56.56 (pyrazoline-C5), 104.78, 111.72, 118.94, 119.24, 119.78, 121.84, 122.84, 123.81, 124.06, 125.30, 127.27, 127.82, 128.70, 130.14, 139.59, 141.85, 147.37, 149.95, 154.53 (Ar–C) ppm; MS, *m*/*z* (%): 464 [M˙^+^] (33.47), analysis for C_27_H_20_N_6_O (444.50), calcd% C, 72.96; H, 4.54; N, 18.91 found: % C, 73.05; H, 4.68; N, 18.97.

#### 2.1.4. General procedure for the synthesis of the target compounds 1-(5-(3-(benzofuran-2-yl)-1-phenyl-1*H*-pyrazol-4-yl)-3-substituted-4,5-dihydropyrazol-1-yl)ethanone 4a–d

A mixture of chalcones 2a–d (0.002 mol) and hydrazine hydrate (0.2 mL, 98%) in glacial acetic acid (15 mL) was refluxed for 3 h. The precipitate formed during heating was filtered, dried and recrystallized from acetic acid to give the target compounds 4a–d, respectively.

##### 2.1.4.1. 1-(5-(3-(Benzofuran-2-yl)-1-phenyl-1*H*-pyrazol-4-yl)-3-phenyl-4,5-dihydropyrazol-1-yl)ethanone (4a)

Yield (75%), mp 171–173 °C, IR (*ν*_max_/cm^−1^): 3097, 3062 (CH, aromatic), 2928, 2851 (CH-aliphatic), 1659 (C=O), 1597 (C=C); ^1^HNMR (400 MHz; DMSO-*d*_6_) *δ*_H_ 2.36 (s, 3H, CH_3_), 3.21–3.29 (m, 1H, pyrazoline-H4), 4.01–4.10 (dd, ^2^*J* = 11.84, ^3^*J* = 17.88 Hz, 1H, pyrazoline-H4), 5.95–5.99 (dd, ^2^*J* = 5.24, ^3^*J* = 11.88 Hz, 1H, pyrazoline-H5), 7.29–7.38 (m, 4H, Ar–H), 7.46–7.48 (m, 3H, Ar–H), 7.52 (t, ^3^*J* = 7.94 Hz, 2H, Ar–H), 7.60 (d, ^3^*J* = 8.00 Hz, 1H, Ar–H), 7.71 (d, ^3^*J* = 7.04 Hz, 1H, Ar–H), 7.80–7.83 (m, 2H, Ar–H), 7.92 (d, ^3^*J* = 7.88 Hz, 2H, Ar–H), 8.47 (s, 1H, pyrazole-H) ppm. ^13^C NMR (100 MHz; DMSO-*d*_6_) *δ*_C_ 22.35 (CH_3_), 42.23 (pyrazoline-C-4), 52.37 (pyrazoline-C5), 104.59, 111.70, 118.91, 121.82, 123.81, 124.21, 125.22, 127.12, 127.23, 127.42, 128.68, 129.22, 130.03, 130.76, 131.65, 139.49, 140.88, 149.94, 154.53, 154.80, 168.38 (CO) ppm; MS, *m*/*z* (%): 446 [M˙^+^] (30.06), analysis for C_28_H_22_N_4_O_2_ (446.51), calcd% C, 75.32; H, 4.97; N, 12.55 found: % C, 75.47; H, 5.06; N, 12.58.

##### 2.1.4.2. 1-(5-(3-(Benzofuran-2-yl)-1-phenyl-1*H*-pyrazol-4-yl)-3-(4-methoxyphenyl)-4,5-dihydropyrazol-1-yl)ethanone (4b)

Yield (75%), mp 214–215 °C, IR (*ν*_max_/cm^−1^): 3048 (CH, aromatic), 2928, 2835 (CH-aliphatic), 1651 (C=O), 1609 (C=C); ^1^HNMR (400 MHz; DMSO-*d*_6_) *δ*_H_ 2.35 (s, 3H, CH_3_), 3.22–3.30 (m, 1H, pyrazoline-H4), 3.80 (s, 3H, OCH_3_), 3.98–4.06 (dd, ^2^*J* = 11.84, ^3^*J* = 17.80 Hz, 1H, pyrazoline-H4), 5.92–5.96 (dd, ^2^*J* = 5.08, ^3^*J* = 11.76 Hz, 1H, pyrazoline-H5), 7.00 (d, ^3^*J* = 8.80 Hz, 2H, Ar–H), 7.29–7.38 (m, 4H, Ar–H), 7.52 (t, ^3^*J* = 7.88 Hz, 2H, Ar–H), 7.62 (d, ^3^*J* = 7.96 Hz, 1H, Ar–H), 7.71–7.77 (m, 3H, Ar–H), 7.92 (d, ^3^*J* = 7.92 Hz, 2H, Ar–H), 8.44 (s, 1H, pyrazole-H) ppm; ^13^C NMR (100 MHz; DMSO-*d*_6_) *δ*_C_ 22.34 (CH_3_), 42.33 (pyrazoline-C-4), 52.24 (pyrazoline-C-5), 55.79 (OCH_3_), 104.57, 111.72, 114.63, 118.90, 121.81, 123.80, 124.18, 124.33, 125.22, 127.19, 127.31, 128.70, 128.81, 130.02, 139.51, 140.86, 149.96, 154.53, 161.34, 168.08, 168.63, 172.58 (CO) ppm; MS, *m*/*z* (%): 476 [M˙^+^] (28.72), analysis for C_29_H_24_N_4_O_3_ (476.54), calcd% C, 73.09; H, 5.08; N, 11.76 found: % C, 73.15; H, 5.12; N, 11.82.

##### 2.1.4.3. 1-(5-(3-(Benzofuran-2-yl)-1-phenyl-1*H*-pyrazol-4-yl)-3-(3,4-dimethoxyphenyl)-4,5-dihydropyrazol-1-yl)ethanone (4c)

Yield (75%), mp 217–218 °C, IR (*ν*_max_/cm^−1^): 3097, 3050 (CH, aromatic), 2932, 2835 (CH-aliphatic), 1659 (C=O), 1601 (C=C); ^1^HNMR (400 MHz; DMSO-*d*_6_) *δ*_H_ 2.36 (s, 3H, CH_3_), 3.01–3.25 (m, 1H, pyrazoline-H4), 3.80 (s, 3H, OCH_3_), 3.81 (s, 3H, OCH_3_), 3.97–4.05 (dd, ^2^*J* = 11.72, ^3^*J* = 17.64 Hz, 1H, pyrazoline-H4), 5.94–5.98 (dd, ^2^*J* = 4.76, ^3^*J* = 11.68 Hz, 1H, pyrazoline-H5), 6.99 (d, ^3^*J* = 8.40 Hz, 1H, Ar–H), 7.31–7.38 (m, 6H, Ar–H), 7.52 (t, ^3^*J* = 7.74 Hz, 2H, Ar–H), 7.64 (d, ^3^*J* = 8.00 Hz, 1H, Ar–H), 7.71 (d, ^3^*J* = 7.40 Hz, 1H, Ar–H), 7.92 (d, ^3^*J* = 8.00 Hz, 2H, Ar–H), 8.43 (s, 1H, pyrazole-H) ppm; ^13^C NMR (100 MHz; DMSO-*d*_6_) *δ*_C_ 21.51 (CH_3_), 42.32 (pyrazoline-C4), 52.29 (pyrazoline-C5), 56.01 (OCH_3_), 56.04 (OCH_3_), 104.57, 109.62, 111.75, 111.87, 118.89, 120.98, 121.79, 123.80, 124.30, 124.42, 125.21, 127.17, 127.23, 128.72, 130.00, 139.53, 140.83, 149.20, 150.00, 151.23, 154.54, 154.65, 168.01 (Ar–C), 172.53 (CO) ppm; MS, *m*/*z* (%): 506 [M˙^+^] (28.64), analysis for C_30_H_26_N_4_O_4_ (506.56), calcd% C, 71.13; H, 5.17; N, 11.06 found: % C, 71.26; H, 5.20; N, 10.94.

##### 2.1.4.4. 1-(5-(3-(Benzofuran-2-yl)-1-phenyl-1*H*-pyrazol-4-yl)-3-(1*H*-benzo[*d*]imidazol-2-yl)-4,5-dihydropyrazol-1-yl)ethanone (4d)

Yield (75%), mp 130–132 °C, IR (*ν*_max_/cm^−1^): 3394 (NH), 3062 (CH, aromatic), 2924, 2851 (CH-aliphatic), 1655 (C=O), 1597 (C=C); ^1^HNMR (400 MHz; DMSO-*d*_6_) *δ*_H_ 2.43 (s, 3H, CH_3_), 2.73–2.79 (dd, ^2^*J* = 8.00, ^3^*J* = 15.32 Hz, 1H, pyrazoline-H4), 2.86–2.96 (dd, ^2^*J* = 8.20, ^3^*J* = 14.52 Hz, 1H, pyrazoline-H4), 4.14–422 (dd, ^2^*J* = 12.16, ^3^*J* = 18.12 Hz, 1H, pyrazoline-H5), 6.98 (m, 2H, Ar–H), 7.15–7.41 (m, 2H, Ar–H), 7.46–7.58 (m, 6H, Ar–H), 7.65–7.74 (m, 4H, Ar–H), 8.25 (s, 1H, pyrazole-H), 12.20 (br.s, 1H, NH, D_2_O exchangeable) ppm; ^13^C NMR (100 MHz; DMSO-*d*_6_) *δ*_C_ 18.65 (CH_3_), 42.20 (pyrazoline-C4), 52.10 (pyrazoline-C5), 104.48, 105.04, 111.64, 114.73, 118.64, 118.79, 121.74, 121.77, 123.69, 125.24, 126.96, 128.76, 128.82, 130.05, 130.12, 139.55, 143.58, 149.74, 152.80, 154.23 (Ar–C), 168.10 (CO) ppm; MS, *m*/*z* (%): 486 [M˙^+^] (30.03), analysis for C_29_H_22_N_6_O_2_ (486.54), calcd% C, 71.59; H, 4.56; N, 17.27 found: % C, 71.62; H, 4.72; N, 17.30.

### 2.2. Biological assessment

#### 2.2.1. *In vitro* anticancer activity against 60 cell lines

##### 2.2.1.1. Preliminary screening at single high dose (10 μM)

All the synthesized compounds 2a–d, 3a–d and 4a–d, were selected by the National Cancer Institute (NCI), USA, for a single-dose screening assay against a panel of sixty human cell lines.^15,39–41^ The experimental procedure and resulting data are presented in the SI.

###### 2.2.1.2. Five-dose full NCI 60 cell panel assay

The compound 3d was further evaluated by the National Cancer Institute (NCI), USA, using a five-dose screening assay (0.01, 0.1, 1, 10, and 100 μM) against a panel of sixty human cell lines.^15,39–41^ The experimental methods and corresponding results are detailed in the SI.

The cytotoxicity assays were done at the National Cancer Institute (NCI), Bethesda, USA against 60 cell lines according to the protocol of the Drug Evaluation Branch, NCI.^15,39–41^

#### 2.2.2. *In vitro* multikinase inhibition assessment

Compound 3d, which exhibited the most potent antiproliferative activity, was further examined for its inhibitory activities against the c-MET, B-Raf, Pim-1, EGFR, and VEGFR-2 kinases. More details are presented in the SI.

#### 2.2.3. Cell cycle analysis

The MCF-7 cell line was treated with the most potent compound 3d at its GI_50_ concentration for 48 h. More details are presented in the SI.

#### 2.2.4. Apoptosis assay

The annexin V-FITC apoptosis detection kit (BD Biosciences) was used to quantify the percentage of cells undergoing apoptosis and detect the modes of cell death, either by apoptosis or necrosis, in the presence or absence of the active compound 3d. The experiment was carried out according to the manufacturer's protocol. More details are presented in the SI.

### 2.3. *In silico* study

AutoDock Vina 1.1.2 (ref. 69 and 70) was employed to perform the docking studies, using the human cMET protein structure (PDB 6SDE), EGFR (PDB 1XKK), VEGFR2 (PDB 4ASD), mutated B-Raf “V600E” (PDB 4XV2) and Pim-1 (PDB 1YHS) to evaluate the binding potential of the newly synthesized compound 3d in comparison to the reference drug for each enzyme. MGL Tools 1.5.7 was used to prepare and convert the protein and ligand files to pdbqt format. The results were visualized with Discovery Studio Visualizer v21.1.0.20298.

## 3. Results and discussion

### 3.1. Chemistry

**Scheme 1:**
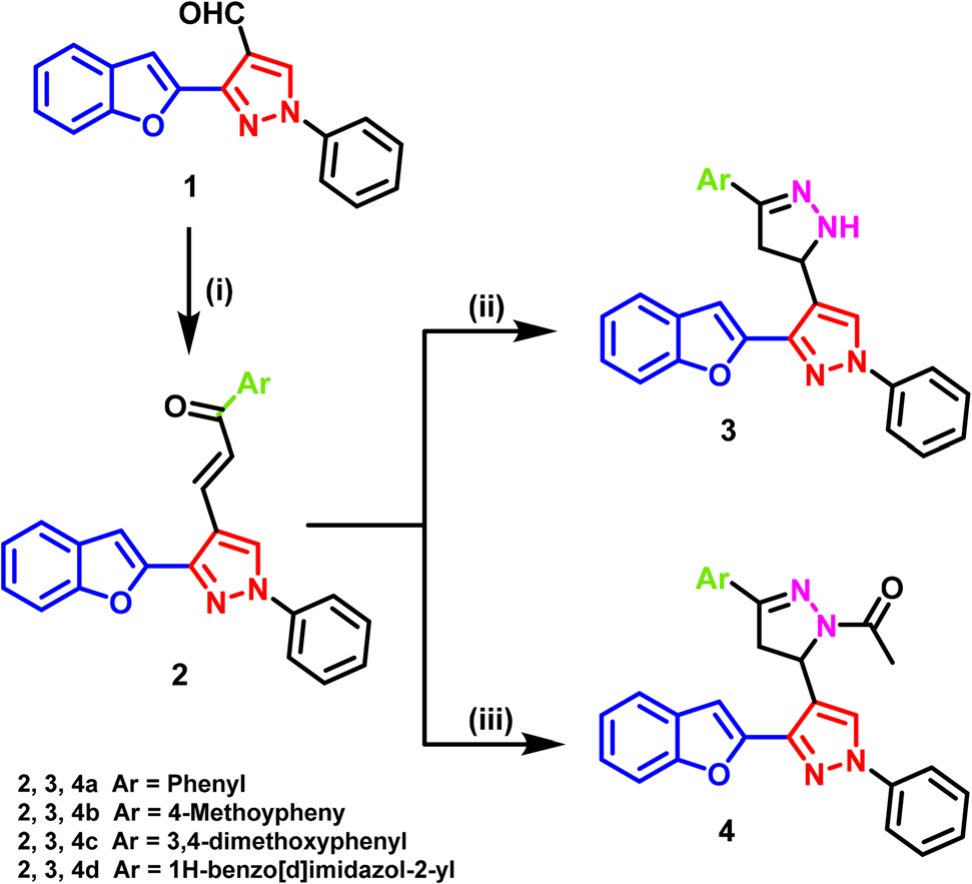
Synthesis of the target pyrazoline derivatives.

The synthetic methods used in this investigation to create the novel derivatives of the benzofuran–pyrazole hybrid are shown in Scheme 1. The crucial starting material 3-(benzofuran-2-yl)-1-phenyl-1*H*-pyrazole-4-carbaldehyde (1) was produced using the published Vilsmeier–Haack process.^71^ Briefly, to a cooled solution of phosphorus oxychloride in anhydrous DMF at 0 °C, the pyrazole precursor was added portion-wise under stirring. The reaction mixture was then allowed to warm to room temperature and stirred for 4–6 h. After completion, the mixture was poured onto crushed ice, neutralized with saturated sodium acetate solution, and stirred for 30 min. The resulting precipitate was filtered, washed with water, and recrystallized from ethanol to afford the target aldehyde 1 in 70–80% yield. The structure was confirmed by comparison with literature data. Chalcones have a history of being used as useful bridges in the synthesis of five- and six-membered heterocycles.^72^ Thus, the developed synthetic approach was designed to construct benzofuran–pyrazole-based dihydropyrazole derivatives efficiently and under relatively mild conditions. The cyclocondensation of chalcones 2a–d with hydrazine hydrate was performed in two solvent systems, absolute ethanol and glacial acetic acid, to direct the formation of two distinct series of products. Refluxing in absolute ethanol favored the formation of the 3-substituted dihydropyrazolyl pyrazole derivatives 3a–d. Ethanol was chosen because it dissolves both hydrazine and chalcones well, providing a homogeneous reaction medium that promotes smooth cyclization without significant byproduct formation. Its protic nature may also stabilize reaction intermediates *via* hydrogen bonding, supporting the selective formation of the pyrazoline ring. Conversely, cyclocondensation in glacial acetic acid led to the formation of the 1-substituted dihydropyrazolyl ethanone derivatives 4a–d. Acetic acid served both as a solvent and mild acid catalyst, activating the chalcone carbonyl group and directing regioselective ring closure. The acidic environment stabilizes protonated intermediates, favoring the observed selectivity toward the 1-substituted products. In comparison to alternative methods reported in the literature, the adopted conditions offered distinct advantages. For instance, cyclization under microwave irradiation achieves fast conversion but may risk partial decomposition of the labile groups or generate side products, requiring further purification.^73^ Similarly, cyclization in the presence of strong mineral acids (*e.g.*, concentrated HCl and H_2_SO_4_) can complicate isolation by promoting polymerization or overreaction.^74^ In contrast, our classical reflux protocol requires no specialized equipment, proceeds under atmospheric pressure, and provides high yields (around 75%) with simple filtration and recrystallization, highlighting its operational simplicity and selectivity.

The molecular structures of the synthesized derivatives were validated by elemental tests and spectral data. A pair of doublets at *δ* 6.31–7.10 and 7.60–8.10 ppm was visible in the ^1^H NMR spectra of the derived derivatives 2a–d. These doublets were caused by the *trans*-olefinic protons and had coupling constant values of *J* = 15.0–15.5 Hz. Besides the expected signals of the parent protons, the ^1^H NMR spectra of compounds 3a–d and 4a–d exhibited the two methylene protons of CH_2_ of the pyrazoline ring as a pair of doublets in the range of *δ* 2.91–3.11 and 3.41–3.65 ppm, while its methine proton CH presented as two doublets at the range of *δ* 5.19–5.26 ppm.

### 3.2. Biological assessment

#### 3.2.1. *In vitro* preliminary antitumor effects at a single dose (10 μM) against the full NCI 60 cell panel

The newly developed benzofuran–pyrazole derivatives 2–4 underwent preliminary anticancer screening at the National Cancer Institute (NCI), USA, as part of the screening project.

Following the NCI, USA protocol (https://dtp.nci.nih.gov), all the recently created compounds were assessed *in vitro* using a single dose (10 μM) against full NCI 60 cell line panels that implied nine distinct categories of cancer, including leukemia, non-small cell lung cancer, melanoma, CNS cancer, ovarian cancer, renal cancer, prostate cancer, and breast cancer.

The results are listed as percentage of growth inhibition (GI%) of the evaluated derivatives against the full panel of cell lines, ranging from 0 to 100% (Table 1). The COMPARE tool was used to analyze the single-dose evaluation findings of all the evaluated derivatives 2–4 against the sixty cancer cell lines.

**Table 1:** *In vitro* growth inhibition% (GI%) of the NCI 60 cancer cell line panel after treatment with 10 μM of the benzofuran–pyrazole hybrids 2–4

Cell name	GI%											
	2a	2b	2c	2d	3a	3b	3c	3d	4a	4b	4c	4d
**Leukemia**												
CCRF-CEM	—	19.37	8.09	—	12.98	17.25	84.09	L	15.54	10.80	9.93	10.91
HL-60(TB)	7.64	10.93	6.49	8.55	0.73	2.83	88.80	L	2.27	0.51	12.17	5.44
K-562	5.10	8.35	5.48	4.77	6.49	7.50	87.48	92.52	6.90	5.81	7.80	2.81
MOLT-4	3.22	7.84	4.38	3.61	0.33	8.04	80.97	88.75	7.01	7.68	8.51	5.60
RPMI-8226	—	10.27	4.63	0.67	2.58	7.55	88.22	L	6.01	4.36	11.35	4.74
SR	17.52	16.61	17.15	14.03	15.96	7.24	77.33	91.17	17.00	12.12	16.71	18.72

**Non-small lung carcinoma**												
A549/ATCC	4.22	—	—	—	3.63	—	57.50	87.82	—	—	2.60	—
EKVX	0.89	5.88	0.81	2.15	6.43	0.52	57.67	78.25	6.06	—	2.78	5.61
HOP-62	—	2.25	4.42	0.08	2.07	4.32	33.71	74.46	1.18	2.33	3.35	—
HOP-92	—	18.73	13.94	—	22.19	3.27	75.25	L	16.24	8.59	—	8.82
NCI-H226	—	0.47	1.83	—	8.13	1.48	68.39	34.35	0.69	—	1.68	3.03
NCI-H23	1.16	—	—	—	—	—	53.25	78.78	—	—	3.23	—
NCI-H322M	—	2.02	—	—	1.85	2.93	41.54	85.31	2.33	3.11	2.32	2.19
NCI-H460	—	—	—	—	—	—	81.94	97.07	—	—	—	—
NCI-H522	3.81	0.40	1.92	0.40	3.69	0.82	51.41	96.77	2.75	1.65	1.54	1.70

**Colon cancer**												
COLO 205	—	—	—	—	91.61	—	47.04	—	—	—	—	—
HCC-2998	—	—	—	—	66.78	—	64.60	70.76	—	—	—	—
HCT-116	—	—	0.17	—	99.83	—	74.37	76.76	—	—	—	—
HCT-15	—	—	1.66	—	75.31	1.12	49.94	83.02	2.61	0.50	2.45	1.38
HT29	—	—	—	—	54.87	—	64.31	64.97	0.65	—	—	0.62
KM12	1.62	—	—	—	62.60	—	68.12	91.86	—	—	1.20	—
SW-620	0.70	—	—	—	79.60	—	47.04	76.92	—	—	4.67	—

**CNS cancer**												
SF-268	4.98	—	1.66	—	—	—	61.15	72.21	—	—	4.67	1.38
SF-295	5.84	7.09	9.48	4.39	9.51	1.43	58.47	84.73	5.69	—	4.33	9.71
SF-539	0.23	3.20	4.21	1.65	6.00	4.09	56.77	85.47	8.41	2.46	1.95	0.68
SNB-19	4.72	6.26	4.96	4.33	5.98	2.46	59.83	92.86	5.00	2.45	6.15	4.75
SNB-75	19.77	—	—	20.70	−10.52	0.28	58.00	65.51	1.45	—	14.70	—
U251	—	—	—	—	2.33	—	60.18	85.69	1.60	—	—	—

**Melanoma**												
LOX IMVIL	—	4.44	—	—	—	—	65.24	75.62	4.25	2.30	3.27	—
MALME-3M	1.12	1.29	5.86	0.07	3.67	4.88	30.46	81.85	1.02	0.92	3.54	5.76
M14	0.52	—	1.79	—	0.57	4.94	63.24	83.05	—	—	—	2.74
MDA-MB-435	0.51	—	—	—	−6.37	—	65.91	83.49	—	—	—	—
SK-MEL-2	—	—	—	—	−6.14	—	47.50	99.44	—	—	1.30	—
SK-MEL-28	—	—	—	—	2.00	—	35.60	80.99	—	—	—	1.16
SK-MEL-5	—	—	1.65	—	0.18	—	74.73	90.42	0.15	—	5.22	0.87
UACC-257	5.50	—	—	1.66	6.06	—	45.64	79.15	—	—	6.12	1.22
UACC-62	2.28	7.77	4.68	—	7.34	8.16	62.47	L	8.33	5.56	—	2.82

**Ovarian carcinoma**												
IGROV1	—	—	—	—	—	—	61.79	89.61	—	—	—	—
OVCAR-3	—	—	—	—	—	—	65.22	81.63	—	—	—	—
OVCAR-4	1.64	—	—	—	—	—	58.72	66.52	—	—	—	—
OVCAR-5	0.37	2.97	0.00	—	3.74	—	41.14	66.23	3.55	—	5.34	6.06
OVCAR-8	—	—	—	—	—	1.00	63.70	86.94	—	—	—	—
NCI/ADR-RES	—	—	1.79	—	—	—	—	—	—	—	—	—
SK-OV-3	—	4.75	11.15	4.37	8.93	6.45	32.78	62.21	9.60	4.82	7.21	—

**Renal carcinoma**												
4.42786–0	1.14	1.04	2.44	0.74	—	—	52.36	71.73	2.59	—	4.42	4.06
A498	1.16	—	6.80	—	6.22	2.47	7.63	54.33	—	—	3.05	5.12
ACHN	—	0.20	0.70	—	—	5.79	74.84	92.45	—	—	—	—
CAKI-1	15.01	12.51	10.30	10.24	11.52	17.83	76.85	92.45	13.36	13.20	13.64	7.51
RXF 393	—	—	—	—	—	—	36.89	63.52	—	—	—	2.67
SN12C	0.82	—	10.90	3.48	12.27	—	63.01	88.17	—	—	10.86	6.54
TK-10	5.20	6.22	6.22	3.41	10.65	3.17	42.27	75.14	11.62	7.73	—	15.06
UO-31	7.39	14.83	15.32	7.86	14.55	26.14	80.36	L	15.50	17.79	11.00	13.92

**Prostate carcinoma**												
PC-3	—	4.83	7.06	—	8.08	12.08	66.98	91.14	8.29	6.35	3.89	5.99
DU-145	—	—	—	—	—	—	58.05	67.57	—	—	1.59	—

**Breast carcinoma**												
-MCF7	5.38	0.75	9.78	5.90	7.06	2.03	77.82	85.98	11.19	9.85	8.83	5.40
BT-549	—	69.00	16.27	15.03	0.80	4.37	—	—	9.06	—	3.97	9.21
T-47D	—	83.54	9.23	7.67	10.71	2.60	81.51	88.48	12.41	2.42	5.68	18.84
MDA-MB-468	0.19	65.44	0.93	1.87	—	7.03	78.06	70.95	3.32	2.43	8.49	—
Mean GI%	0.37	0.61	2.16	−0.94	2.24	0.8	60.71	83.58	1.66	−2.13	2.51	1.26

Except for 3c and 3d, which showed superior activity, the GI values of the examined compounds showed moderate to weak anticancer activity. Compounds 3c and 3d demonstrated promising broad-spectrum cytotoxic activity against several of the tested cancer cell lines.

According to the obtained data (Table 1), compound 3d exhibited a pronounced selective GI% against 2 subpanels of leukemia cancer cell lines, namely, K-562 and SR (GI% = 92.52%, and 91.17%, respectively); 2 subpanels of non-small cell lung carcinoma (NSCLC), NCI-H460 and NCI-H522 (GI% = 97.07% and 96.77%); KM12 subpanel of colon cancer (GI% = 91.86%); SNB-19 subpanel of CNS cancer (GI% = 92.86%); 2 subpanels of melanoma cancer cell lines, SK-MEL-2 and SK-MEL-5 (GI% = 99.44% and 90.42%); and 2 subpanels of renal carcinoma lines, ACHN and CAKI-1 (GI% = 92.45% and 92.45%), respectively.

The obtained results demonstrated the influence of the type of attached ring system to the linker bridge on the anticancer activity. The presence of the 1*H*-benzo[*d*]imidazol-2-yl ring in 3d produced the most potent anticancer activity against various subpanels of examined cancer cell lines, which is expected due to the extra hydrophobic interactions with different proteins, resulting in a better fit in the active sites of the enzymes 3d acts on. Additionally, the unsubstituted NH group of the attached pyrazoline ring acts as a centre for hydrogen-bonding interactions.

The benzofuran–pyrazole-benzo[*d*]imidazole derivative 3d was selected for further detailed study at five different concentrations due to its significant data in the single-dose study.

#### 3.2.2. *In vitro* anticancer assessment at 5 doses on full NCI 60 cell panel

In the second step, the chosen candidate 3d (D-804233/1), which met established threshold inhibition standards, was tested against all 60 cell lines at tenfold dilutions of five distinct concentrations (0.01, 0.1, 1, 10, and 100 μM).^75,76^

Table 2 displays the calculated response parameters, GI_50_ and LC_50_, against the assessed cell lines. GI_50_ denotes the compound concentration that results in a 50% suppression in net cell growth, and LC_50_ for the lethal dose, which denotes the compound concentration that results in 50% loss of the initial cells.^77^ Additionally, the subpanel and full panel mean graph midpoints (MG-MID) were computed for the GI_50_ values to represent the average activity parameter over the subpanels and full panel cell lines for 3d (Table 3). The five-dose anticancer screening showed that 3d had significant effects on the examined cancer cell lines (39 of them had GI_50_ values less than 4.00 μM, and the values ranged from 1.84 to 6.62 μM). Luckily, the LC_50_ values of 3d exceeded 100 μM against the majority of examined cell lines with a few exceptions, indicating that it had non-lethal effects (Table 3).

**Table 2:** Anti-proliferative activity of compound 3d on NCI cancer cell lines at 5 dose levels

Leukemia	GI_50_ (μM)	Colon cancer	GI_50_ (μM)	Melanoma	GI_50_ (μM)	Renal cancer	GI_50_ (μM)
CCRF-CEM	3.11	COLO 205	5.28	LOX IMVI	3.71	786–0	4.31
HL-60(TB)	2.56	HCC-2998	5.25	MALME-3M	6.19	A498	12.70
K-562	3.37	HCT-116	5.46	M14	3.22	ACHN	3.11
MOLT-4	4.13	HCT-15	3.18	MDA-MB-435	4.24	CAKI-1	2.57
RPMI-8226	3.04	HT29	6.46	SK-MEL-2	2.53	RXF 393	6.25
SR	3.15	KM12	5.55	SK-MEL-28	4.58	SN12C	3.42
Non-small cell lung cancer		SW-620	5.95	SK-MEL-5	2.76	TK-10	4.97
A549/ATCC	3.86	CNS cancer		UACC-257	6.24	UO-31	2.10
EKVX	2.69	SF-268	4.51	UACC-62	1.84	Prostate cancer	
HOP-62	3.66	SF-295	2.59	Ovarian cancer		PC-3	3.47
HOP-92	4.78	SF-539	3.97	IGROV1	3.34	DU-145	5.15
NCI-H226	6.62	SNB-19	3.77	OVCAR-3	4.91	Breast cancer	
NCI-H23	3.69	SNB-75	2.72	OVCAR-4	3.51	MCF7	2.75
NCI-H322M	3.77	U251	3.72	OVCAR-5	5.52	MDA-MB-231/ATCC	4.78
NCI-H460	3.64			OVCAR-8	5.88	HS 578T	7.94
NCI-H522	3.48			NCI/ADR-RES	3.16	BT-549	3.85
						T-47D	3.12

**Table 3:** Mean graph midpoint values (MG-MIDa) for the subpanel tumor cell line GI_50_ parameter (μM)^a^

Subpanel type	MG-MID	Selectivity index
Leukemia	3.23	1.30
NSCL cancer	4.02	1.04
Colon cancer	5.02	0.83
CNS cancer	3.55	1.18
Melanoma	3.92	1.07
Ovarian cancer	4.32	0.97
Renal cancer	4.93	0.85
Prostate cancer	4.31	0.97
Breast cancer	4.49	0.935

a Full panel MG-MID for 3d = 4.20 μM.

The selectivity index (SI) was computed by dividing the full panel MG-MID (μM) for the tested compound by its subpanel MG-MID (μM). SI is a measuring factor for compound selectivity towards subpanels. Compound 3d showed non-selective, broad-spectrum anticancer activity against all the cancer subpanels, with selectivity ratios ranging from 0.83 to 1.3 (Table 3).

#### 3.2.3. *In vitro* multikinase inhibition assessment

Based on the obtained anticancer evaluation, the most potent compound, 3d, was further assessed for its *in vitro* multi-targeting PK-inhibiting impact against B-Raf (V600E), c-Met, Pim-1, EGFR (WT), and VEGFR-2. The most common PKI drugs, vemurafenib, erlotinib, staurosporine, and sorafenib, cause intracellular phosphorylation-inhibiting effects against the examined protein kinases, serving as positive controls^78,79^ (Table 4). Compound 3d was 1.2- and 4.1-times more effective at suppressing EGFR (WT) and VEGFR-2 (IC_50_ = 0.177 ± 0.007 and 0.275 ± 0.011 μg mL^−1^) than the reference drugs erlotinib and sorafenib (IC_50_ = 0.220 ± 0.15 and 1.12 ± 0.10 μg mL^−1^), respectively. Also, it had the same effect on c-Met as staurosporine, with IC_50_ values of 0.405 ± 0.017 μg mL^−1^. Alternatively, the kinases B-Raf (V600E) and Pim-1 were 2.8- and 4.9-times less sensitive to the tested compound (IC_50_ = 0.078 ± 0.004 and 1.053 ± 0.046 μg mL^−1^) than the reference standards vemurafenib and staurosporine (IC_50_ values of 0.027 ± 0.001 and 0.213 ± 0.009 μg mL^−1^), respectively. The resultant data showed a favorable correlation with the corresponding values for their antiproliferative activities.

**Table 4:** Multi-targeting suppression of compound 3d against different protein kinases measured in μg mL^−1^

Compound name	B-Raf (V600E)	c-Met	Pim-1	EGFR (WT)	VEGFR-2
3d	0.078 ± 0.004	0.405 ± 0.017	1.053 ± 0.046	0.177 ± 0.007	0.275 ± 0.011
Vemurafenib	0.027 ± 0.001				
Erlotinib				0.220 ± 0.15	
Staurosporine		0.40 ± 0.014	0.213 ± 0.009		
Sorafenib					1.12 ± 0.10

The promising multikinase suppression activity was confirmed by a docking study, underscoring the significance of the NH group of the benzo[*d*]imidazole ring, which facilitates interactions of 3d through hydrogen bonds with various amino acid residues, including ARG1086, GLU885, LYS483, and ASP594 of the evaluated kinases, alongside the hydrophobic, pi–cation, and pi–sulphur interactions of the other fragments of the molecule with various amino acid residues.

#### 3.2.4. Cell cycle distribution and apoptosis detection

##### 3.2.4.1. Cell cycle assay

Normal cell growth and division are governed by four cell cycle stages of pre-G1, G1, S, and G2/M phases. However, the majority of cancer cells frequently undergo uncontrollable cell divisions brought on by cell cycle down-regulation. Therefore, targeting particular cell cycle stages is a crucial therapeutic approach in the management of antiproliferative disorders.^80^

Given that 3d has emerged as a well-balanced active compound that functions as both an anticancer agent and a multi-targeting protein kinase suppressor, it was of interest to gain a deeper understanding of how 3d inhibits the growth of cancer cells. A propidium iodide (PI) staining experiment was performed in this study to look at its effect on cell cycle distribution and apoptosis activation.^81,82^

**Fig. 3:**
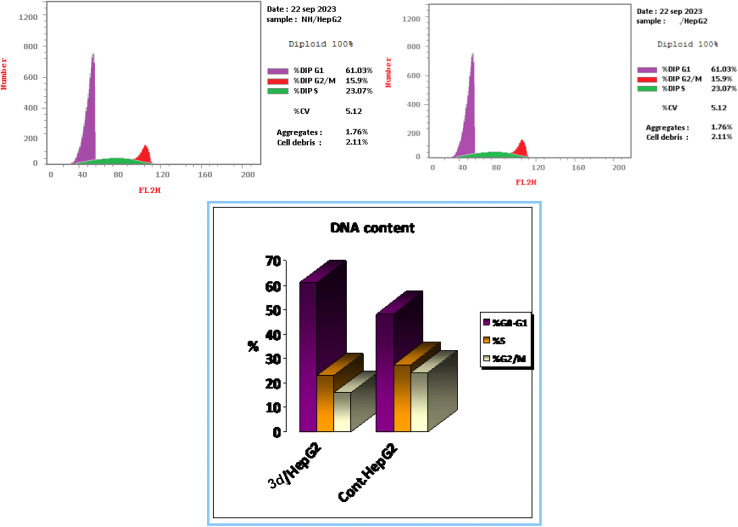
Cell cycle detection of compound 3d on the HepG-2 cancer cell line in comparison with the untreated cells.

When 3d was added to MCF-7 cancer cells at its GI_50_ concentration of 2.75 μM, the percentage of cells in the G0–G1 phase increased compared to cells that had not been treated (61.03% *vs.* 48.39%). Conversely, the cell percentage in the S phase was reduced from 27.51% in the control cells to 23.077% in the 3d-treated cells and in the G2/M stage, from 24.1% in the control cells to 15.9% in the 3d-treated cells. This blocked cell cycle passage at the G0–G1 stage can stop cells from dividing and growing, which is in line with that shown by other tests about the ability of compound 3d to stop cell division and growth (Fig. 3).

##### 3.2.4.2. Apoptosis detection

**Fig. 4:**
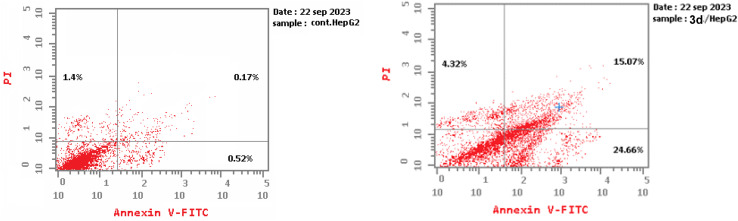
Apoptosis detection of compound 3d on the HepG-2 cancer cell line in comparison with the untreated cell line.

Flow cytometry assay employing annexin V-FITC and propidium iodide double labeling was used to further examine the apoptotic impact of compound 3d.^81,82^ Exposure of HepG-2 cancer cells to 3d for 24 h resulted in early apoptosis with a value of 24.66% in HepG-2 cells, compared to 0.52% in the untreated control cells. Additionally, 3d enhanced the late apoptotic effect from 0.17% in the control cells to 15.07% in the treated cells. Moreover, compound 3d promoted necrosis by 4.32% in the 3d-treated cells in comparison with 1.4% in the untreated ones (Table 5 and Fig. 4). These findings support the cytotoxic action of compound 3d on HepG-2 cells. In addition, it implies that its cytotoxic impact is not only due to it initiating programmed cell death pathways in an orderly manner but also its ability to effectively complete the apoptotic process, which results in the fragmentation and removal of cancer cells.

**Table 5:** Apoptosis detection assay for compound 3d in HepG-2 cancer cells

	Total	Early	Late	Necrosis
NH/HepG-2	44.05	24.66	15.07	4.32
Cont.HepG-2	2.09	0.52	0.17	1.4

It has been reported that suppression activity against VEGFR-2 and EGFR results in the induction of cell death *via* both the apoptosis and necrosis pathways,^82^ which explains the necrotic effect of 3d against the examined cancer cells.

### 3.3. *In silico* investigation

#### 3.3.1. Molecular docking of compound 3d

**Fig. 5:**
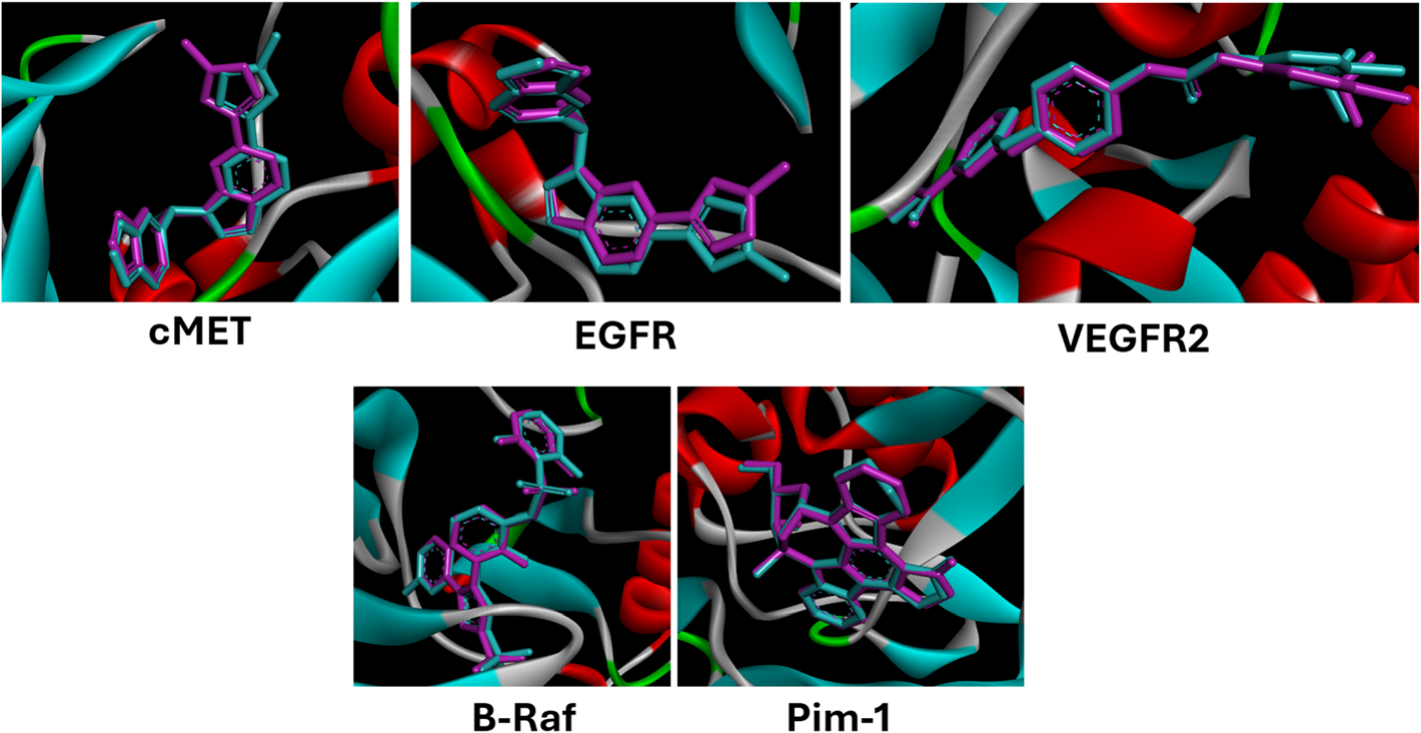
Validation of the docking procedure to c-MET, EGFR, VEGFR-2, B-Raf, and Pim-1, where the co-crystallized ligand is shown in cyan and the re-docked ligand in purple.

Firstly, we carried out a validation step to confirm the suitability of the docking protocol against the five target enzymes using AutoDock Vina 1.1.2.^69,70^ We calculated the root mean square deviation (RMSD) and found it within the accepted range of 1.08 for c-MET, 1.24 for EGFR, 1.01 for VEGFR-2, 0.34 for B-Raf, and 0.23 for Pim-1 (ref. 83) (Fig. 5).

**Fig. 6:**
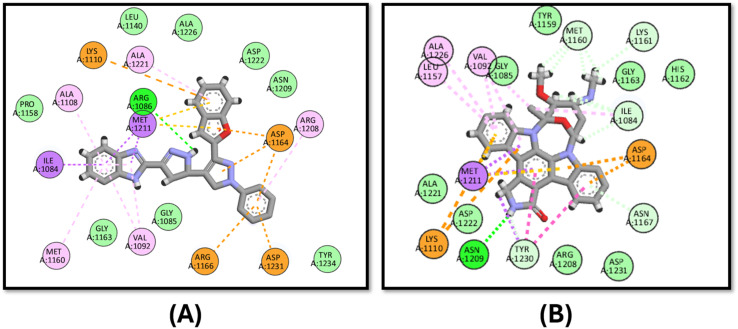
Docking poses of (A) compound 3d and (B) staurosporine inside the active site of c-MET kinase.

The tested compound 3d showed comparable activity to that of staurosporine against c-MET kinase with IC_50_ values of 0.405 ± 0.017 and 0.40 ± 0.014 μg mL^−1^, respectively, and the docking study proved this finding given that it has a docking binding energy score of −9.0 kcal mol^−1^ compared to that of the staurosporine “−10.6 kcal mol^−1^”. Compound 3d binds to ARG1086 through hydrogen bonding in addition to pi–cation interactions with ARG1166, LYS1110 and pi–anion forces to ASP1231 (Fig. 6).

**Fig. 7:**
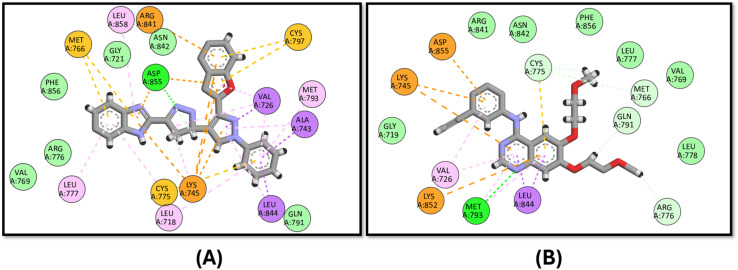
Docking poses of (A) compound 3d and (B) lapatinib inside the active site of EGFR.

The binding affinity of 3d was tested against EGFR in comparison to the co-crystallized ligand lapatinib. It has good affinity for the EGFR active site in comparison to lapatinib with binding energy scores of −9.2 and −11.4 kcal mol^−1^, respectively. It also binds to the key amino acid MET793 through hydrophobic interactions, while lapatinib binds to it *via* hydrogen bonding (Fig. 7).

**Fig. 8:**
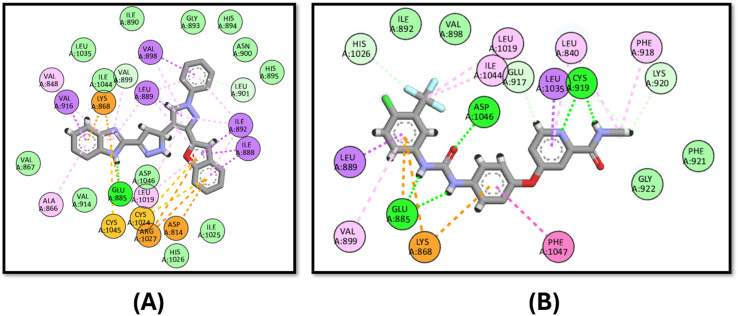
Docking poses of (A) compound 3d and (B) sorafenib inside the active site of VEGFR-2.

In the case of VEGFR-2, compound 3d has a comparable binding mode to that of the co-crystallized ligand, sorafenib (Fig. 8). 3d has an energy score of −10.1 kcal mol^−1^, while that of sorafenib is −10.2 kcal mol^−1^. Fig. 8 shows that both compounds bind to the key amino acid GLU885 through hydrogen binding and LYS868 through pi–cation interactions.

**Fig. 9:**
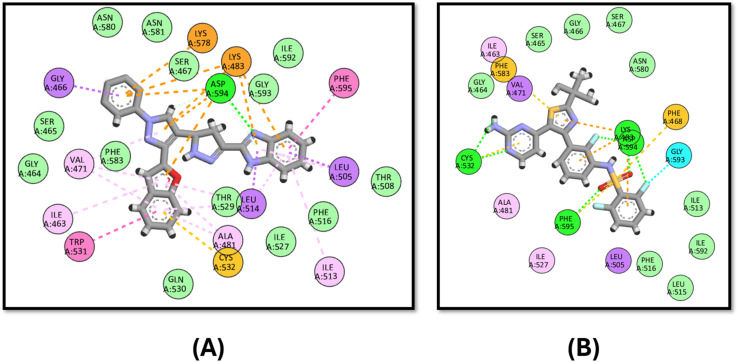
Docking poses of (A) compound 3d and (B) vemurafenib inside the active site of B-Raf “V600E”.

Moreover, 3d exhibits good binding affinity to the mutated B-Raf “V600E” with a score of −9.6 kcal mol^−1^ compared to the reference drug vemurafenib with a value of −10.5 kcal mol^−1^. It has a similar binding mode to that of vemurafenib through hydrogen bonding with LYS483 and ASP594 in addition to pi–sulfur interactions with CYS532 and PHE595, which endows it with good activity against the mutated B-Raf “V600E”, as shown in Fig. 9.

**Fig. 10:**
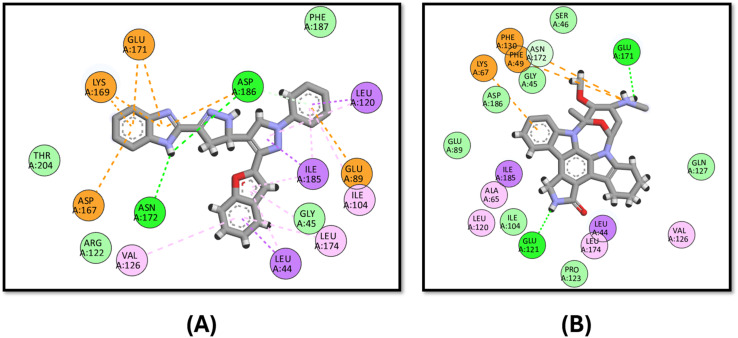
Docking poses of (A) compound 3d and (B) staurosporine inside the active site of Pim-1.

Finally, the docking of 3d against Pim-1 did not show any hydrogen bonding achieved by the reference staurosporine, instead, it binds to the ASN172 and ASP186 residues (Fig. 10).

In analyzing the binding interactions of compound 3d across various kinase targets, it is crucial to consider both the conserved and divergent structural features within the ATP-binding pocket and allosteric sites of these kinases. Despite sharing a common ATP-binding region, kinases such as B-Raf (V600E), c-Met, Pim-1, EGFR, and VEGFR-2 exhibit distinct structural nuances that govern the binding affinity and selectivity. For instance, B-Raf (V600E) harbors a mutated valine-to-glutamic acid residue at position 600, resulting in a hydrophobic pocket alteration that accommodates inhibitors such as vemurafenib more effectively.^84^ In contrast, Pim-1 features a less hydrophobic allosteric pocket, explaining the reduced binding efficacy of 3d, as observed in our study.^85^

Similarly, the EGFR and VEGFR-2 kinases share a relatively conserved hinge region that facilitates hydrogen bonding interactions, as evidenced by the binding of 3d to MET793 in EGFR and GLU885 in VEGFR-2. However, subtle variations in the surrounding residues, such as the bulky LYS868 in VEGFR-2, allow differential interactions, contributing to the distinct binding profiles observed.^86,87^ c-Met, characterized by a more polar active site due to residues such as ARG1086 and ASP1231, presents a mixed hydrophobic-polar environment that enables pi–cation and pi–anion interactions, respectively, with the tested compound.^88^

Thus, the observed multi-kinase inhibition profile of 3d can be rationalized by its structural adaptability to accommodate diverse kinase pockets, facilitated by its benzofuran–pyrazole scaffold and strategically positioned aromatic and hydrogen-bond donor/acceptor functionalities. This structural versatility underscores the potential of 3d as a multi-target kinase inhibitor capable of modulating different signalling pathways through differential binding modes. Further optimization to enhance the selectivity and minimize off-target interactions can be considered in future studies to further refine its kinase-targeting profile.

#### 3.3.2. ADME study of compound 3d

**Fig. 11:**
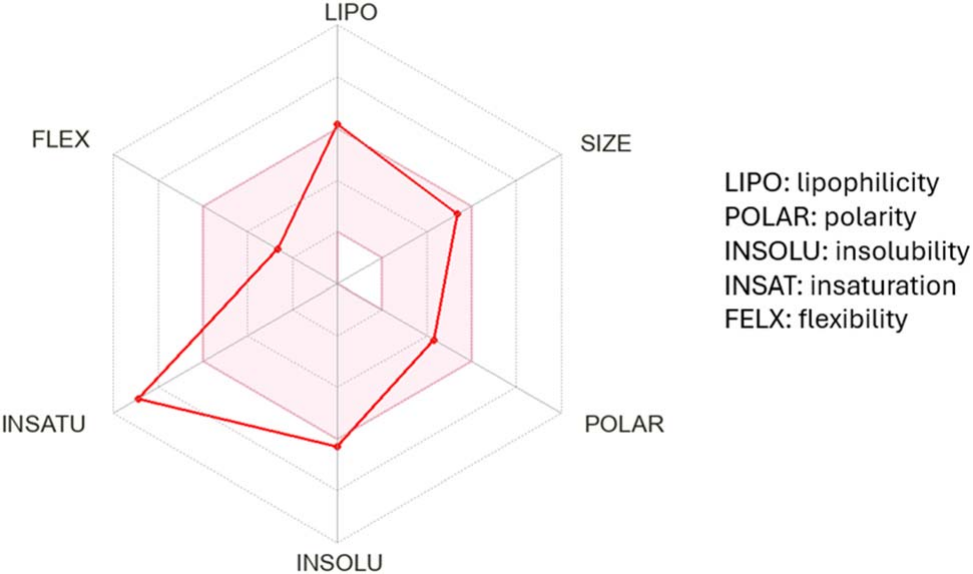
Bioavailability radar chart of compound 3d (the pink area represents the accepted range for each of the studied parameters).

The ADME study of compound 3d was carried out using the free SwissADME server.^89^ The bioavailability radar represents six key parameters relevant to drug-likeness (Fig. 11). 3d has two violations represented by the points that go outside the accepted pink area, which are unsaturation because this compound has a very low fraction of sp^3^ carbons and insolubility.

Lipinski's rule of five^90^ is also applied by measuring the molecular weight, the log *P* value, the number of hydrogen bond acceptor groups (O/N) and the number of hydrogen bond donor groups (OH/NH). Compound 3d has no violations given that its molecular weight is 444.49 g mol^−1^ (<500), its log value is 4.32 (<5), its number of hydrogen bond acceptor groups is 7 (<10), and its number of hydrogen bond donor groups is 2 (<5). These findings indicated the expected good oral bioavailability of 3d.

**Fig. 12:**
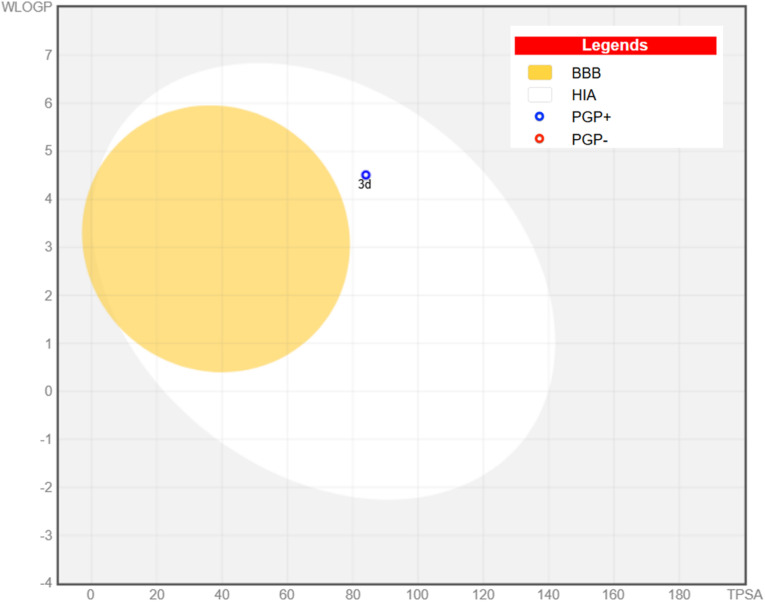
Boiled egg chart for compound 3d.

Finally, to assess the expected GIT absorption, BBB penetration and the possibility of being a substrate for the efflux protein permeability glycoprotein (P-gp),^91^ a boiled-egg chart was constructed (Fig. 12). Compound 3d showed good human intestinal absorption (HIA), as represented by the white area in the chart, but could not penetrate the BBB, which is represented by the yellow area. Unfortunately, 3d is expected to be a substrate for the efflux protein P-gp, which is indicated by the blue circle.

## 4. Conclusion

The goal of this study is to design and develop new benzofuran–pyrazole-based analogues, tethered with various substituted aromatic and heterocyclic ring systems featuring the pharmacophoric fragments of protein kinase suppressors 3a–d and 4a–d. The conclusions can be summarized according to the following points:

• All the new analogues were selected by the NCI to screen their antiproliferative activity against sixty human cancer cell lines (NCI60).

• The 1*H*-benzo[*d*]imidazole derivative 3d exhibited prominent % inhibition of various cancer cell lines and advanced to the five-dose assay. Luckily, it showed remarkable anti-proliferative activity against various types of cancer lines with GI_50_ values ranging from 0.33 to 4.87 μM.

• Fortunately, its LC_50_ exceeded 100 μM against the majority of examined cell lines, confirming its non-lethal effects.

• Due to its promising antiproliferative activity, compound 3d was further assessed for its *in vitro* multi-targeting PK-inhibiting activity against B-Raf (V600E), c-Met, Pim-1, EGFR (WT), and VEGFR-2. The most common PKI drugs, vemurafenib, erlotinib, staurosporine, and sorafenib, were chosen as standard controls.

• 3d exhibited a significant suppression effect against EGFR (WT) VEGFR-2, and c-Met (IC_50_ = 0.177 ± 0.007, 0.275 ± 0.011, and 0.405 ± 0.017, respectively). The kinases B-Raf (V600E) and Pim-1 were less sensitive to 3d (IC_50_ = 0.078 ± 0.004 and 1.053 ± 0.046 μg mL^−1^) than the reference standards vemurafenib and staurosporine (IC_50_ = 0.027 ± 0.001 and 0.213 ± 0.009 μg mL^−1^), respectively.

• Additionally, 3d resulted in early and late apoptosis in MCF-7 cancer cells and arrested the cell cycle at the G0–G1 phase.

• *In silico* molecular docking of 3d in the active sites of the tested kinases showed good affinity and good binding interactions with amino acid residues of c-MET, EGFR, VEGFR-2, and B-Raf “V600E”, with binding energy scores ranging from −9.0 to 10.5 kcal mol^−1^.

• In addition, the ADME study exhibited that compound 3d follows Lipinski's rule of five, indicating its expected good oral bioavailability. Also, compound 3d has good human intestinal absorption but cannot penetrate the BBB.

• Future prospects: more studies will be carried out for further optimization of the pharmacophoric structure of the benzofuran–pyrazole–pyrazoline parent scaffold, considering the significance of the unsubstituted NH groups of the pyrazoline and the benzo[*d*]imidazole ring, which facilitate hydrogen bond interactions with various amino acid residues of the evaluated kinases, including ARG1086, GLU885, LYS483, and ASP594, alongside the hydrophobic, pi–cation, and pi–sulphur interactions of the other fragments of the molecules with various amino acid residues. In addition, the peripheral benzimidazole has a polar imidazole ring (containing two nitrogen atoms) and a benzene ring, making it less hydrophobic overall, which directs the development of new molecules with a peripheral phenyl ring with polar and less hydrophobic substitutions, leading to more favorable docking scores and promising experimental cytotoxic and multi-kinase inhibition activity.

Also, *in vivo* and histopathological studies should be carried out in further studies to fully evaluate its safety and therapeutic efficacy. Thus, these results can provide a solid foundation for future drug discovery initiatives and contribute to the creation of new benzofuran–pyrazole-based anticancer drugs with multitargeting enzyme activities.

## Conflicts of interest

The authors declare that they have no known competing financial interests or personal relationships that could have appeared to influence the work reported in this manuscript.

## Supplementary Material

RA-015-D5RA00553A-s001

## Data Availability

Further inquiries can be directed to the corresponding author. The original contributions presented in the study are included in the article/SI. See DOI: https://doi.org/10.1039/d5ra00553a.
