# Supplementary material for: Design, synthesis, and in silico studies of new benzofuran–pyrazole hybrids as multi-kinase inhibitors with potential antiproliferative activity
Source: RSC Adv. 2025 Oct 1;15(42):35003–21. doi: 10.1039/d5ra00553a (PMC12486440; doi:10.1039/d5ra00553a)
Supplement: RA-015-D5RA00553A-s001 [file RA-015-D5RA00553A-s001.pdf]

Supplementary Information (SI) for RSC Advances.
This journal is © The Royal Society of Chemistry 2025

Supporting Information

Design, synthesis and *in silico* studies of new benzofuran-pyrazole hybrids as multi-kinase inhibitors of potential antiproliferative activity

Somaia S. Abd El-Karim[1*], Yasmin M. Syam[1], Reham M. Abdelkader[2], Mohamed K. El-Ashrey, Manal M. Anwar[1*],

[1]Department of Therapeutic Chemistry, National Research Centre, El-Bohouth St., 12262, Cairo, Egypt
[2] Department of pharmacology and toxicology, faculty of pharmacy and biotechnology, German university in Cairo, Cairo, Egypt
[3] Pharmaceutical Chemistry Department, Faculty of Pharmacy, Cairo University, Kasr Elini St., Cairo 11562, Egypt.
[4] Medicinal Chemistry Department, Faculty of Pharmacy, King Salman International University (KSIU), South Sinai 46612, Egypt.

[*]Corresponding author, Email address: ssabdelkarim@gmail.com, manal.hasan52@live.com

Experimental protocols

5.1. Chemistry

All melting points are uncorrected and were taken in open capillary tubes using Electrothermal apparatus 9100. Elemental microanalyses were carried out at Microanalytical Unit, Central Services Laboratory, National Research Centre, Dokki, Cairo, Egypt, using Vario Elementar and were found within ±0.4% of the theoretical values. Infrared spectra were recorded on a FT/IR-4100 Jasco-Japan, Fourier transform, Infrared spectrometer at cm-1 scale using KBr disc technique at Central Services Laboratory, National Research Centre, Dokki, Cairo, Egypt.
^{1}H NMR and ^{13}C NMR spectra were determined by using a Bruker High Performance Digital FT-NMR Spectrometer Avance III 400MHz, Faculty of Pharmacy-Cairo University,Cairo, Egypt. Chemical shifts were expressed in δ (ppm) downfield from TMS as an internal standard. chemical shifts are expressed in δ (ppm) downfield from TMS as an internal standard. The mass spectra were measured with a GC MS-Qp1000EX Shimadzu, Cairo University, Cairo, Egypt. Follow up of the reactions and checking the purity of the compounds were made by TLC on silica gel-precoated aluminium sheets (Type 60, F 254, Merck, Darmstadt, Germany) using chloroform/methanol (20:1, v/v) and the spots were detected by exposure to UV lamp at λ254 nanometer for few seconds and by iodine vapor. The chemical names given for the prepared compounds are according to the IUPAC system.

3-(5-(Benzofuran-2-yl)-1-phenyl-1H-pyrazol-3-yl)-1-phenylprop-2-en-1-one (2a)

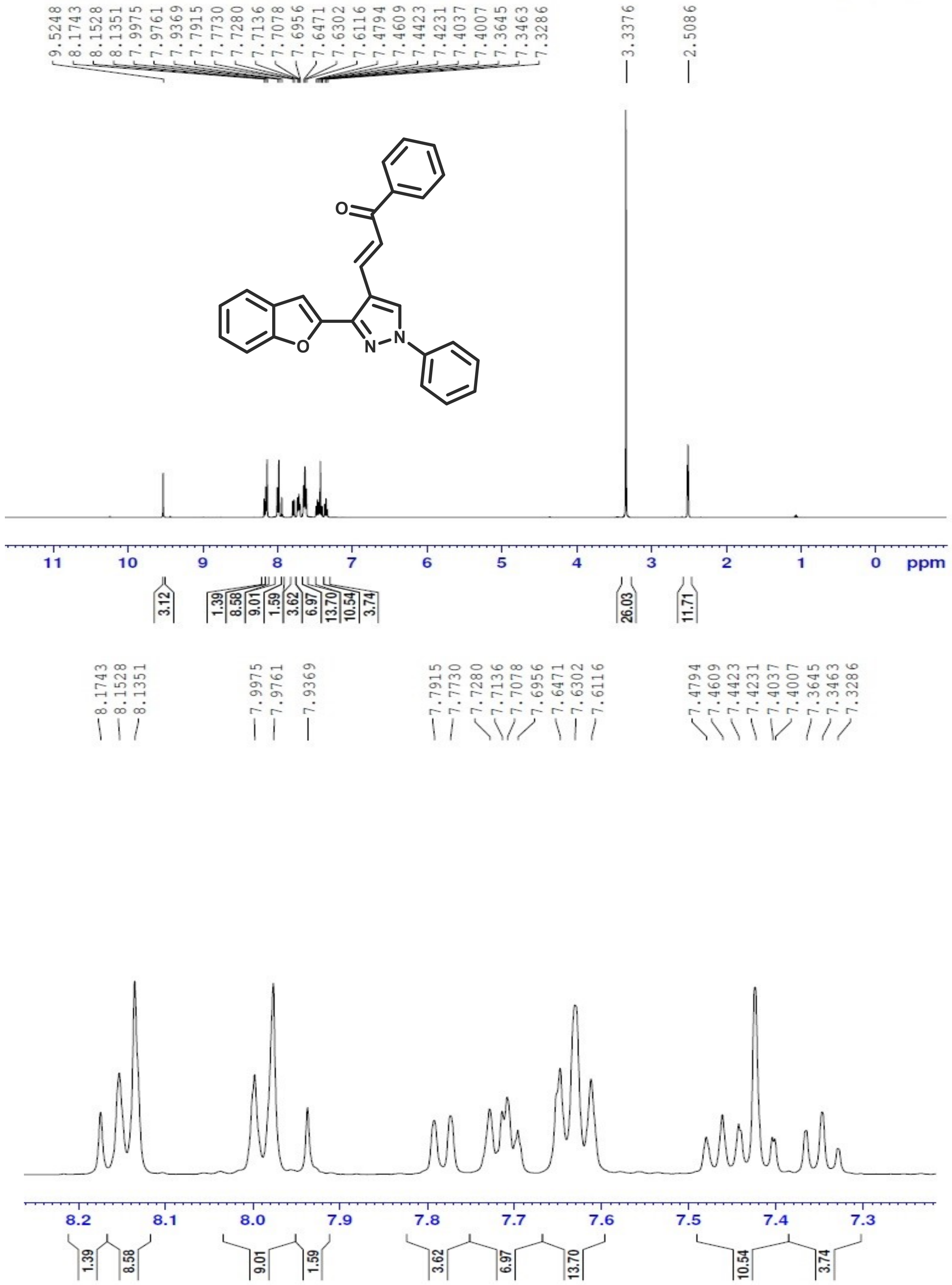

Figure S1: ^{1}HNMR (400 MHz; DMSO-*d6*) spectrum of compound **2a**

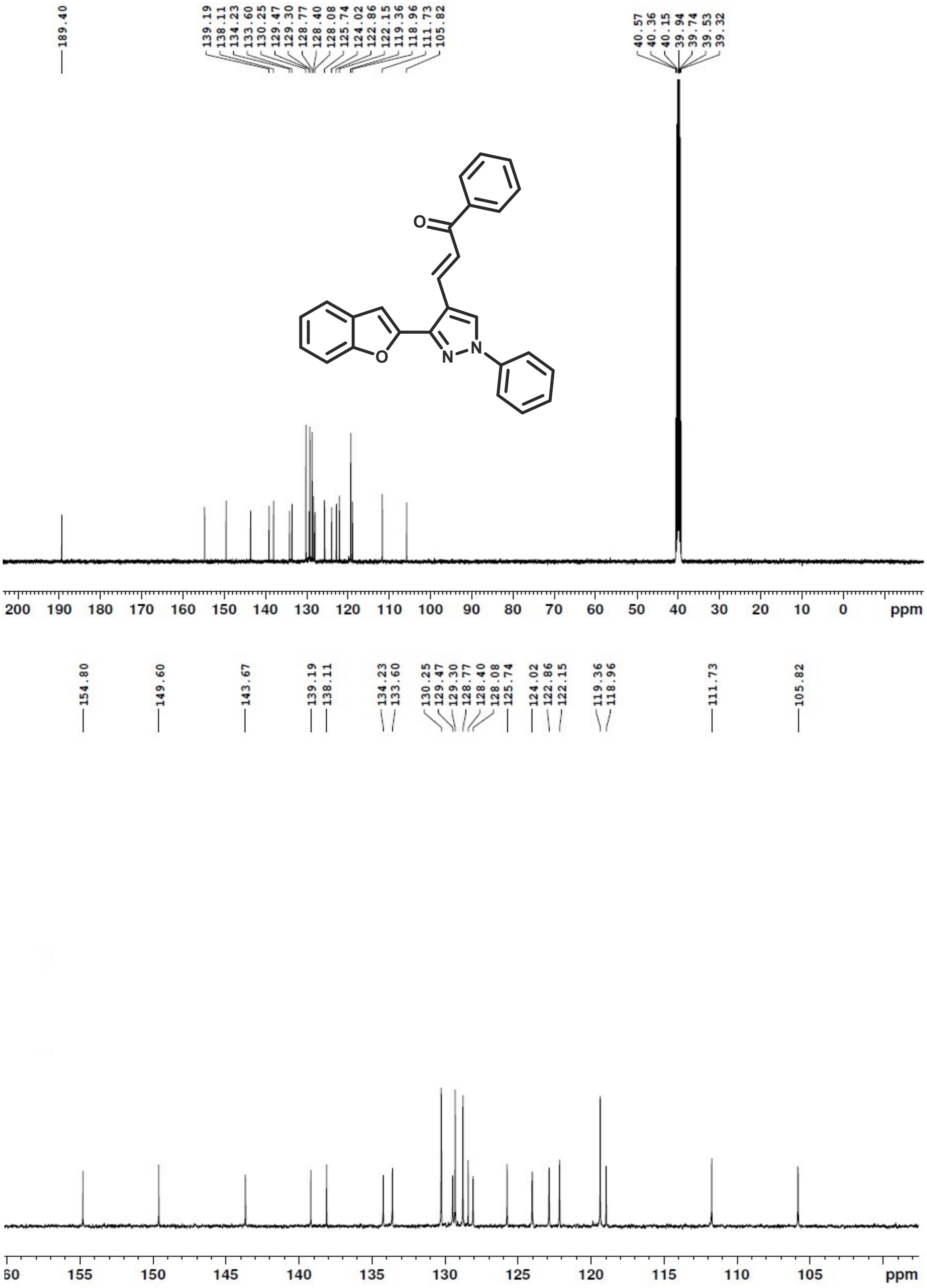

Figure S2: ^{13}CNMR (100 MHz; DMSO-*d6*) spectrum of compound **2a**

3-(5-(Benzofuran-2-yl)-1-phenyl-1H-pyrazol-3-yl)-1-(4-methoxyphenyl)prop-2-en-1-one (2b)

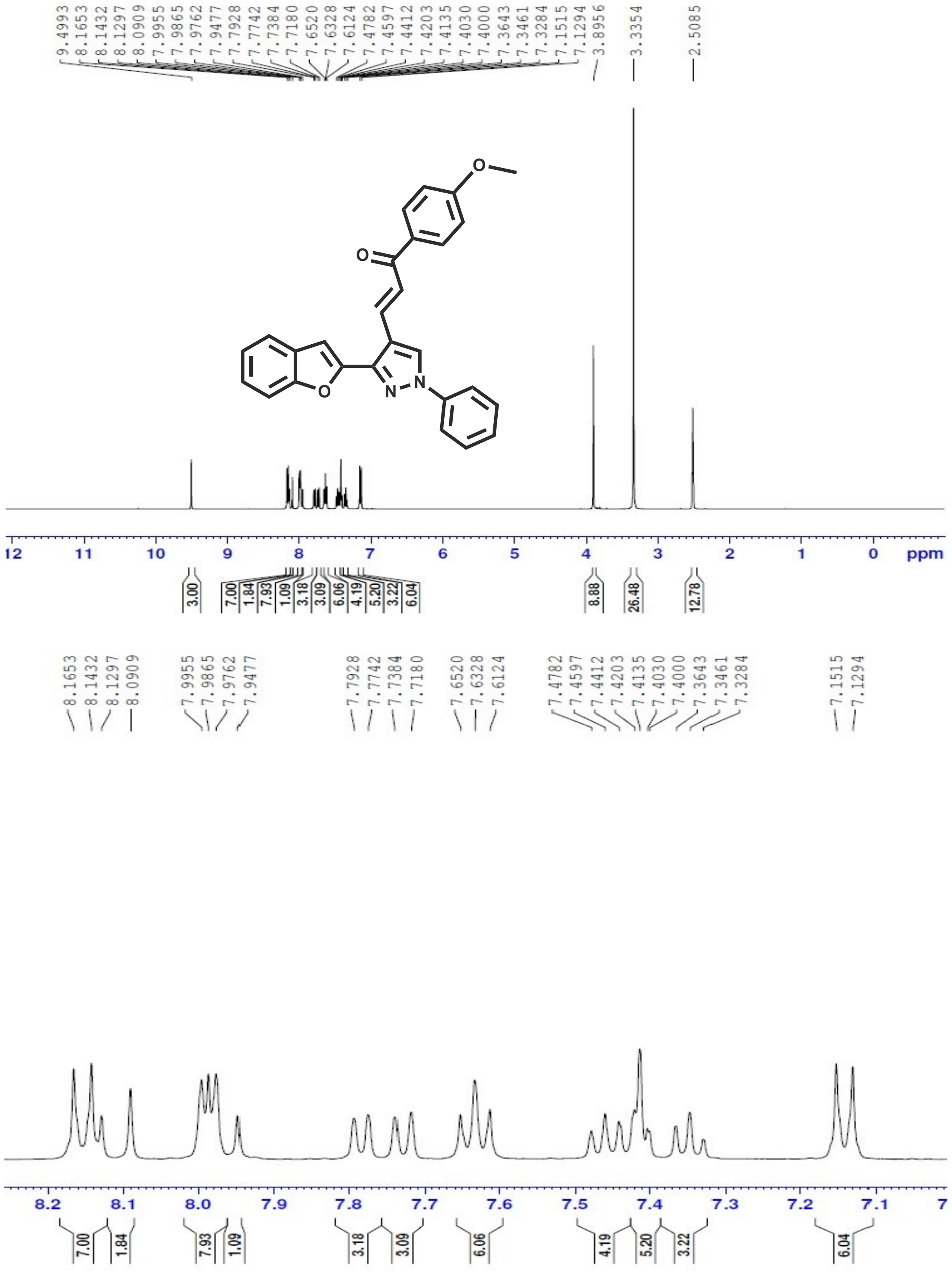

Figure S3: ^{1}HNMR (400 MHz; DMSO-*d6*) spectrum of compound **2b**

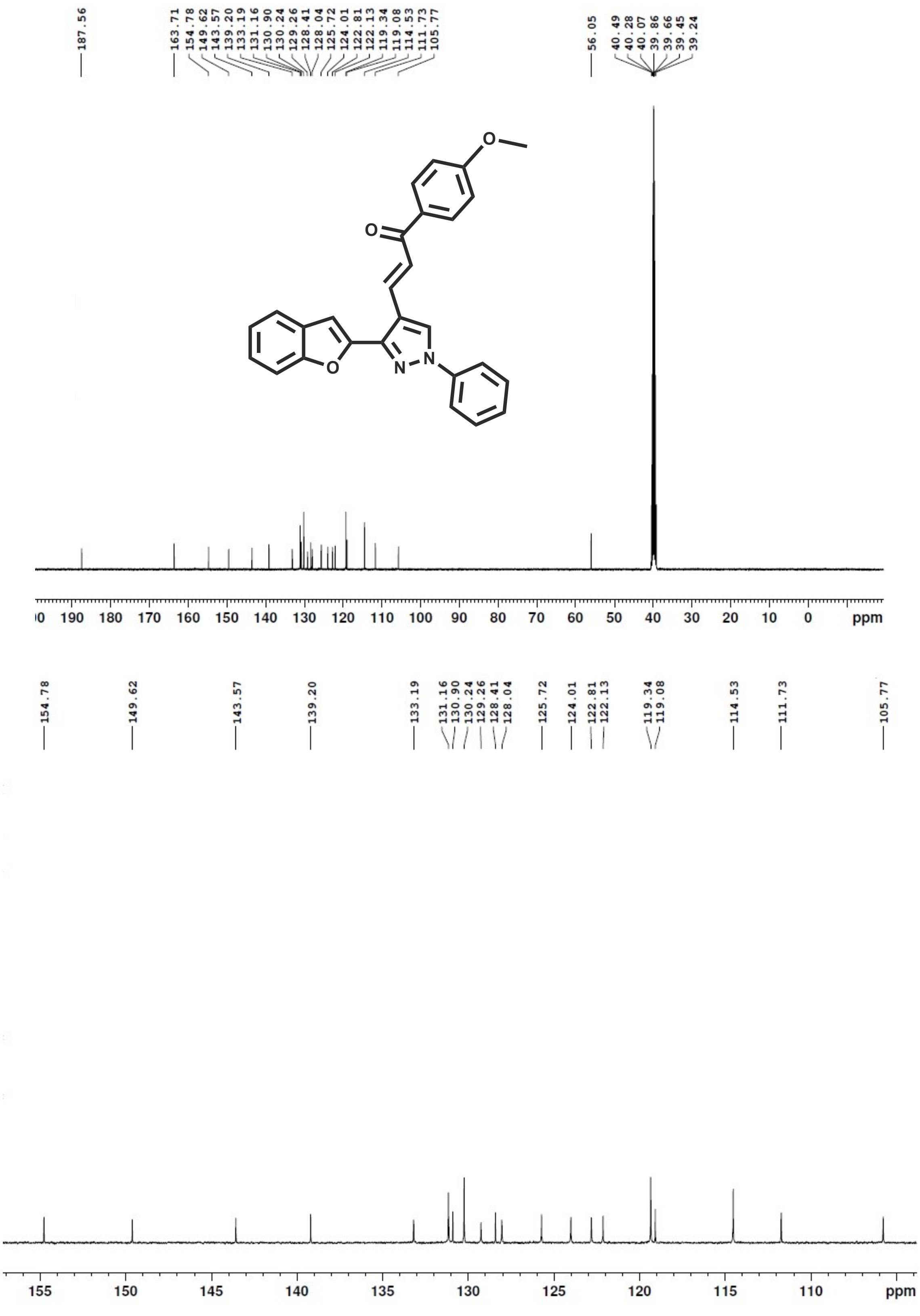

Figure S4: ^{13}CNMR (100 MHz; DMSO-*d6*) spectrum of compound **2b**

3-(5-(Benzofuran-2-yl)-1-phenyl-1H-pyrazol-3-yl)-1-(3,4-dimethoxyphenyl)prop-2-en-1-one (2c)

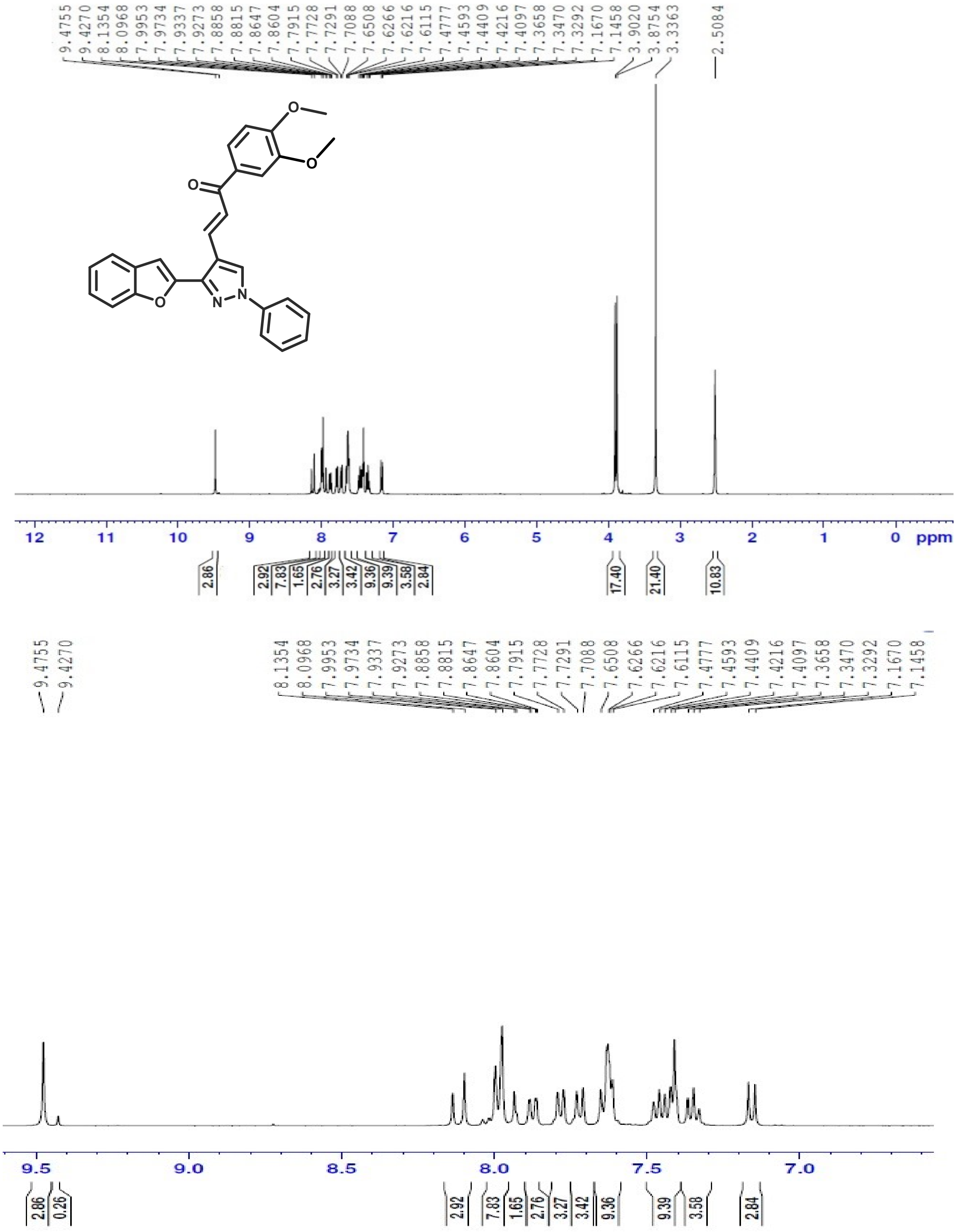

Figure S5: ^{1}HNMR (400 MHz; DMSO-*d6*) spectrum of compound **2c**

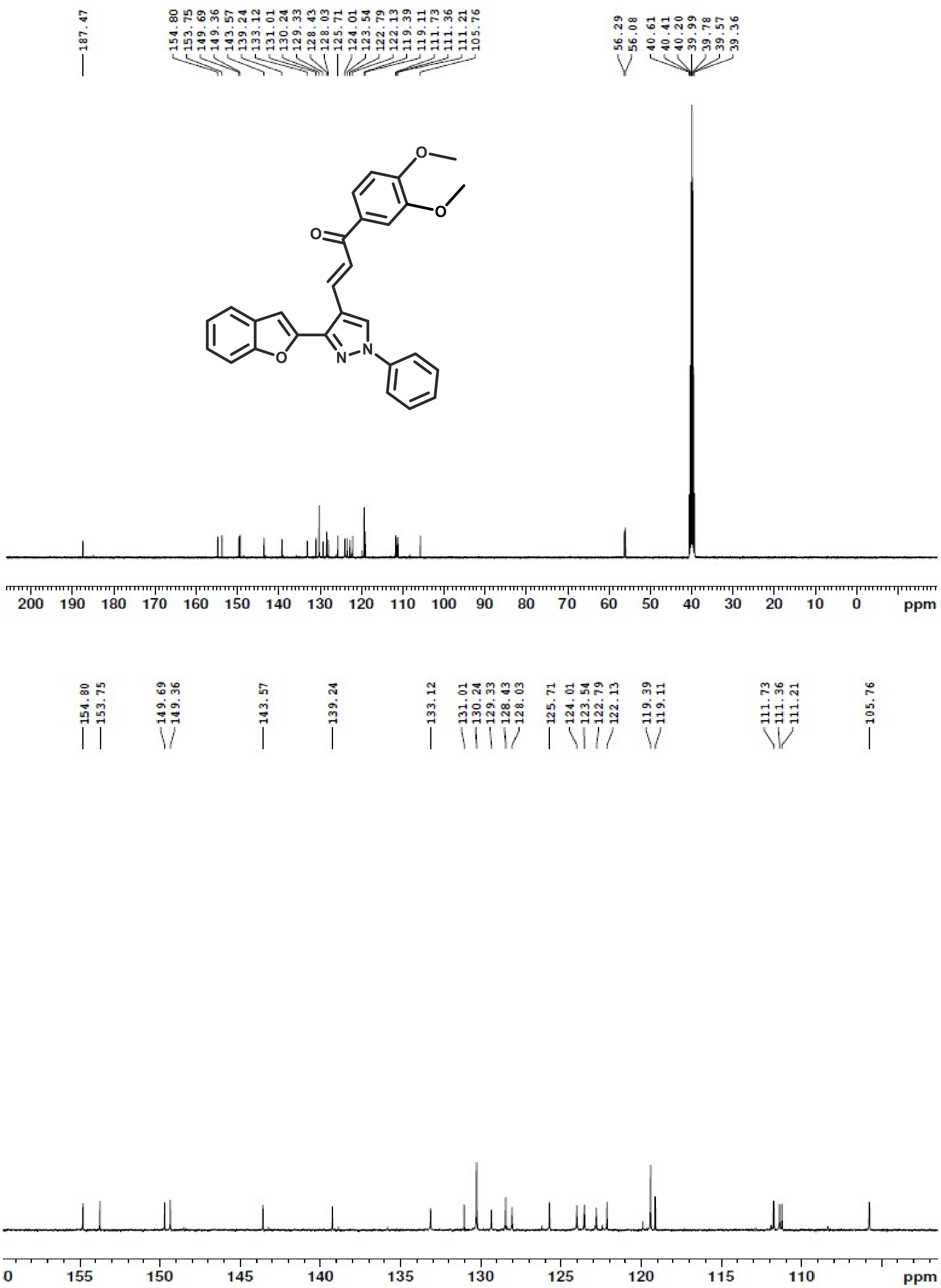

Figure S6: ^{13}CNMR (100 MHz; DMSO-*d6*) spectrum of compound **2c**

1-(1H-Benzo[d]imidazol-2-yl)-3-(5-(benzofuran-2-yl)-1-phenyl-1H-pyrazol-3-yl)prop-2-en-1-one (2d)

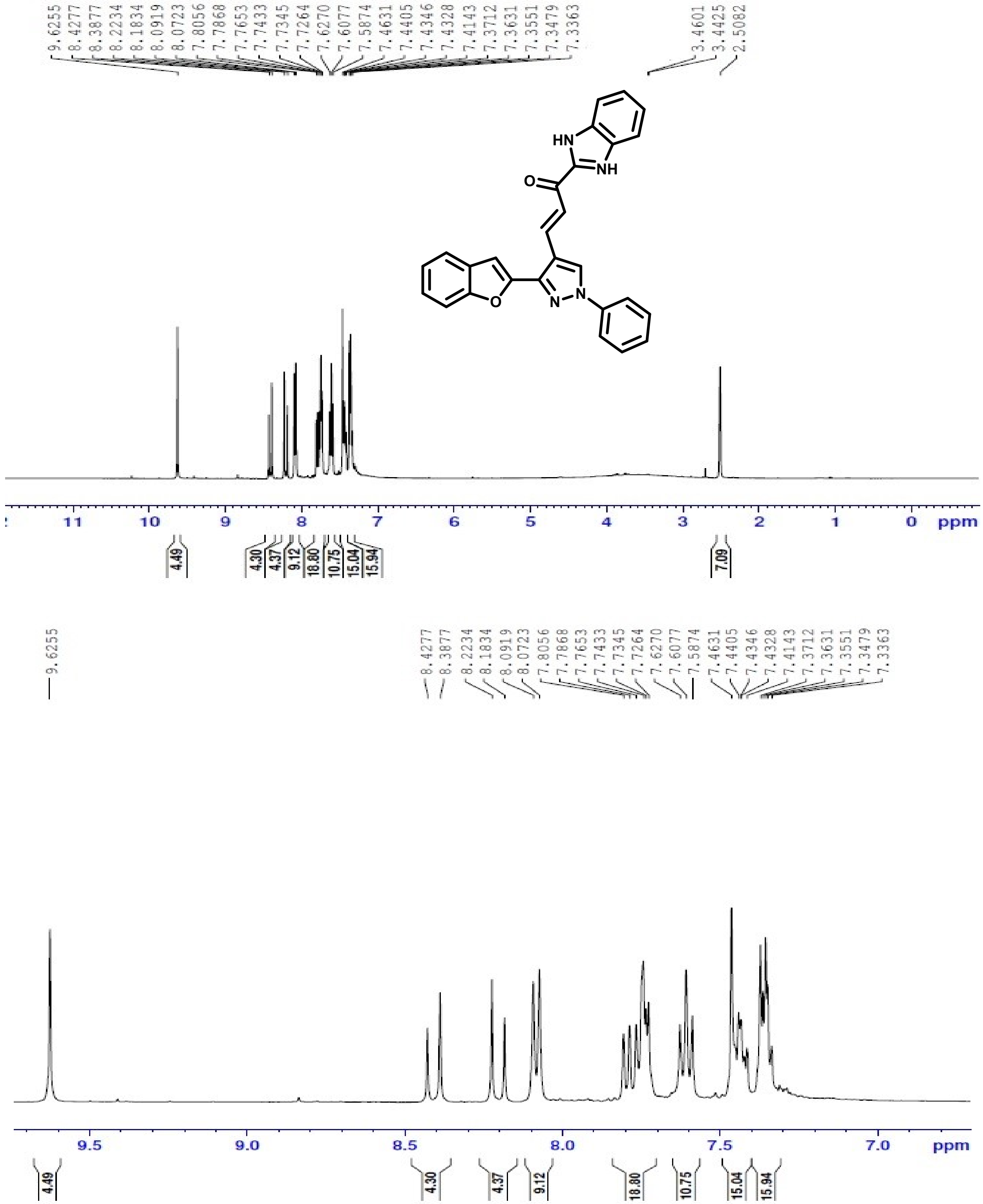

Figure S7: ^{1}HNMR (400 MHz; DMSO-*d6*) spectrum of compound **2d**

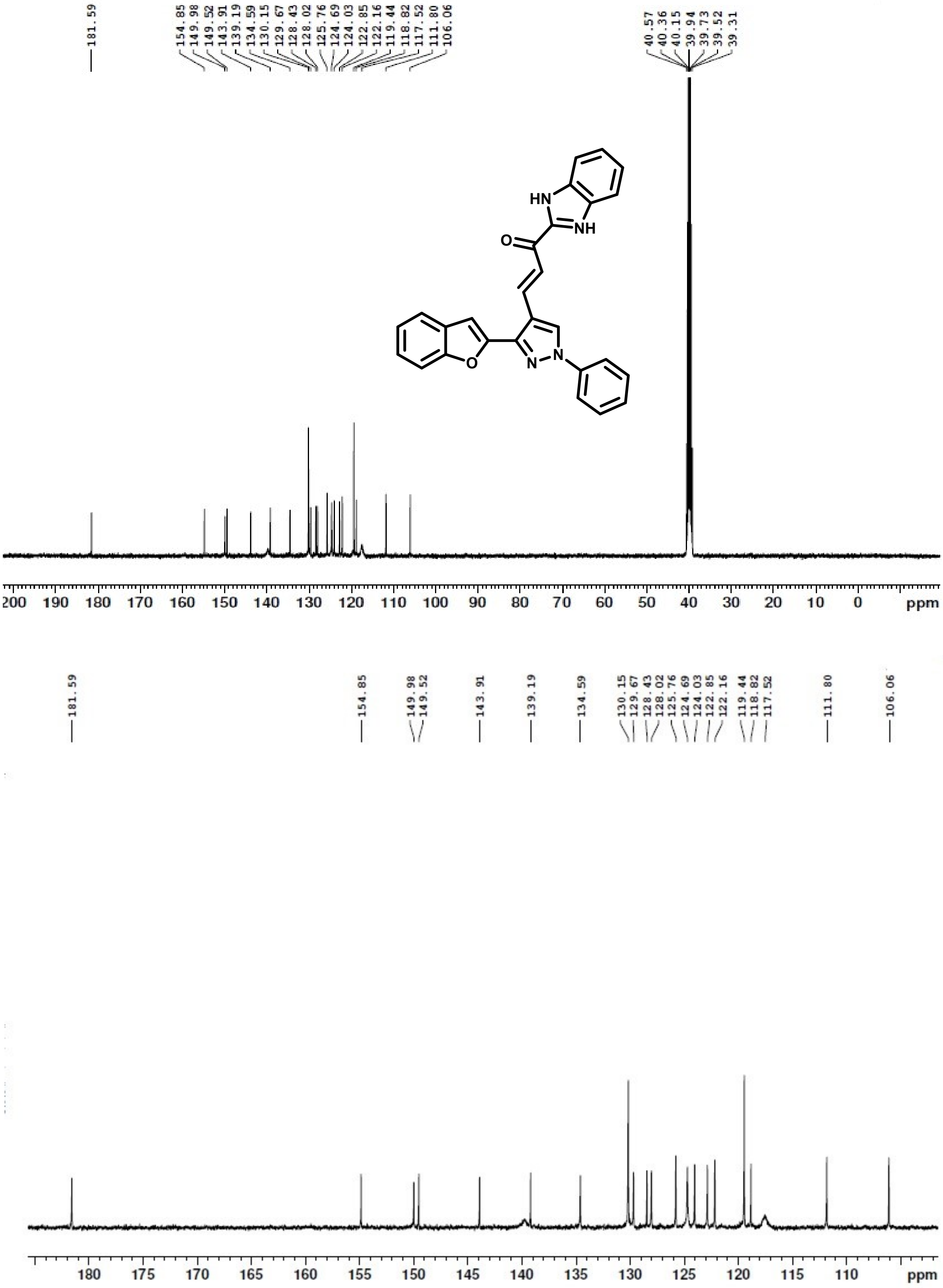

Figure S8: ^{13}CNMR (100 MHz; DMSO-*d6*) spectrum of compound **2d**

3-(Benzofuran-2-yl)-4-(3-phenyl-4,5-dihydro-1H-pyrazol-5-yl)-1-phenyl-1H-pyrazole (3a)

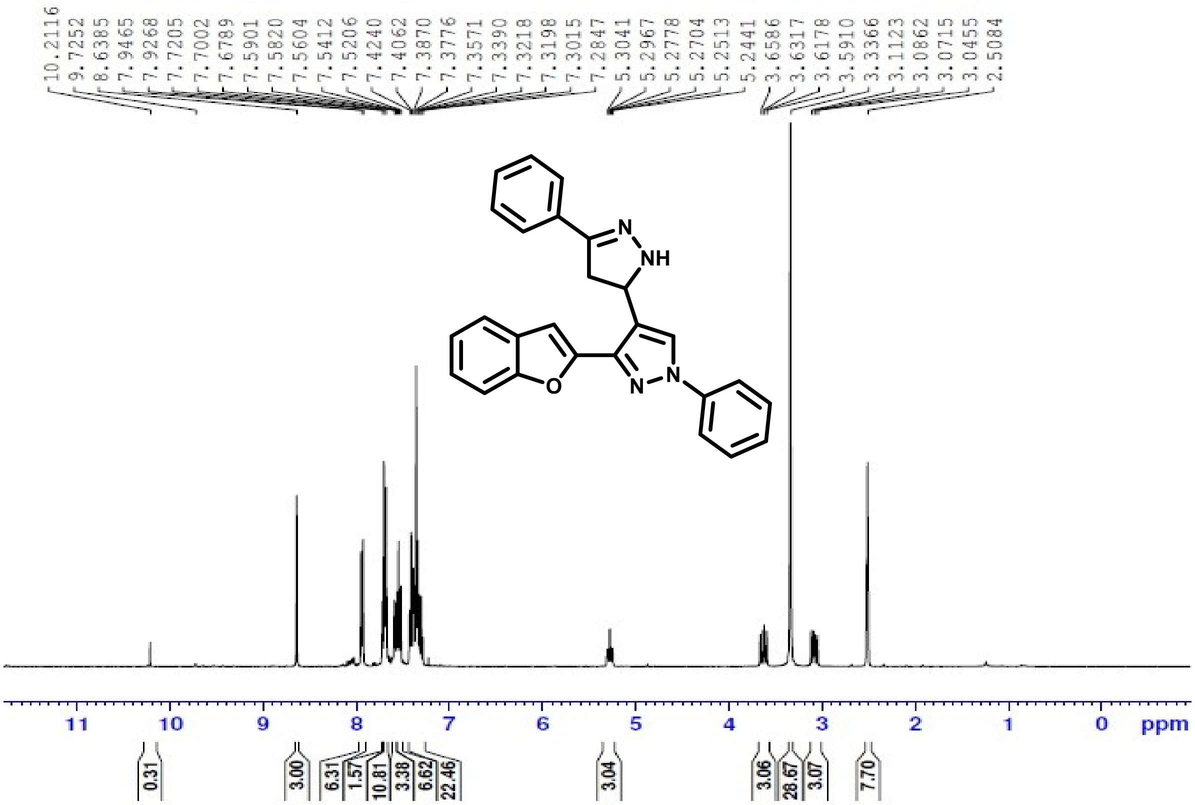

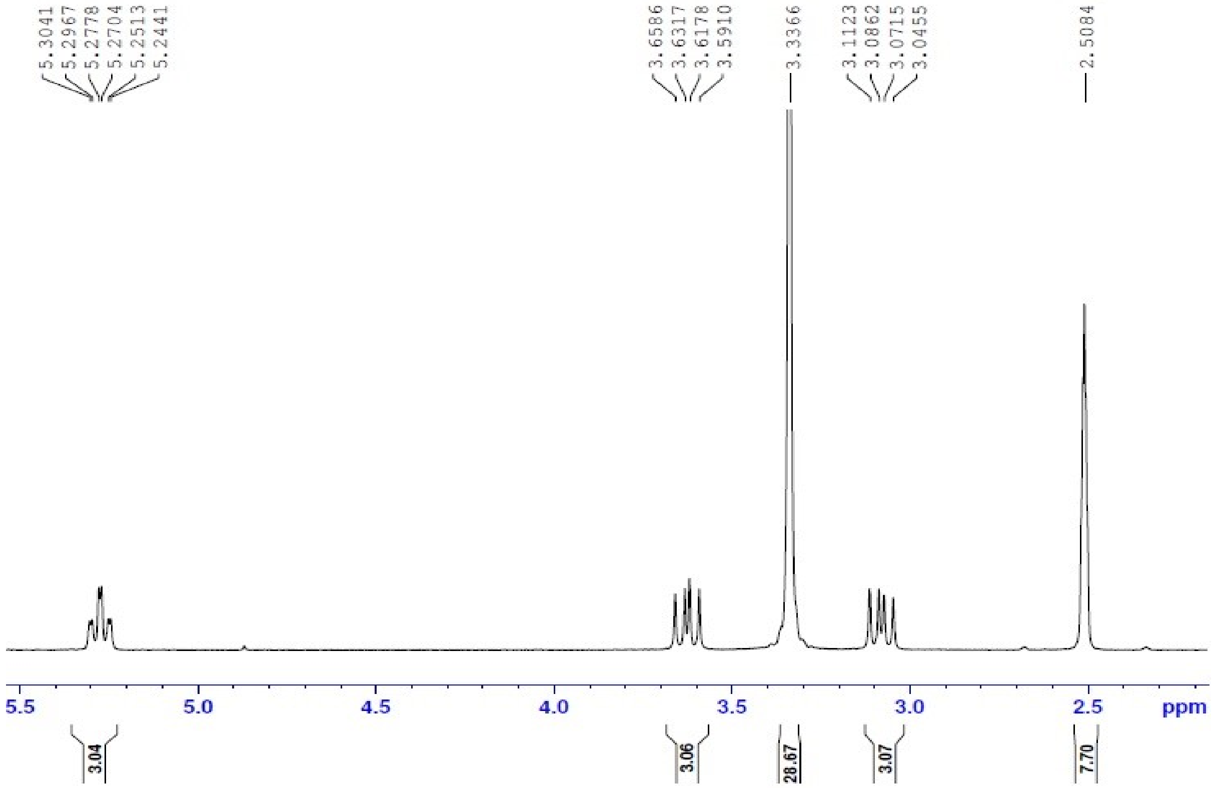

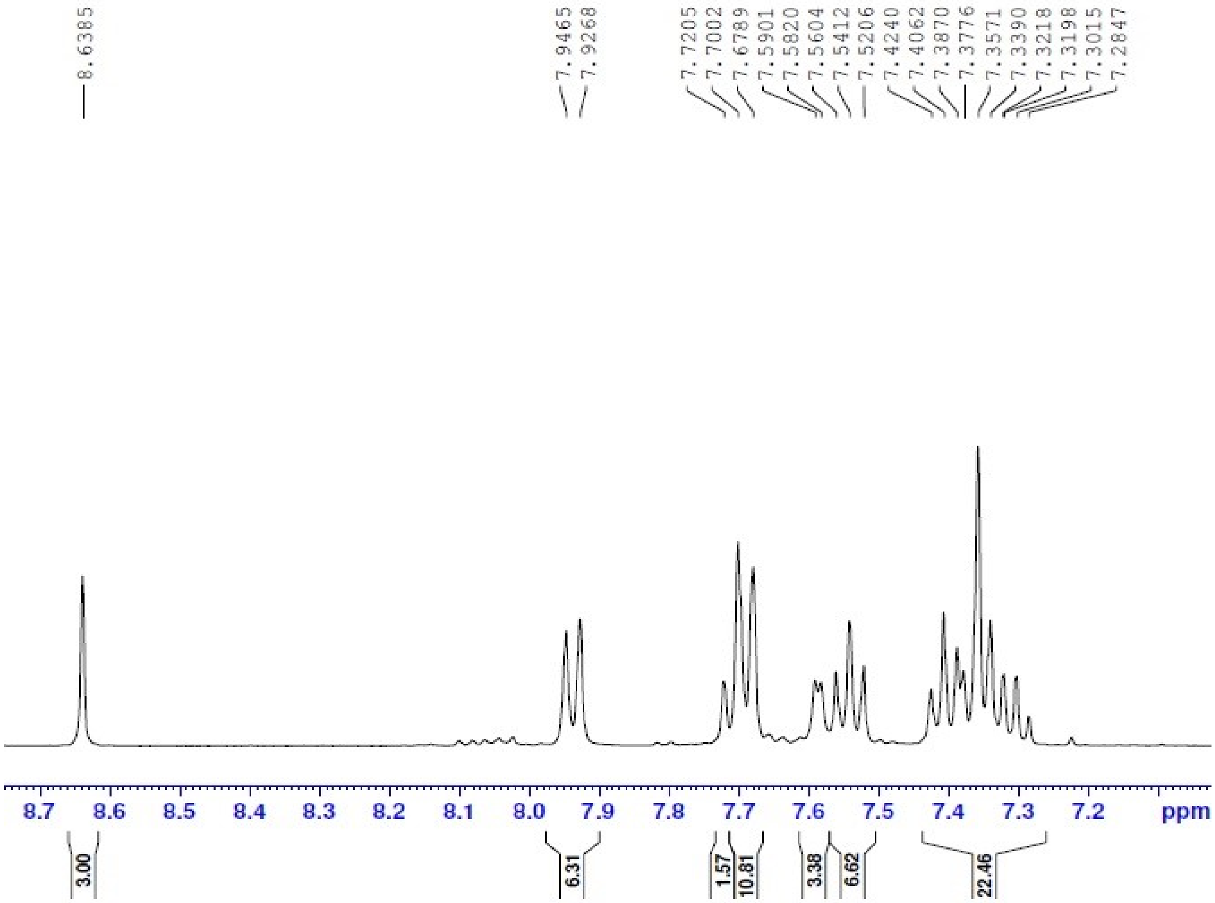

Figure S9: ^{1}HNMR (400 MHz; DMSO-*d6*) spectrum of compound **3a**

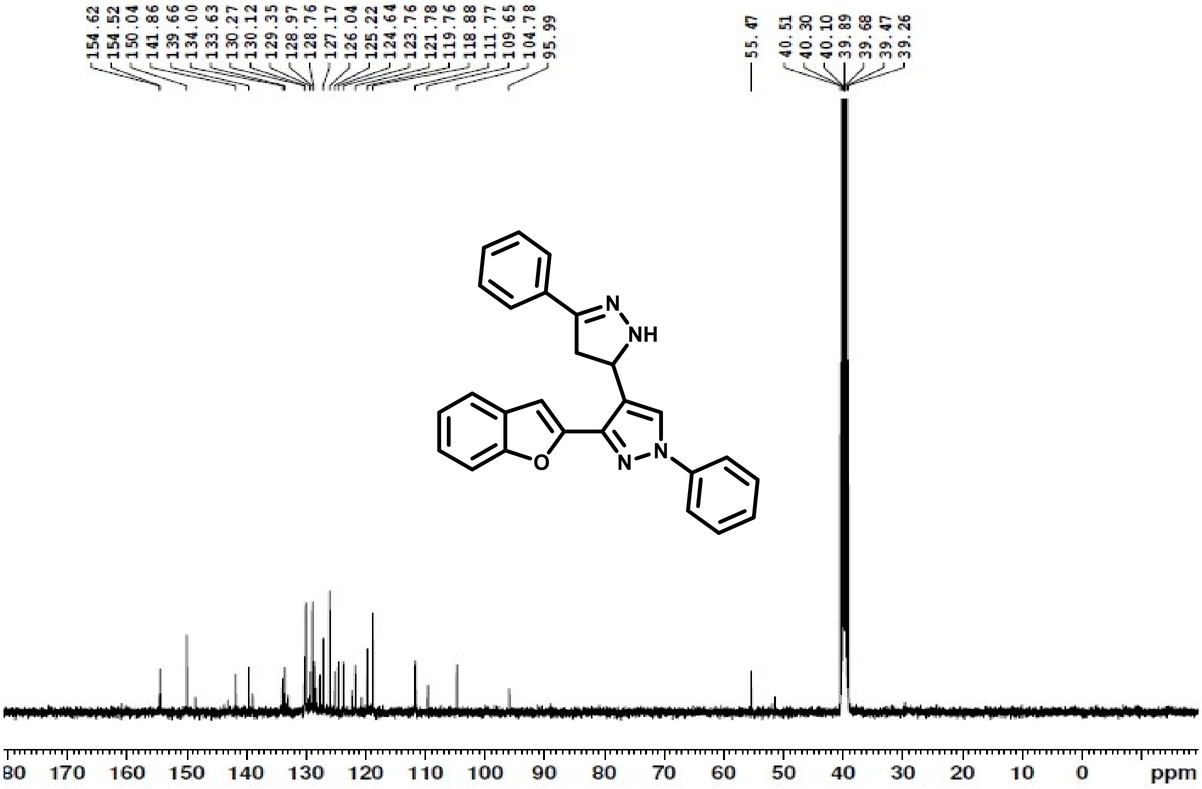

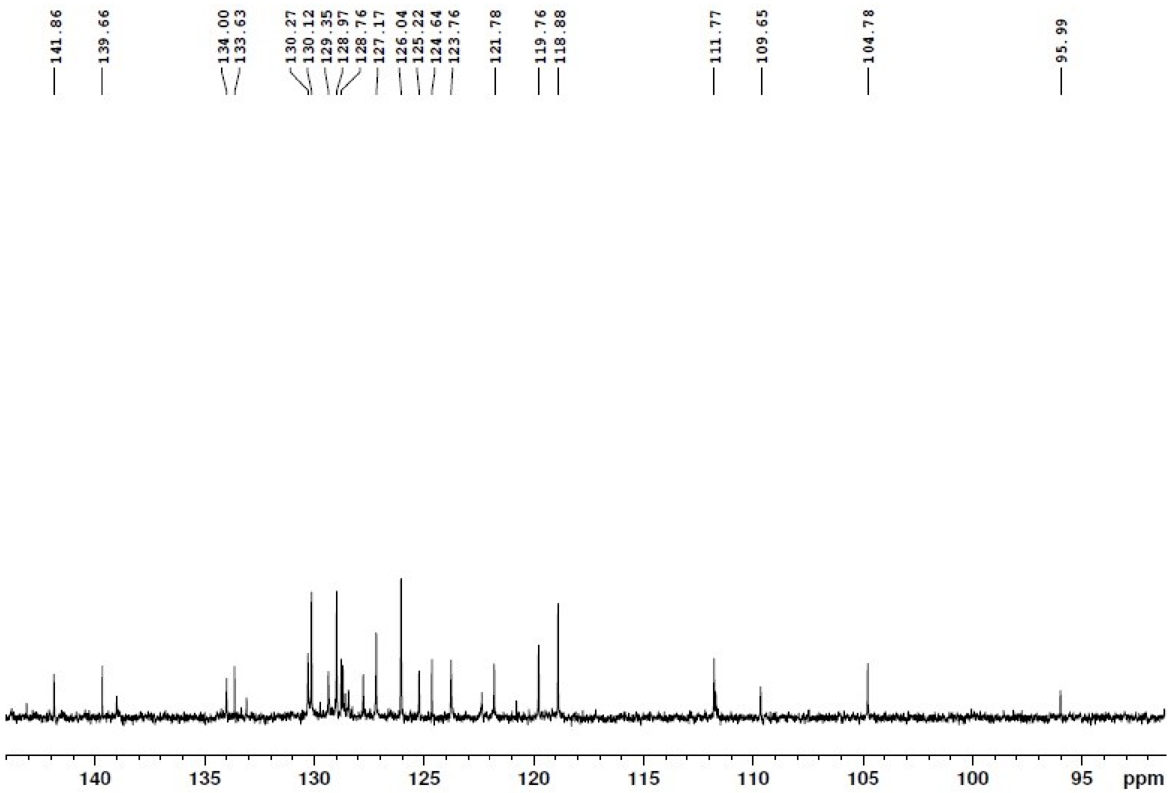

Figure S10: ^{13}CNMR (100 MHz; DMSO-*d6*) spectrum of compound **3a**

3-(Benzofuran-2-yl)-4-(3-(4-methoxyphenyl)-4,5-dihydro-1H-pyrazol-5-yl)-1-phenyl-1H-pyrazole (3b)

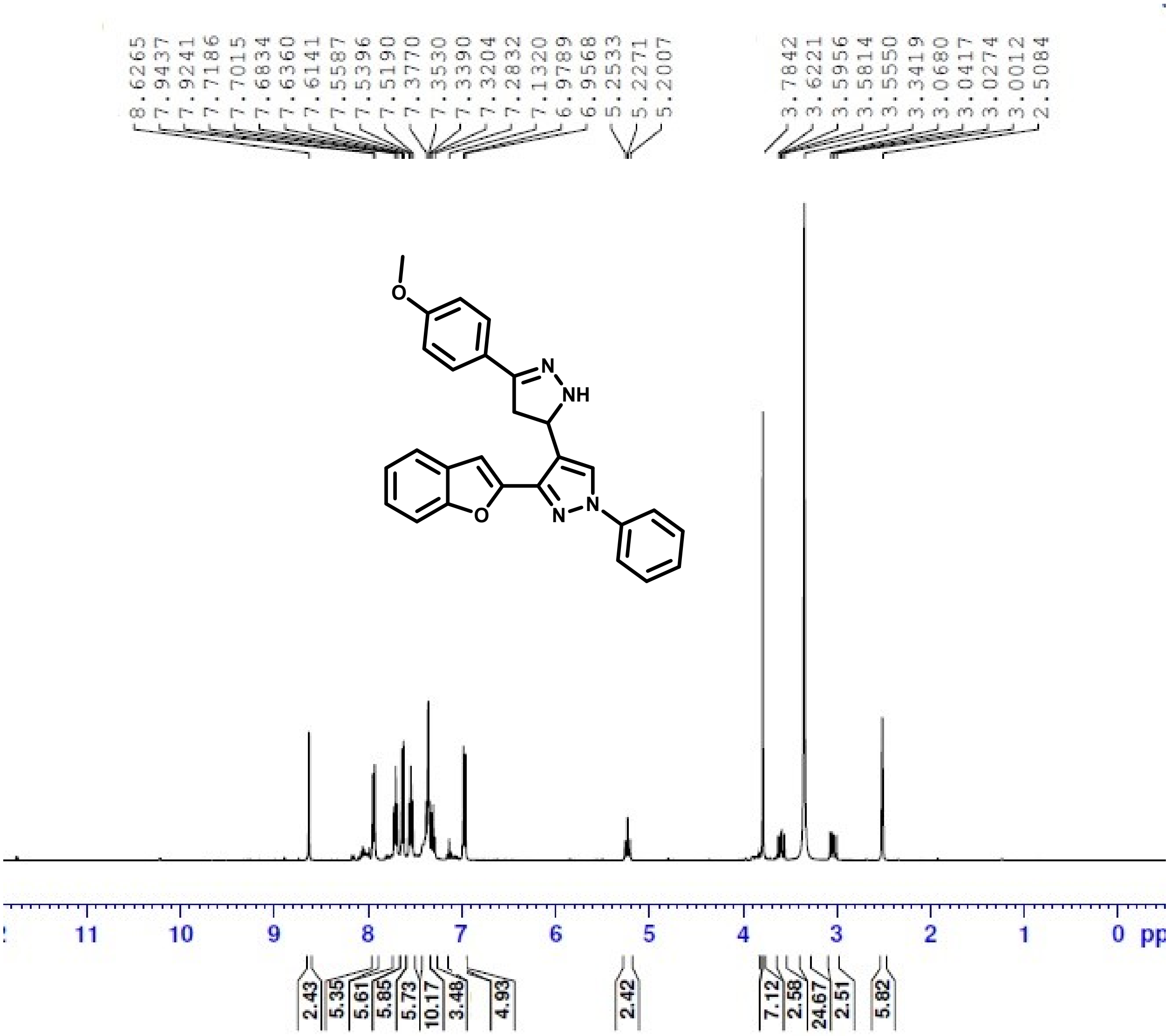

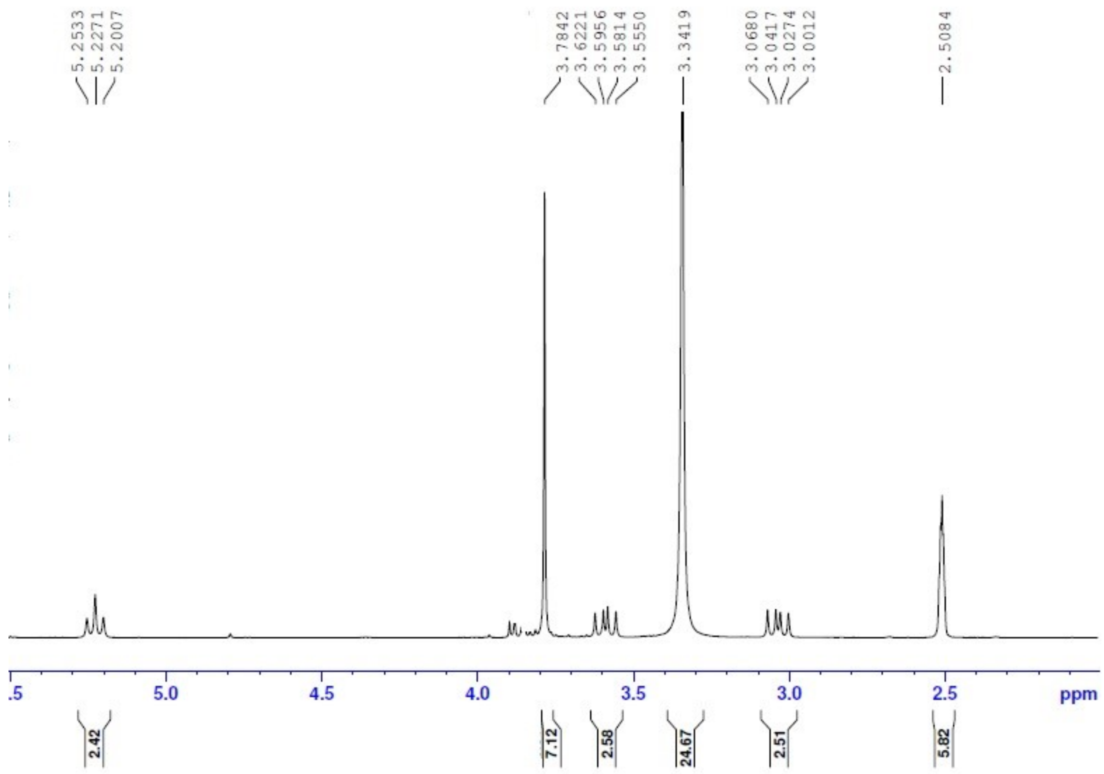

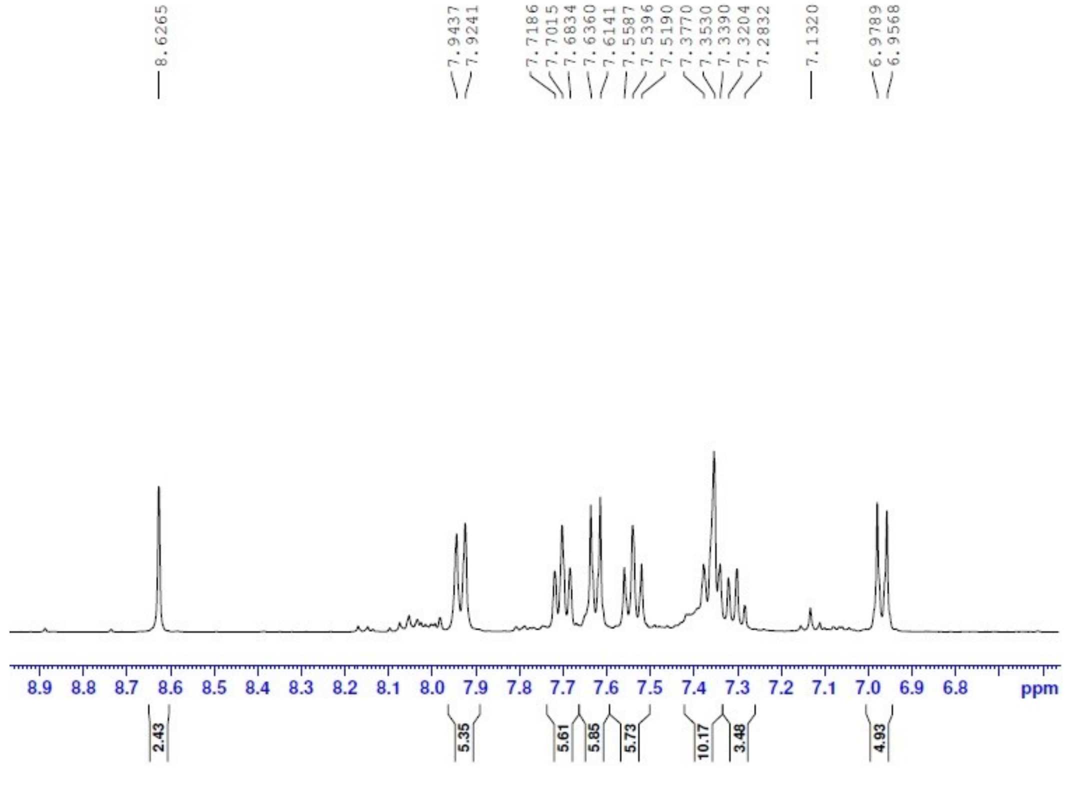

Figure S11: ^{1}HNMR (400 MHz; DMSO-*d6*) spectrum of compound **3b**

3-(Benzofuran-2-yl)-4-(3-(3,4-dimethoxyphenyl)-4,5-dihydro-1H-pyrazol-5-yl)-1-phenyl-1H-pyrazole (3c)

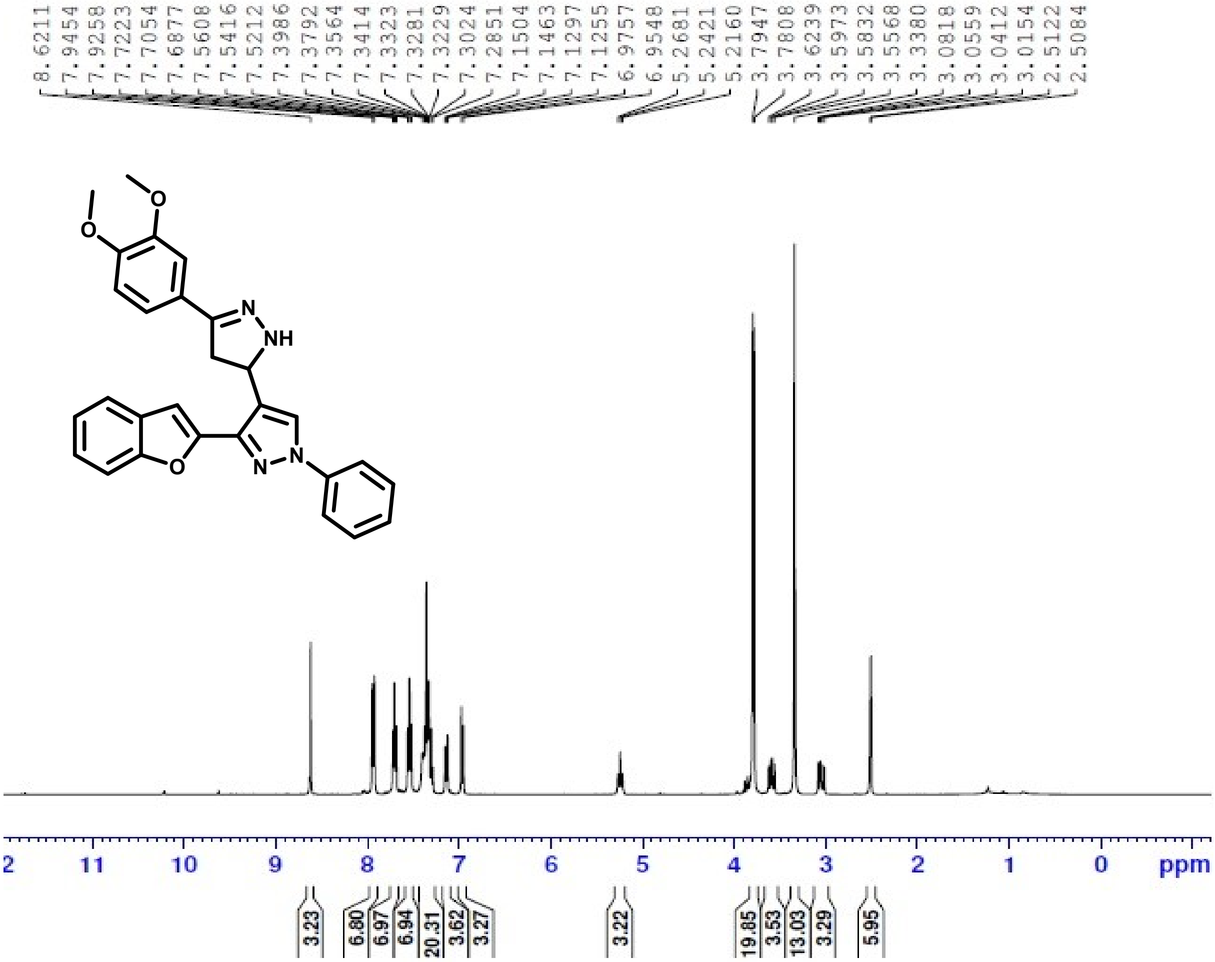

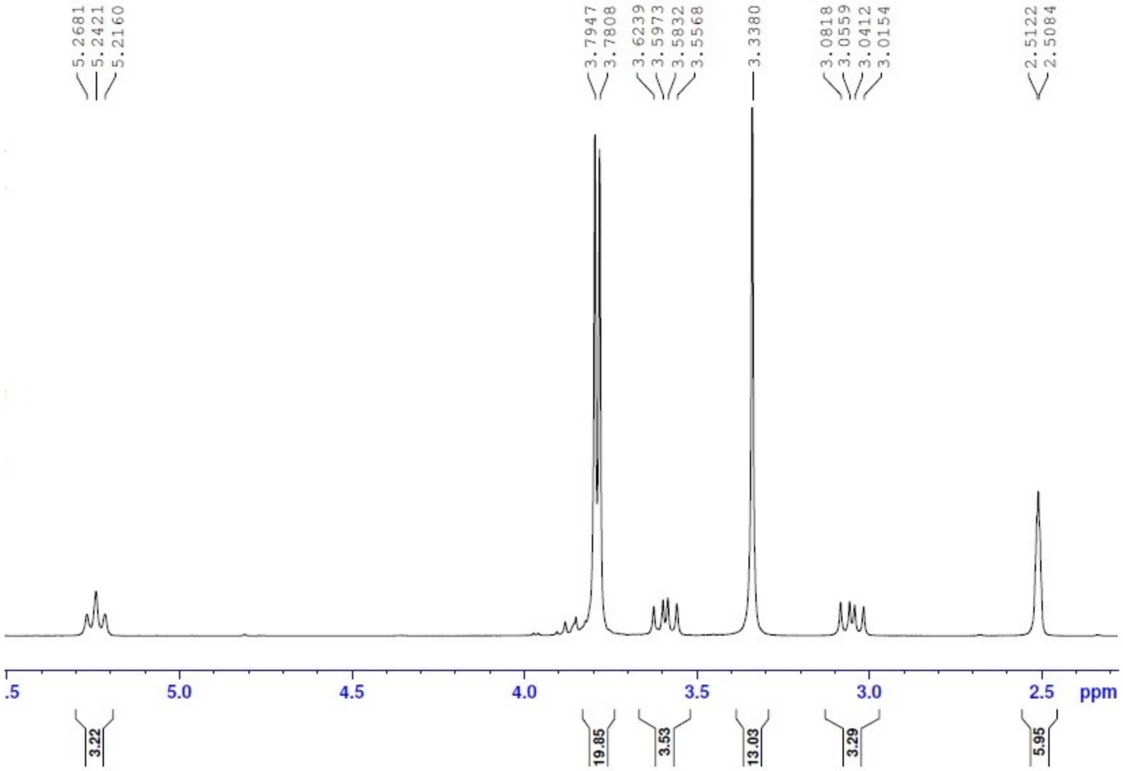

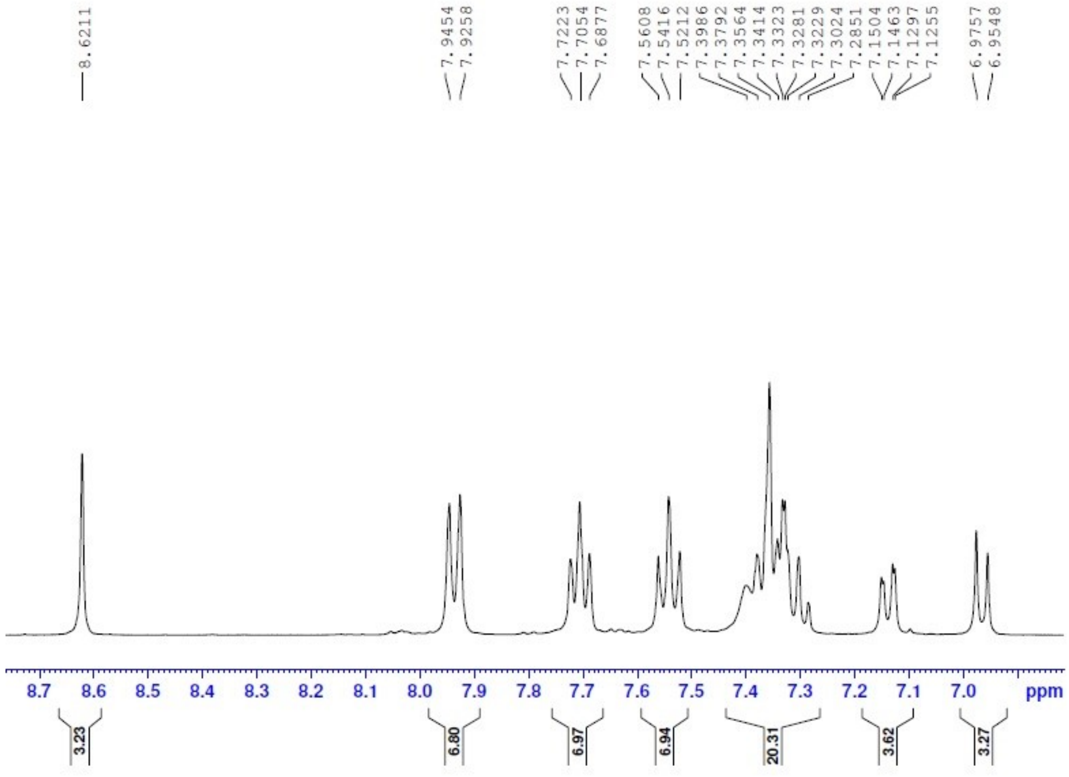

Figure S12: ^{1}HNMR (400 MHz; DMSO-*d6*) spectrum of compound **3c**

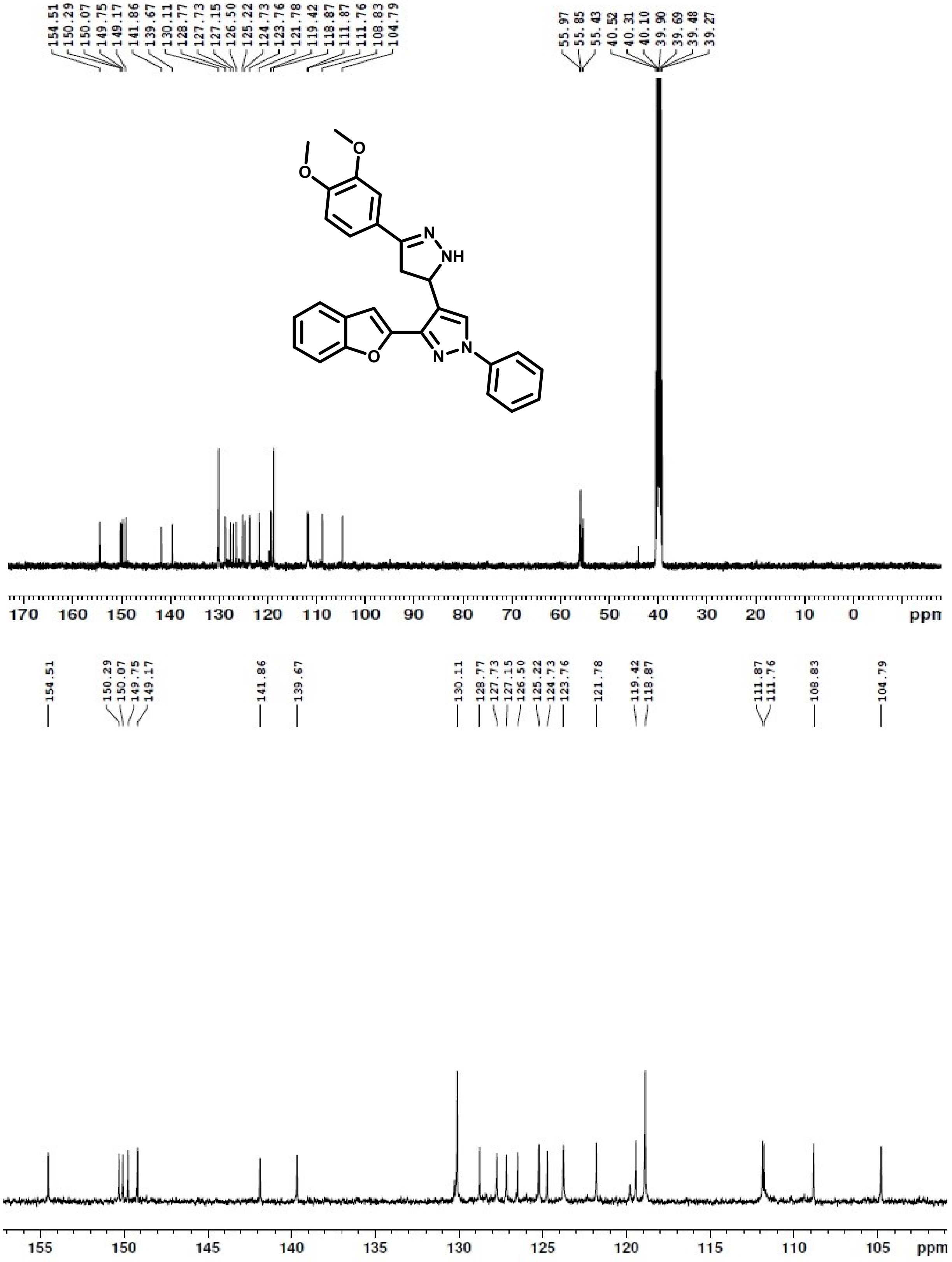

Figure S13: ^{13}CNMR (100 MHz; DMSO-*d6*) spectrum of compound **3c**

3-(Benzofuran-2-yl)-4-(3-(1H-benzo[d]imidazol-2-yl)-4,5-dihydro-1H-pyrazol-5-yl)-1-phenyl-1H-pyrazole (3d)

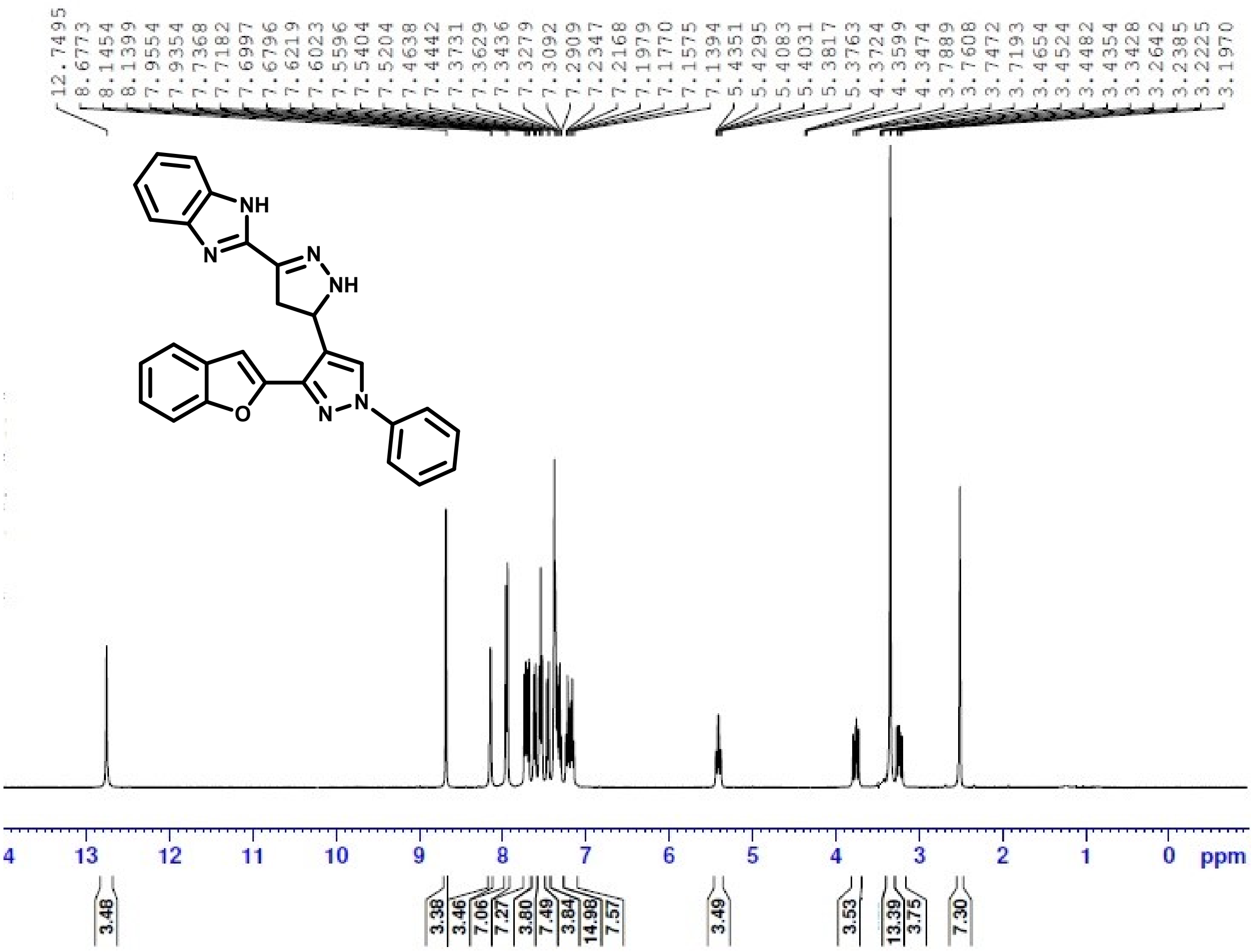

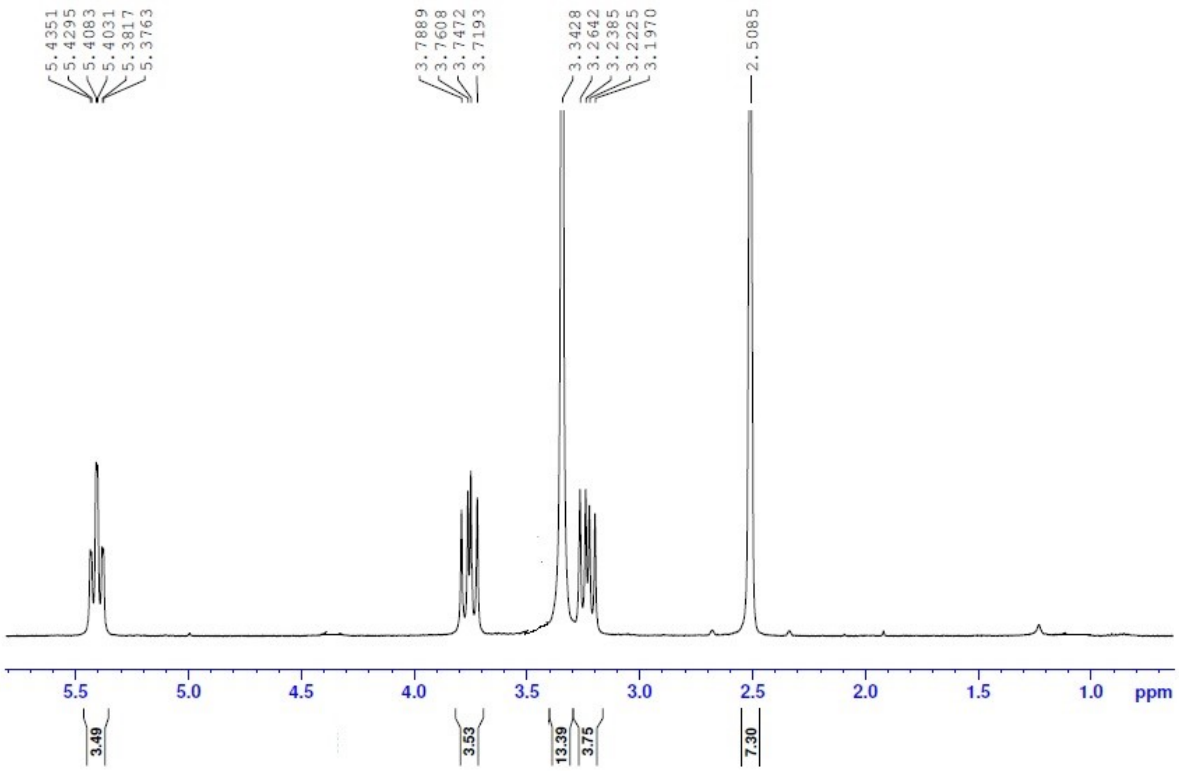

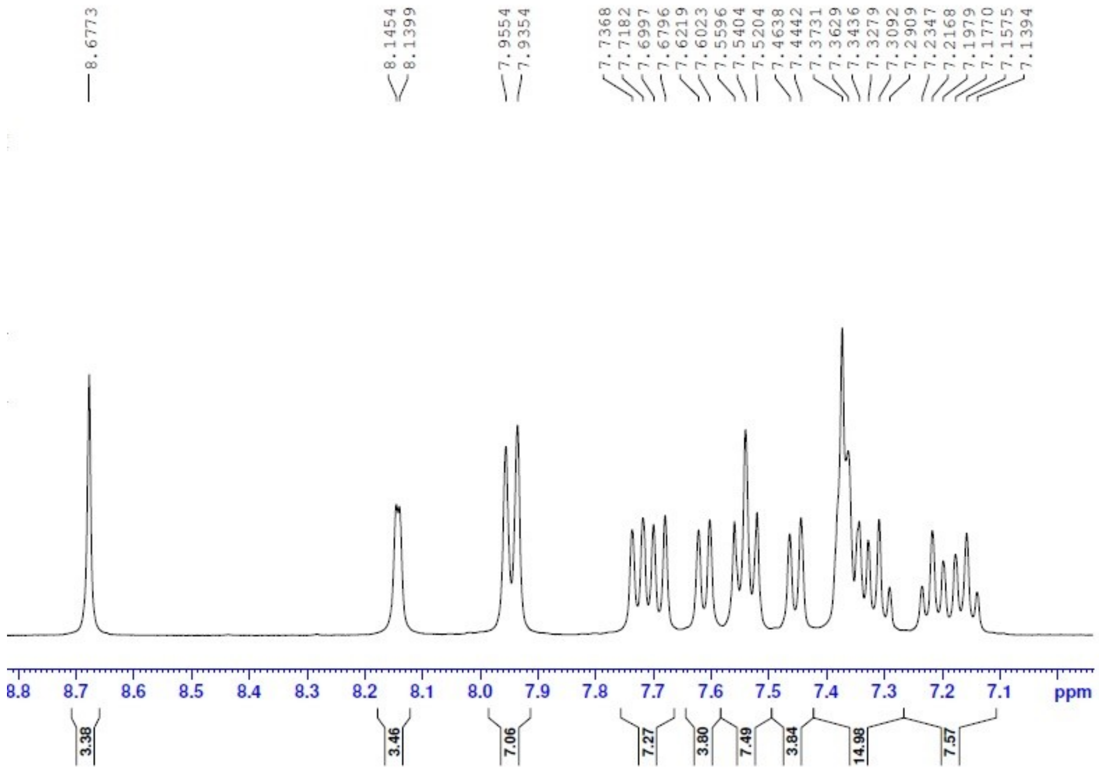

Figure S14: ^{1}HNMR (400 MHz; DMSO-*d6*) spectrum of compound **3d**

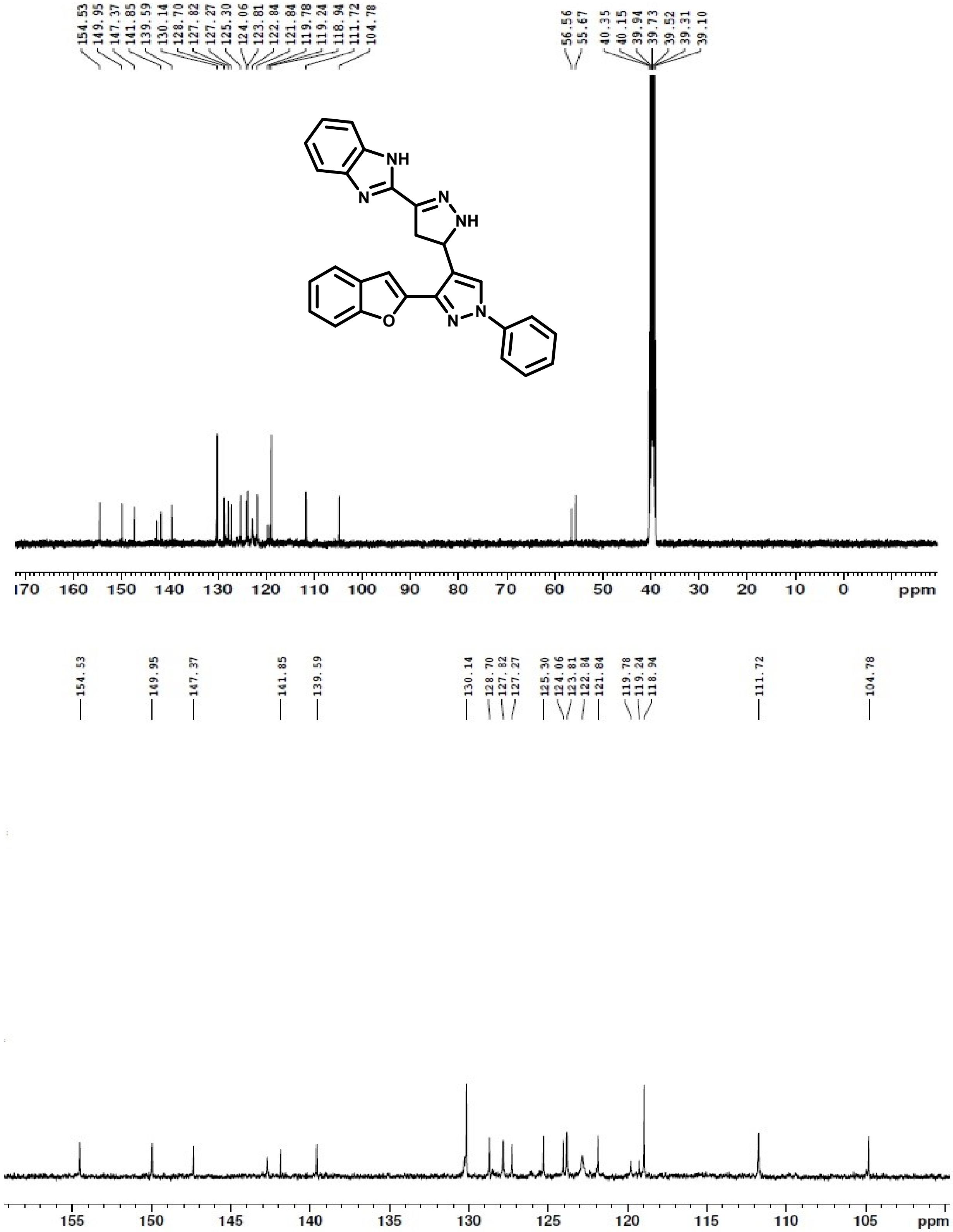

Figure S15: ^{13}CNMR (100 MHz; DMSO-*d6*) spectrum of compound **3d**

1-(5-(3-(Benzofuran-2-yl)-1-phenyl-1H-pyrazol-4-yl)-3-phenyl-4,5-dihydropyrazol-1-yl)ethanone (4a)

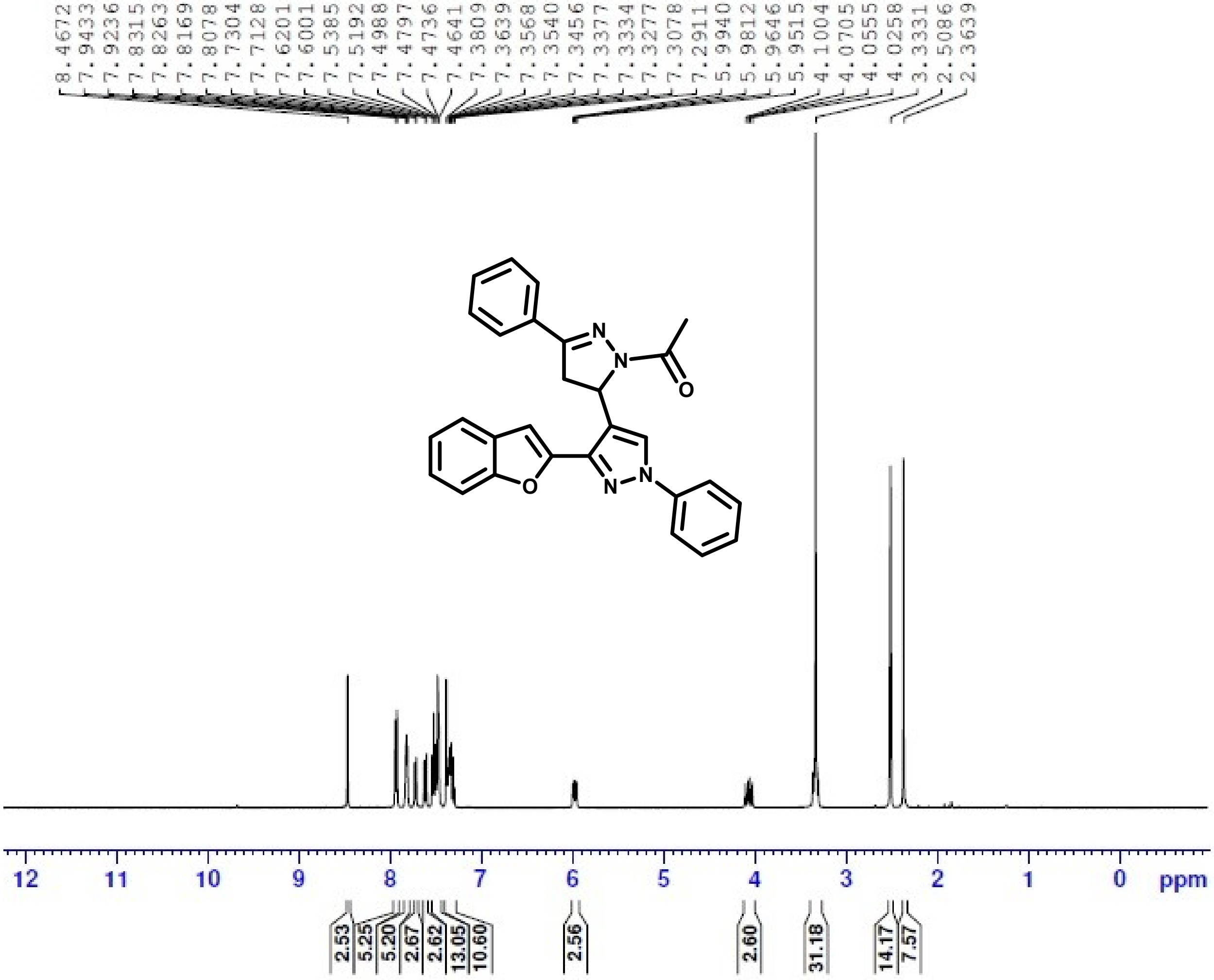

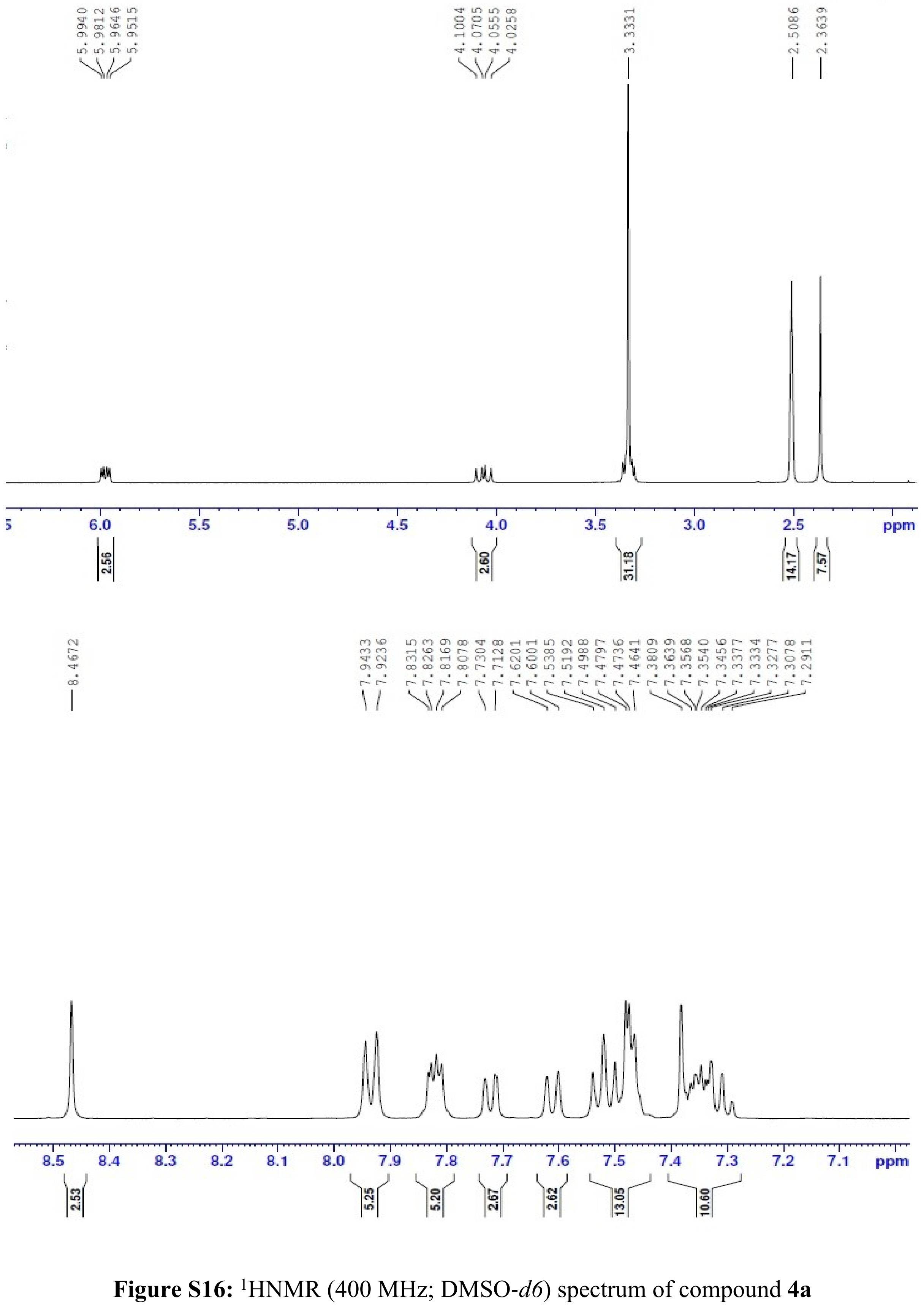

Figure S16: ^{1}HNMR (400 MHz; DMSO-*d6*) spectrum of compound **4a**

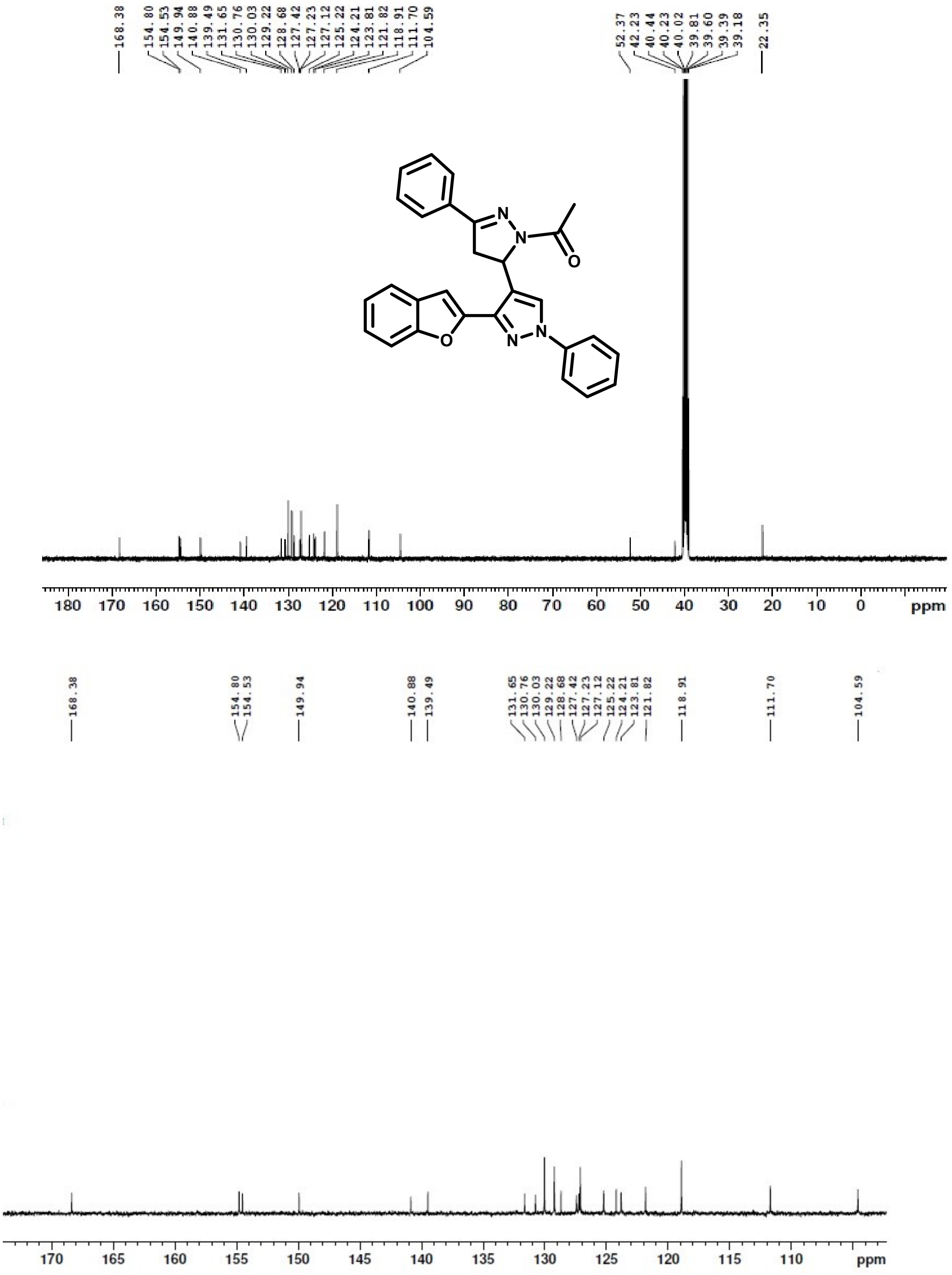

Figure S17: ^{13}CNMR (100 MHz; DMSO-*d6*) spectrum of compound **4a**

1-(5-(3-(Benzofuran-2-yl)-1-phenyl-1H-pyrazol-4-yl)-3-(4-methoxyphenyl)-4,5-dihydropyrazol-1-yl)ethanone (4b)

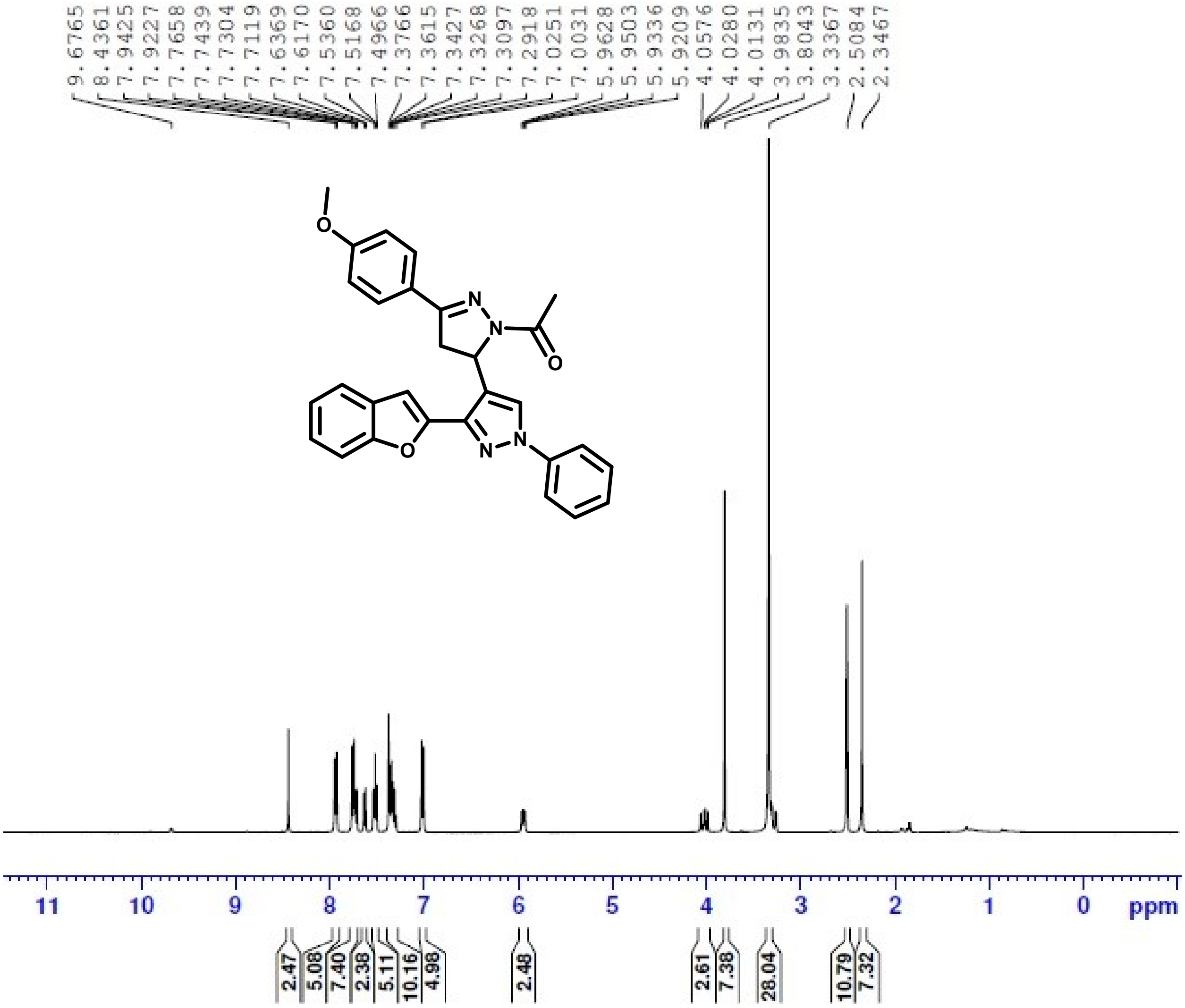

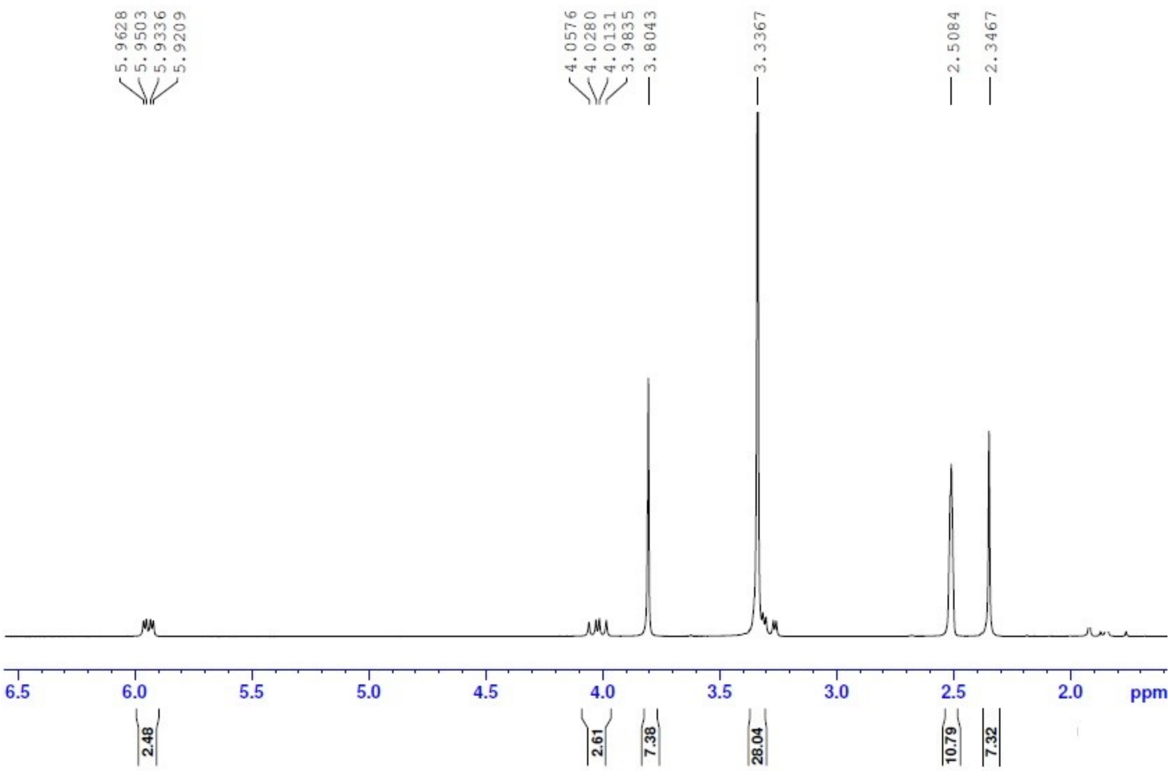

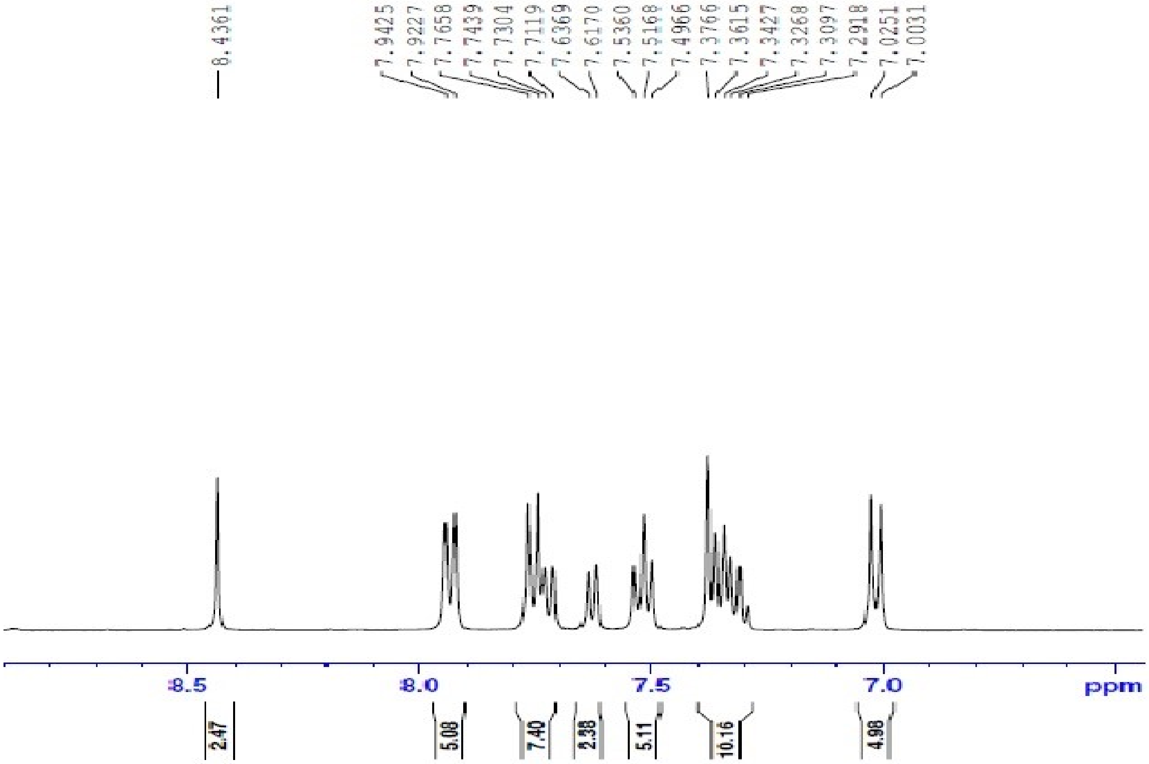

Figure S18: ^{1}HNMR (400 MHz; DMSO-*d6*) spectrum of compound **4b**

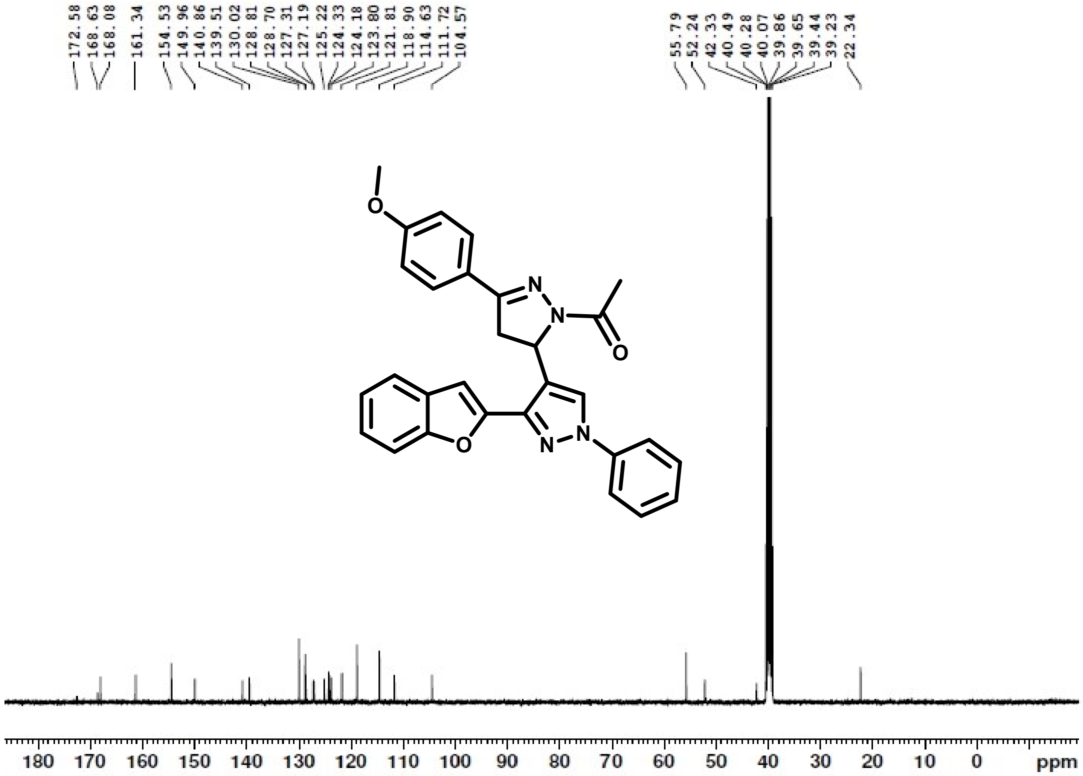
172.58
168.63
168.08
161.34
154.53
149.96
140.86
139.51
130.02
128.81
128.70
127.31
127.19
125.22
124.33
124.18
123.80
121.81
118.90
114.63
111.72
104.57
55.79
52.24
42.33
40.49
40.28
40.07
39.86
39.65
39.44
39.23
22.34
O
N
N
O
O
N
N
180 170 160 150 140 130 120 110 100 90 80 70 60 50 40 30 20 10 0 ppm

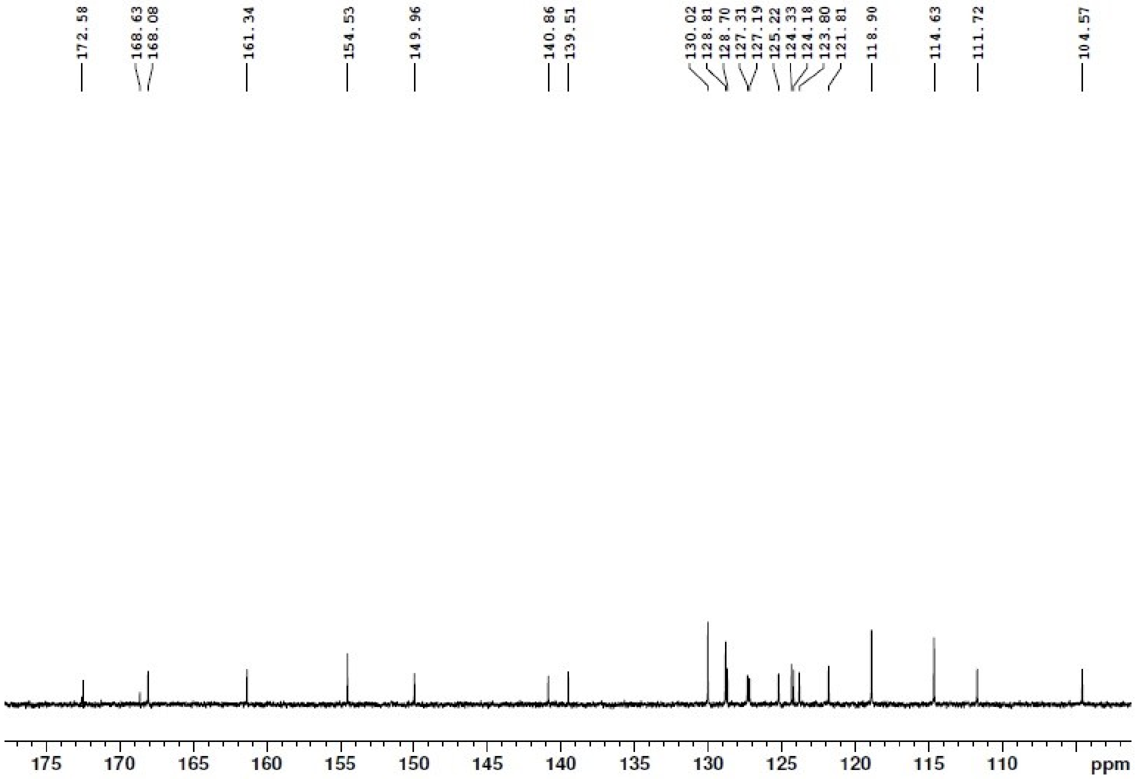
172.58
168.63
168.08
161.34
154.53
149.96
140.86
139.51
130.02
128.81
128.70
127.31
127.19
125.22
124.33
124.18
123.80
121.81
118.90
114.63
111.72
104.57
175 170 165 160 155 150 145 140 135 130 125 120 115 110 ppm

Figure S19: ^{13}CNMR (100 MHz; DMSO-*d6*) spectrum of compound **4b**

1-(5-(3-(Benzofuran-2-yl)-1-phenyl-1H-pyrazol-4-yl)-3-(3,4-dimethoxyphenyl)-4,5-dihydropyrazol-1-yl)ethanone (4c)

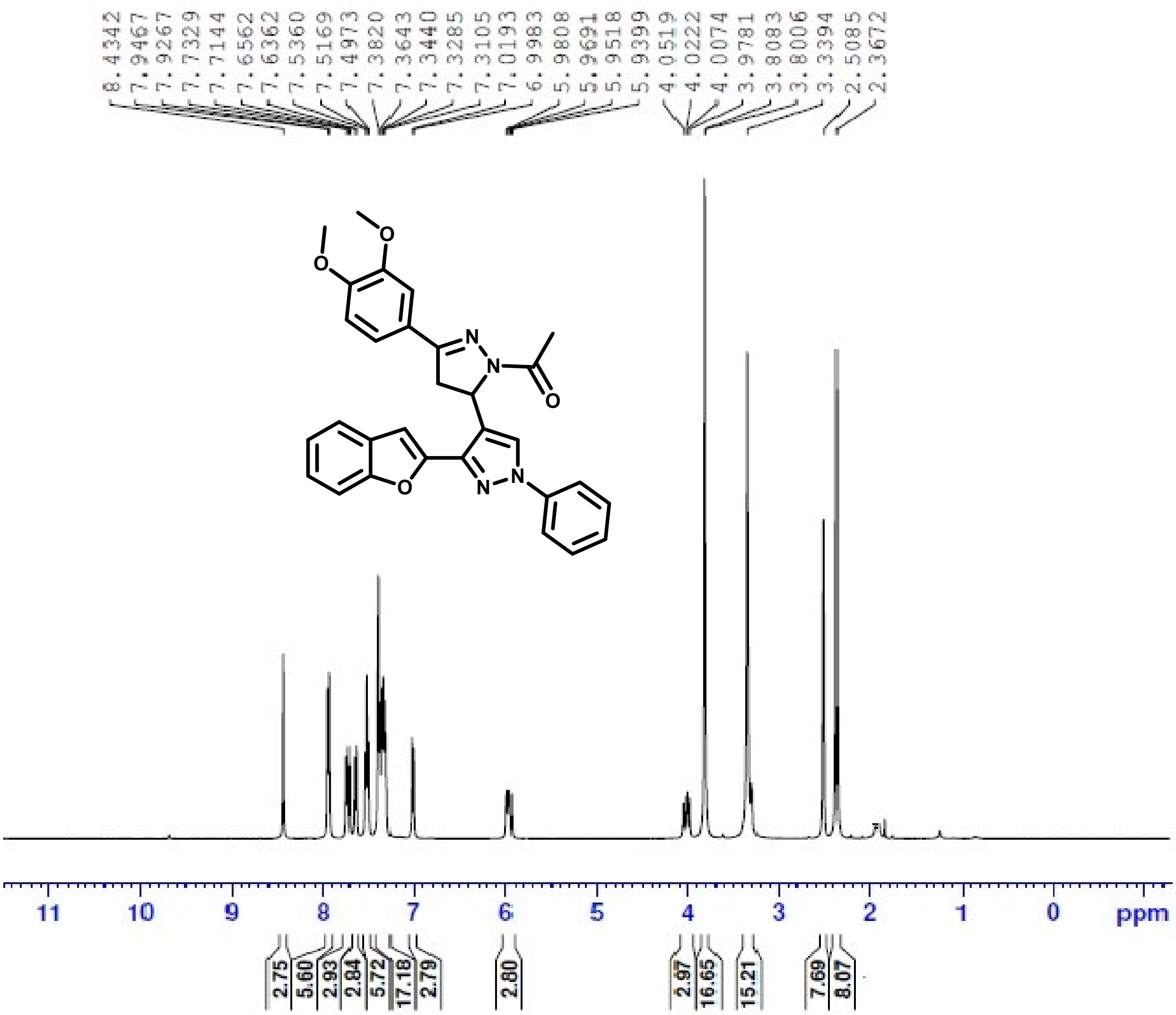

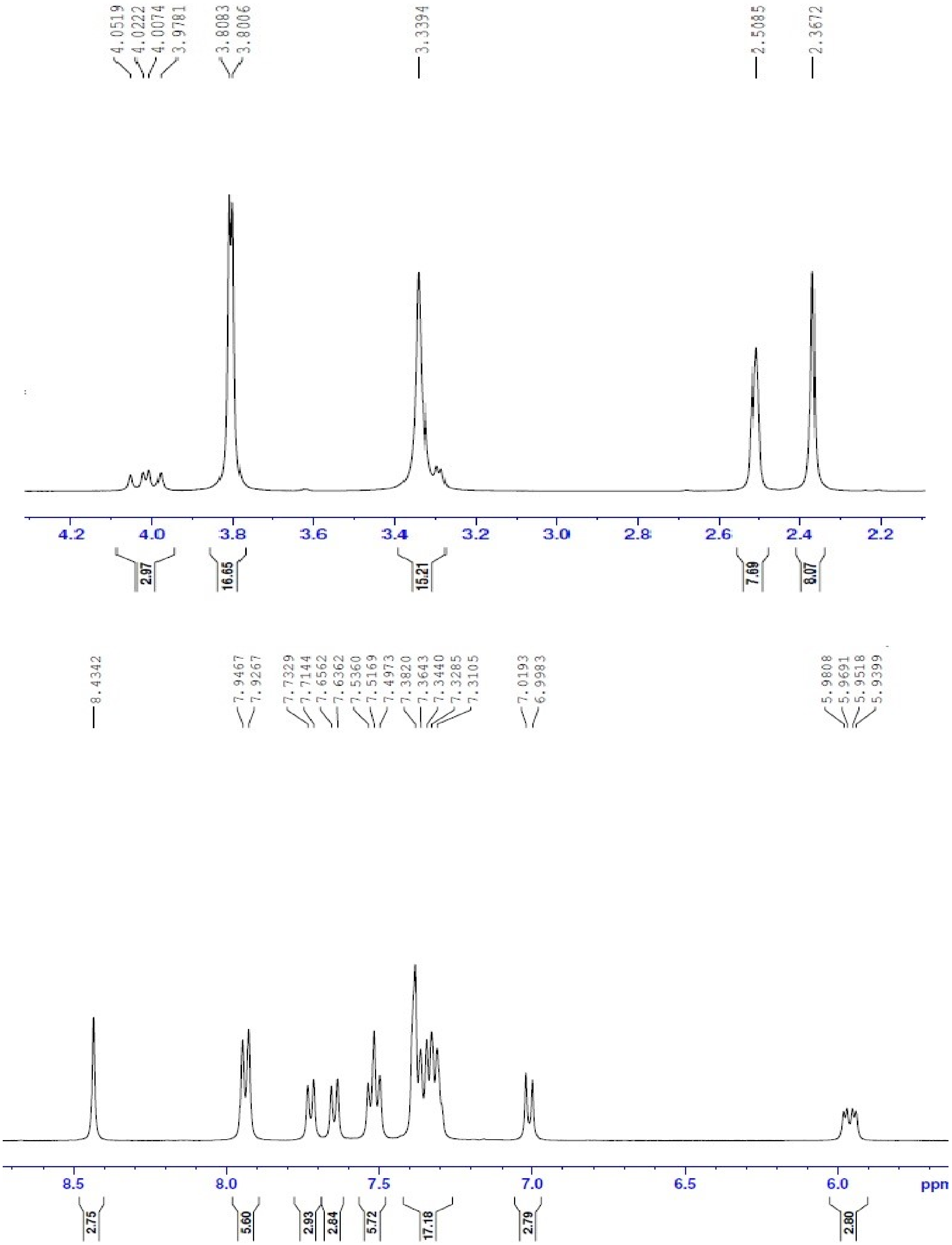

Figure S20: ^{1}HNMR (400 MHz; DMSO-*d6*) spectrum of compound **4c**

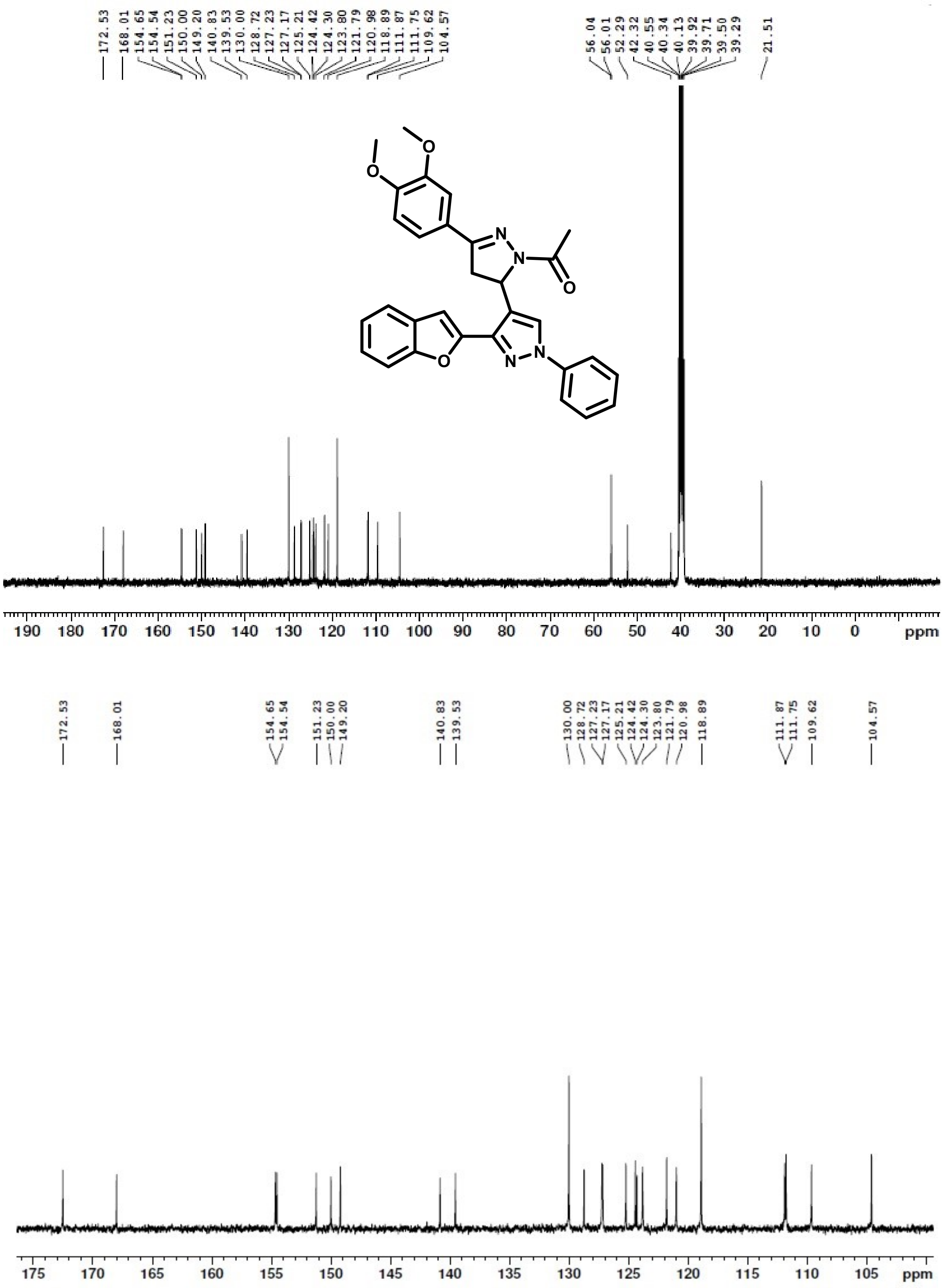

Figure S21: ^{13}CNMR (100 MHz; DMSO-*d6*) spectrum of compound **4c**

1-(5-(3-(Benzofuran-2-yl)-1-phenyl-1H-pyrazol-4-yl)-3-(1H-benzo[d]imidazol-2-yl)-4,5-dihydropyrazol-1-yl)ethanone (4d)

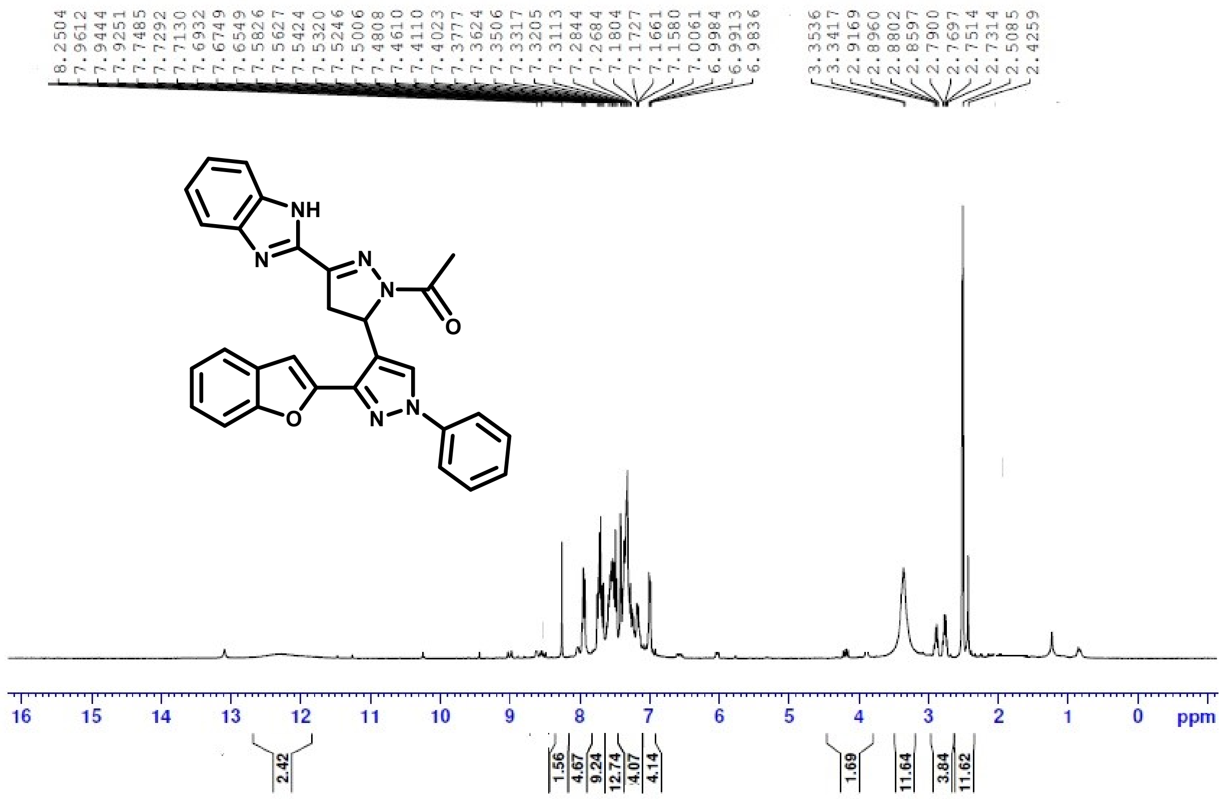

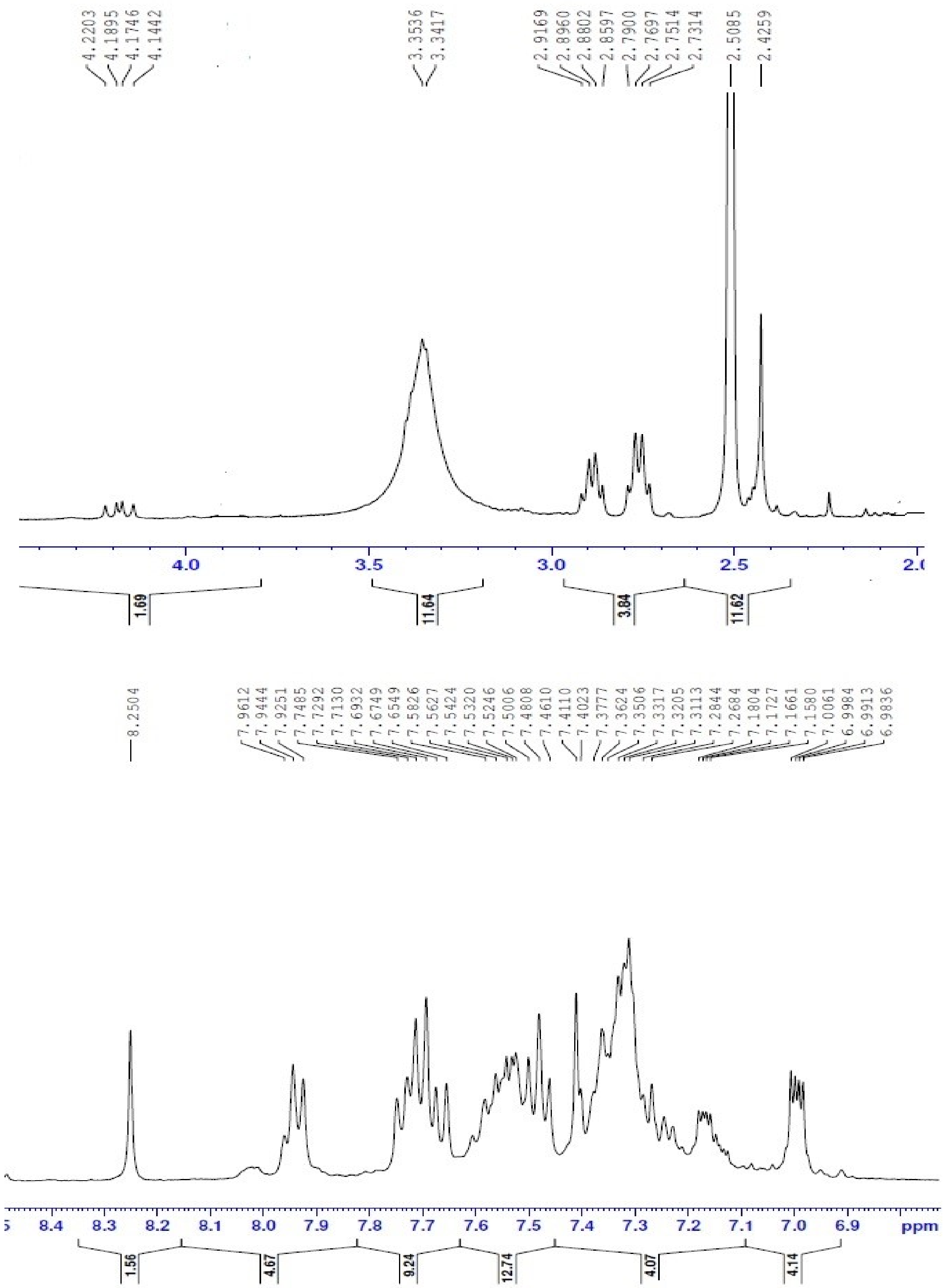

Figure S22: ^{1}HNMR (400 MHz; DMSO-*d6*) spectrum of compound **4d**

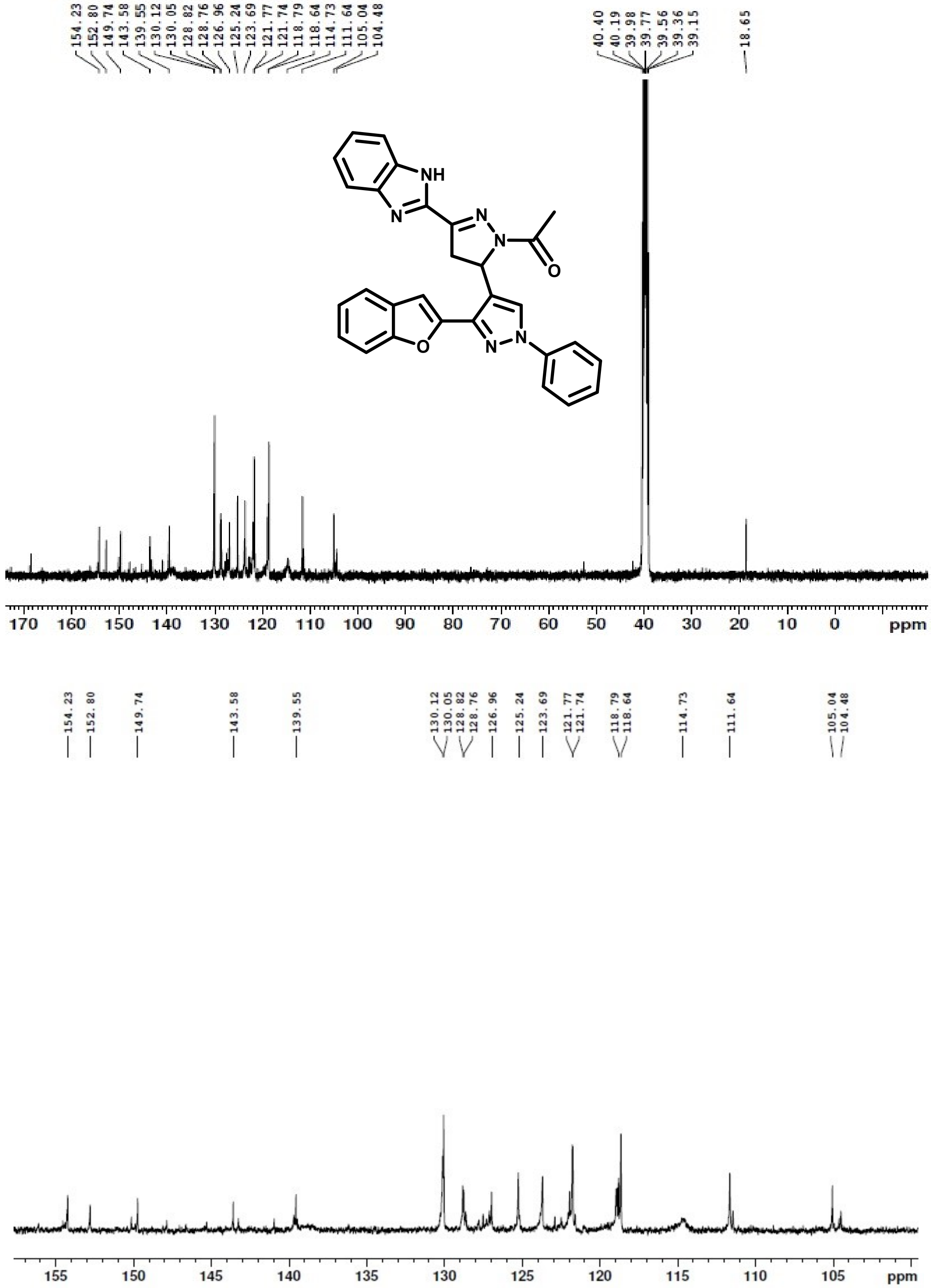

Figure S23: ^{13}CNMR (100 MHz; DMSO-*d6*) spectrum of compound **4d**

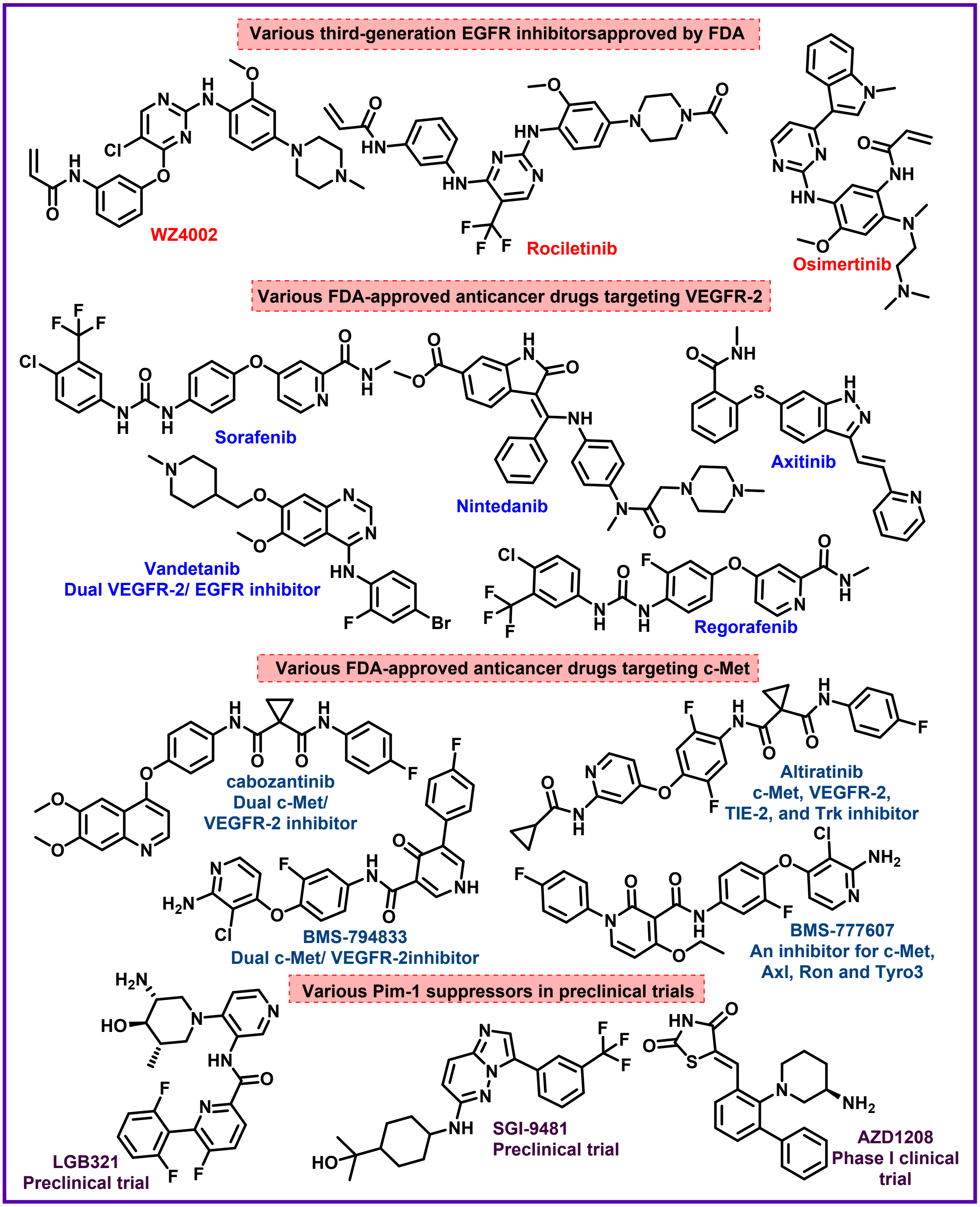

Figure S24: Examples of various anticancer drugs targeting EGFR, VEGFR-2, c-Met, and Pim-1 kinases

5.2. Biological activity

5.2.1. Evaluation of cytotoxic activity against a panel of sixty human cancer cell lines

The selected compounds by NCI were evaluated for their anticancer activity in a two-stage process. First, these compounds were screened against the full NCI 60 cell lines panel representing leukemia, Non-Small Cell Lung Cancer, melanoma, colon cancer, CNS cancer, breast cancer, ovarian cancer, renal cancer and prostate cancer at a single high dose of 10^{-5} M. Then, the output from the single dose screen is reported as a mean graph and is available for analysis by the COMPARE program. Second, compounds exhibiting significant growth inhibition were evaluated against the 60 cell panel at five different minimal concentrations (0.01, 0.1, 1, 10 and 100 μM).

5.2.1.1. Assay protocol

The human tumor cell lines of the cancer screening panel are grown in RPMI 1640 medium containing 5% fetal bovine serum and 2 mM L-glutamine. For a typical screening experiment, cells are inoculated into 96 well microtiter plates in 100 mL at plating densities ranging from 5000 to 40,000 cells/well depending on the doubling time of individual cell lines. After cell inoculation, the microtiter plates are incubated at 37 C, 5% CO_2, 95% air and 100% relative humidity for 24 h prior to addition of the experimental drugs. After 24 h, two plates of each cell line are fixed in situ with TCA, to represent a measurement of the cell population for each cell line at the time of drug-addition (Tz). The experimental drugs are solubilized in dimethyl sulfoxide at 400-fold the desired final maximum test concentration and stored frozen prior to use. At the time of drug addition, an aliquot of frozen concentrate is thawed and diluted to twice the desired final maximum test concentration with complete medium containing 50 mg/mL Gentamicin. Additional four, 10-fold or ½ log serial dilutions are made to provide a total of five drug concentrations plus control. Aliquots of 100 mL of these different drug dilutions are added to the appropriate microtiter wells already containing 100 mL of medium, resulting in the required final drug concentrations. Following drug addition, the plates are incubated for an additional 48 h at 37 ^{o}C, 5% CO_2, 95% air, and 100% relative humidity. For adherent cells, the assay is terminated by the addition of cold TCA. Cells are fixed in situ by the gentle addition of 50 ml of cold 50% (w/v) TCA (final concentration, 10% TCA) and incubated for 60 min at 4 ^{o}C. The supernatant is discarded, and the plates are washed five times with tap water and air dried. Sulforhodamine B (SRB) solution (100 mL) at 0.4% (w/v) in 1% acetic acid is added to each

well, and plates are incubated for 10 min at room temperature. After staining, unbound dye is removed by washing five times with 1% acetic acid and the plates are air dried. Bound stain is subsequently solubilized with 10 mM trizma base, and the absorbance is read on an automated plate reader at a wavelength of 515 nm. For suspension cells, the methodology is the same except that the assay is terminated by fixing settled cells at the bottom of the wells by gently adding 50 ml of 80% TCA (final concentration, 16% TCA).

5.2.1.2. Data analysis

Using the seven absorbance measurements [time zero, (Tz), control growth, (C), and test growth in the presence of drug at the five concentration levels (Ti)], the percentage growth is calculated at each of the drug concentrations levels. Percentage growth inhibition is calculated as: [(Ti–Tz)/(C–Tz)] x 100 for concentrations for which Ti P Tz and [(Ti-Tz)/Tz]x100 for concentrations for which Ti < Tz: three dose response parameters are calculated for each experimental agent. Growth inhibition of 50% (GI_{50}) is calculated from [(Ti–Tz)/(C–Tz)] x 100 = 50, which is the drug concentration resulting in a 50% reduction in the net protein increase (as measured by SRB staining) in control cells during the drug incubation. The LC_{50} (concentration of drug resulting in a 50% reduction in the measured protein at the end of the drug treatment as compared to that at the beginning) indicating a net loss of cells following treatment is calculated from [(Ti–Tz)/Tz] x 100 = -50. Values are calculated for each of these three parameters if the level of activity is reached; however, if the effect is not reached or is exceeded, the value for that parameter is expressed as greater or less than the maximum or minimum concentration tested. Results for each compound were reported as a mean graph of the percent growth of the treated cells when compared to the untreated control cells. There after obtaining the results for one dose assay, analysis of historical Development Therapeutics Programme (DTP) was performed and compounds which satisfies predetermined threshold inhibition criteria is selected for NCI full panel 5 dose assay.

5.2.2. Enzymatic assays

5.2.2.1. c-Met kinase inhibitory assay

The c-Met kinase activity was determined in 384-well plates using homogenous time-resolved fluorescence (HTRF) assays following the manufacturer's instructions. The compound 3d was dissolved in DMSO and diluted to different concentrations with kinase buffer. First, 4 µl of the compound solution, 2 µl of TK substrate solution (5 µM), 2 µl of c-met solution (0.3075

μg/ml), and LB943 (Bethold, Germany) were measured for fluorescence at 620 nm and 665 nm using the excitation light at 320 nm. 2 μl of ATP solution (15 μM) were successively added to each well. Reactions were incubated for 40 min at 37 ◦C, followed by the addition of 10 μl mixed solution containing 5 μl SA-XL665 (0.5 μM) and 5 μl TK Antibody, and sealing plate incubation for 1 h at 37 °C. The fluorescence at 620 nm and 665 nm was measured with Mithras LB943 (Bethold, Germany) using 100 us - [(activation light at 320 nm. The inhibition rate (%) was calculated using the following equation: % inhibition = 100 - [(activity of enzyme with tested compounds - min)/(max - min)] × 100 (max: the observed enzyme activity measured in the presence of enzyme, substrates, and cofactors; min: the observed enzyme activity in the presence of substrates and cofactors and in the absence of enzyme). IC_{50} values were processed by SPSS 19.0 statistical software from the inhibition curves. The experimental results were expressed by mean ± SD of three independent experiments.

5.2.2.2. EGFR^{WT} kinase inhibitory assay

The most active cytotoxic compound, **3d,** was further examined for its inhibitory activities against EGFR^{WT}. Homogeneous time-resolved fluorescence (HTRF) assay was applied in this test with EGFR (WT) (Sigma). Firstly, EGFR^{WT} and its substrates were incubated with the tested compounds in enzymatic buffer for 5 min. ATP (1.65 μM) was added into the reaction mixture to allow starting the enzymatic reaction. The assay was conducted for 30 min at room temperature. The reaction was stopped by the addition of detection reagents that contain EDTA. The detection step continued for 1 h, and then the IC_{50} values were determined using GraphPad Prism 5.0. Three independent experiments were performed for each concentration.

5.2.2.3. VEGFR-2 Kinase inhibitory assay

The most active cytotoxic compound, **3d**, was tested as a VEGFR-2 kinase inhibitor using the VEGFR-2 Kinase Assay Kit to measure VEGFR-2 kinase activity for screening and profiling applications using Kinase-Glo® MAX as a detection reagent.

The assay protocol: The sample and control were tested in duplicate. 1) 5x Kinase Buffer 1, ATP, and 50x PTK substrate were thawed (optional: If desired, DTT could be added to 5x Kinase Buffer 1 to make a 10 mM concentration; e.g., 10 μl of 1 M DTT could be added to 1 ml of 5x Kinase Buffer 1. 2) The master mixture (25 μl per well) was prepared as follows: N wells x (6 μl 5x Kinase Buffer 1 + 1 μl ATP (500 μM) + 1 μl 50x PTK substrate + 17 μl water). 25 μl were added to every well. 3) 5 μl of inhibitor solution were added to each well labeled as "Test

Inhibitor.”. For the “Positive Control" and “Blank,” 5 µl of the same solution were added without inhibitor (inhibitor buffer). 4) 3 mL of 1x Kinase Buffer 1 were prepared by mixing 600 µL of 5x Kinase Buffer 1 with 2400 µL of water. 3 ml of 1x Kinase Buffer 1 is sufficient for 100 reactions. 5) To the wells designated as "Blank," 20 µl of 1x Kinase Buffer 1 were added. 6) The VEGFR-2 enzyme was thawed on ice. Upon first thaw, briefly the tube containing the enzyme was spun to recover the full content of the tube. The amount of VEGFR-2 required for the assay was calculated, and the enzyme was diluted to 1 ng/µl with 1x Kinase Buffer 1. remaining undiluted enzyme was stored in aliquots at -80°C. 7) The reaction was initiated by adding 20 µl of diluted VEGFR2 enzyme to the wells designated “Positive Control” and "Test Inhibitor Control." And incubated at 30°C for 45 minutes. 8) Kinase-Glo Max reagent was thawed. 9) After the 45 minutes, 50 µl of Kinase-Glo Max reagent was added to each well. The plate was covered with aluminum foil and incubated at room temperature for 15 minutes. 10) Luminescence was measured using the microplate reader according to the manufacturer’s instructions.

5.2.2.4. B-Raf Kinase inhibitory assay

The activity of **3d** was screened against B-Raf. All samples and controls were tested in duplicate. The assay protocol: 1) 5x Kinase Buffer 1, ATP, and 5x Raf substrate were thawed. 2) The master mixture was prepared (25 µl per well): N wells x (6 µl 5x Kinase Buffer 1 + 1 µl ATP (500 µM) + 10 µl 5X Raf substrate + 8 µl water). 25 µl was added to every well. 3) 5 µl of inhibitor solution of each well was added and labeled as “Test Inhibitor.”. For the “Positive Control" and “Blank,” 5 µl of the same solution was added without inhibitor (inhibitor buffer). 4) 3 ml of 1x Kinase Buffer 1 was prepared by mixing 600 µl of 5x Kinase Buffer 1 with 2400 µl of water. 3 ml of 1x Kinase Buffer 1 is sufficient for 100 reactions. 5) To the wells designated as "Blank," 20 µl of 1x Kinase Buffer 1 was added. 6) B-Raf (V600E) enzyme was thawed on ice. Upon first thaw, the tube containing the enzyme was spun to recover the full content of the tube. The amount of B-Raf required for the assay was calculated, and the enzyme to ~2 ng/µl for B-Raf (V600E) was diluted with 1x Kinase Buffer 1. The remaining undiluted enzyme was stored in aliquots at -80°C. 7) The reaction was initiated by adding 20 µl of diluted B-Raf(V600E) enzyme to the wells designated “Positive Control” and "Test Inhibitor Control," and incubation was carried out at 30°C for 45 minutes. 8) Kinase-Glo Max reagent was thawed. 9) After the 45-

minute reaction, 50 µl of Kinase-Glo Max reagent was added to each well. The plate was covered with aluminum foil and incubated at room temperature for 15 minutes. 10) Luminescence was measured using the microplate reader.

5.2.2.5. PIM kinase inhibitory assay

The most active cytotoxic compound, **3d,** was further examined for its inhibitory activity against the PIM enzyme using the ADP-Glo™ Kinase Assay.

The protocol of the experiment depends on the dilution of the enzyme, substrate, ATP, and inhibitors in kinase buffer. Then, 1 µl of the inhibitor (5% DMSO), 2 µl of the enzyme, and 2 µl of the substrate/ATP were added to the wells of the 384 low-volume plate and incubated at room temperature for 60 minutes. Then, 5 µl of ADP-Glo™ Reagent was added and incubated at room temperature for 40 minutes. 10 µl of the kinase detection reagent was added and incubated at room temperature for 30 minutes. Then, luminescence (integration time 0.5–1 second) was recorded according to the manufacturer's instructions.

5.2.3. Cell cycle analysis

Further exploration of the cytotoxic activity of compound **3d** was performed using propidium iodide (PI) flow cytomertric analysis to measure the extent of PI that binds to DNA of dead cells with permeable plasma membranes to determine cell cycle status in tissue culture quantitate cell death at all cell phases. After incubation of MCF-7 cells with compound **3d**, cells were fixed with ethanol then dehydrated before staining with PI according to the reported methodology.

5.2.4. Annexin-V FTIC apoptotic study

Estimation of fractional DNA content (aka sub-G1 assay) is a widely used assay to determine apoptosis. Cleavage of genomic DNA into smaller fragments (180–200 bp lengths) is a hallmark for apoptosis in numerous cells. PI stained cells will stain less intensely and show a peak below the G1 peak (Sub-G1). In this work, flow cytometer with Annexin V-fluorescein isothiocyanate/propidium iodide FITC/PI double staining apoptosis detection kit (K101, Biovision) was used to study apoptosis of MCF-7 cells treated with compound **3d**. Cells were incubated, collected by centrifugation, re-suspended in 500 µL of 1X binding buffer. Annexin V-FITC (5 µL) and PI (5 µL) was added and incubation was continued for additional 5 min in the dark at room temperature. Annexin V-FITC binding was analyzed by flow cytometry using FITC signal detector (FL1) and PI staining by FL2 phycoerythrin emission signal detector [75].
